# Anti-Inflammatory Effects of Bisacurone Isolated from *Curcuma longa* (Ryudai Gold): An In Vivo and In Silico Study

**DOI:** 10.3390/molecules31030548

**Published:** 2026-02-04

**Authors:** Mahir Anjum, Md. Amzad Hossain, Jesmin Akter, Atsushi Miyamoto, Md. Zahorul Islam

**Affiliations:** 1 Department of Pharmacology, Faculty of Veterinary Science, Bangladesh Agricultural University, Mymensingh 2202, Bangladesh; mahir47537@bau.edu.bd; 2 Faculty of Agriculture, University of the Ryukyus, Nishihara 903-0213, Okinawa, Japan; jesminbau02@gmail.com; 3 Department of Veterinary Pharmacology, Joint Faculty of Veterinary Medicine, Kagoshima University, 1-21-24 Korimoto, Kagoshima City 890-0065, Kagoshima, Japan; k1330977@kadai.jp

**Keywords:** bisacurone, *Curcuma longa*, in vivo, in silico, anti-inflammatory, histopathology

## Abstract

Bisacurone is a sesquiterpenoid constituent of *Curcuma longa* that has received considerably less attention than curcuminoids despite emerging evidence of its biological activity. In this study, the anti-inflammatory potential of bisacurone isolated from the Ryudai gold variety of *Curcuma longa* was evaluated using an integrated in vivo and in silico approach. Acute inflammation was assessed in rats using a carrageenan-induced paw edema model, supported by histopathological examination of paw tissues. Bisacurone significantly reduced paw edema during the peak inflammatory phase and markedly attenuated dermal thickening and inflammatory cell infiltration, indicating effective suppression of acute inflammatory responses. The effects of bisacurone were comparable to that of indomethacin. To elucidate the underlying molecular basis, density functional theory calculations, molecular docking, molecular dynamics simulations, and pharmacokinetic and toxicity predictions were performed. In silico analyses revealed favorable electronic properties, drug-likeness, and stable interactions of bisacurone with key inflammatory regulators, particularly IKKβ and COX-1, along with moderate interactions with MAPKs and iNOS. Molecular dynamics simulations confirmed the stability of the protein–ligand complexes. Collectively, these findings demonstrate that bisacurone exerts anti-inflammatory effects through multi-target modulation of inflammatory signaling pathways and highlight its potential as a bioactive functional food component and a lead compound for anti-inflammatory drug development.

## 1. Introduction

Inflammation is a fundamental biological response that protects tissues against infection, injury, and metabolic stress, thereby maintaining homeostasis [1]. However, persistent or dysregulated inflammatory signaling is a key contributor to the pathogenesis of chronic disorders, including metabolic syndrome, cardiovascular disease, neurodegenerative conditions, cancer, and progressive tissue degeneration [2,3,4]. At the molecular level, chronic inflammation is characterized by sustained activation of transcription factors, stress-responsive kinases, and redox-sensitive enzymes, leading to excessive cytokine production, oxidative stress, and cumulative cellular damage [5,6]. Although conventional anti-inflammatory therapies such as non-steroidal anti-inflammatory drugs and glucocorticoids are effective, their long-term use is limited by adverse effects, immunosuppression, and reduced efficacy [7,8]. Moreover, inflammatory signaling is governed by interconnected networks involving nuclear factor kappa B (NF-κB), mitogen-activated protein kinases (MAPKs), cyclooxygenases (COX), inducible nitric oxide synthase (iNOS), and oxidative stress regulators, highlighting the limitations of single-target interventions and the need for multi-target modulators [9].

Natural products have, therefore, gained increasing attention as sources of structurally diverse compounds capable of modulating multiple inflammatory pathways simultaneously. Numerous phytochemicals exert coordinated effects on redox balance, kinase signaling, and transcriptional regulation, making them attractive candidates for the management of chronic inflammatory conditions [10,11]. Among medicinal plants, *Curcuma longa* L. (turmeric) has been extensively studied due to its long history of dietary consumption and broad pharmacological activity. While curcumin has dominated turmeric research, accumulating evidence demonstrates that turmeric contains a wide range of non-curcuminoid constituents, including sesquiterpenoids and volatile compounds, whose biological activities remain comparatively underexplored [12,13,14,15].

**Figure 1:**
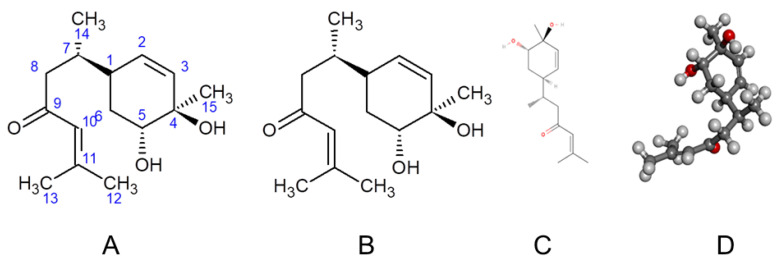
Structural representation of bisacurone. (**A**) 2D chemical structure showing atom numbering; (**B**) 2D structure highlighting stereochemistry. (**C**) 2D skeletal formula. (**D**) 3D ball-and-stick model illustrating the spatial conformation of the molecule.

We developed the Ryudai gold variety of *Curcuma longa*, registered by the Ministry of Agriculture, Forestry, and Fisheries of Japan (Registration No. 21485, 29 February 2012). In our previous work, bioactivity-guided fractionation of this variety led to the isolation and structural characterization of several compounds, including bisacurone [16]. Although bisacurone was successfully isolated, it exhibited relatively weak antioxidant activity and was, therefore, excluded from the antioxidant-focused analysis of that study [16]. Subsequent studies, however, have revealed that bisacurone, a bisabolane-type sesquiterpenoid (Figure 1), exerts biological effects that extend beyond direct radical scavenging. In contrast to curcumin, bisacurone displays modest antioxidant capacity, suggesting that its anti-inflammatory activity is mediated predominantly through specific protein-level interactions [17]. We have isolated and purified 39.6 mg bisacurone (1.32%) after repeated fractionation from 3 kg fresh rhizome of Ryudai gold [16]. This concentration may not be significant as an anti-inflammatory dose when using this turmeric as a spice. Therefore, isolated sesquiterpene could be explored as potential lead compound for the development of anti-inflammatory drug.

Indeed, in vitro studies demonstrate that bisacurone suppresses lipopolysaccharide- and cytokine-induced inflammatory responses by inhibiting IκB kinase activation and NF-κB nuclear translocation, resulting in reduced expression of pro-inflammatory cytokines and adhesion molecules [17,18]. At the molecular level, several pathways appear repeatedly. These include NF-κB, MAPKs, TLR4-linked cascades, Nrf2/Keap1 axis, JNK1, and iNOS [17,18]. In vivo investigations further indicate that bisacurone attenuates inflammation and tissue injury in experimental models of diabetic nephropathy, myocardial ischemia–reperfusion injury and burn wounds through coordinated modulation of NF-κB signaling, activation of the Nrf2/heme oxygenase-1 pathway, and attenuation of oxidative and endoplasmic reticulum stress [19,20,21]. Despite these findings, the molecular basis of bisacurone’s anti-inflammatory activity remains incompletely defined, as most studies infer pathway involvement from downstream biomarker changes without establishing direct interactions with key inflammatory proteins. To address this gap, this study employs an integrated in vivo and in silico approach, combining a carrageenan-induced paw edema model in rats with computational analyses—including density functional theory calculations, pharmacokinetic and toxicity prediction, molecular docking, and molecular dynamics simulations—to elucidate the molecular interactions of bisacurone with the major inflammatory regulators. This integrated strategy provides mechanistic insight into bisacurone as a multi-target modulator of inflammatory signaling and supports its potential relevance in inflammation-driven pathologies.

## 2. Results

### 2.1. Effects of Bisacurone on Carrageenan-Induced Paw Edema in Rat

**Figure 2:**
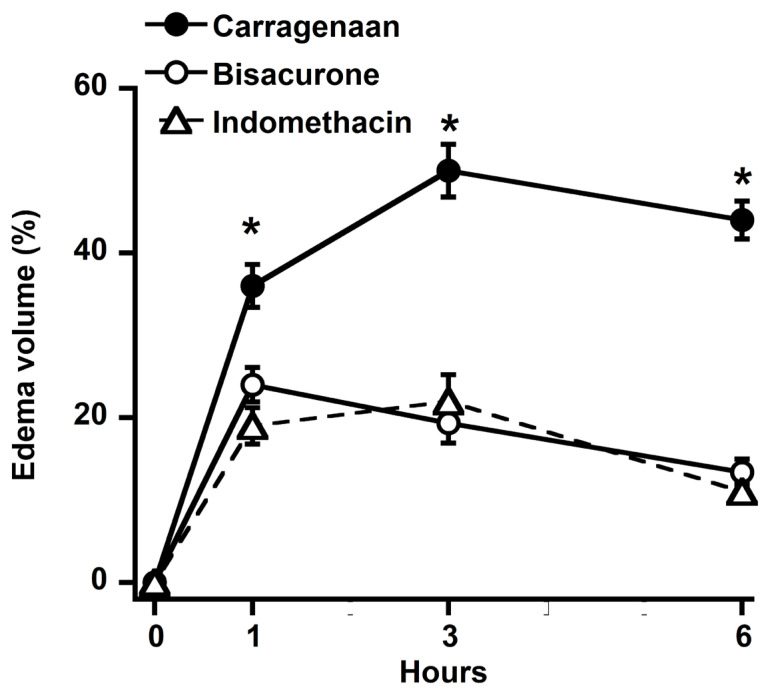
Effect of bisacurone (○; 100 µg/kg body weight, oral gavage) and indomethacin (Δ; 10 mg/kg bwt, oral gavage) on carrageenan-induced paw edema in rats (●). Both bisacurone and indomethacin treatment produced a significant reduction in paw swelling compared with the carrageenan control. Each value represents the mean ± SD of four rats. * *p*-value < 0.05 versus carrageenan-treated group.

Carrageenan administration caused a clear increase in paw volume. Bisacurone treatment (100 µg/kg body weight) produced a significant reduction in paw edema compared with the carrageenan control (Figure 2). Paw swelling peaked 3 h after carrageenan injection, reaching 50.0 ± 3.2% in the control group. In contrast, treatment with bisacurone or indomethacin markedly attenuated edema formation, resulting in increases of 19.3 ± 2.4% and 22.0 ± 3.1%, respectively. The maximum anti-inflammatory effect of bisacurone appeared at the third hour after carrageenan injection. At six hours, the inhibitory effect on edema volume declined (Figure 2). Notably, bisacurone exhibited an edema-inhibitory effect comparable to indomethacin (10 mg/kg), with no statistically significant difference between the two treatments.

### 2.2. Histopathological Analysis of Rat Paws Tissue

**Figure 3:**
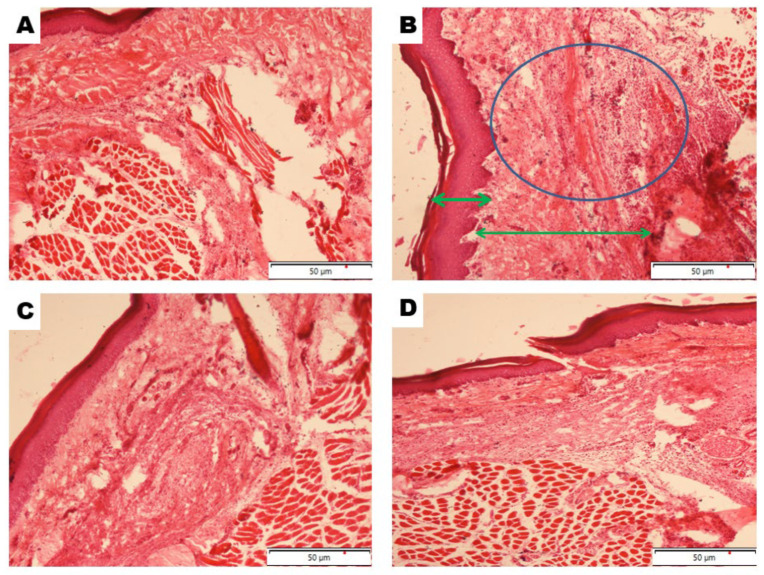
Representative histological sections of rat paw tissue collected three hours after carrageenan injection and stained with hematoxylin and eosin (original magnification = ×100; scale bar = 50 μm). (**A**) Control group showing normal dermal and hypodermal structure. (**B**) Carrageenan-treated group exhibiting acute inflammatory responses extending into the muscle layer with extensive inflammatory cell accumulation (blue circle) and thickening of dermis and epidermis (green arrows). (**C**) Carrageenan + bisacurone and (**D**) carrageenan + indomethcin groups showing a reduced number of infiltrating inflammatory cells and partial restoration of normal tissue structure.

Microscopic evaluation of paw sections from the control rats showed a well-preserved tissue structure with no visible signs of inflammation (Figure 3A). In contrast, carrageenan administration produced clear pathological changes, including heavy infiltration of inflammatory cells, prominent edema, thickening of the dermal layer, and disruption of normal tissue organization (Figure 3B). Treatment with bisacurone (100 μg/kg body weight) noticeably improved these changes. Three hours after carrageenan injection, bisacurone markedly reduced dermal and epidermal thickening and limited inflammatory cell infiltration, leading to a partial recovery of the normal tissue architecture (Figure 3C). A comparable protective effect was observed in the indomethacin-treated group (Figure 3D).

### 2.3. Results of In Silico Studies

#### 2.3.1. Conceptual Density Functional Theory (DFT)

**Figure 4:**
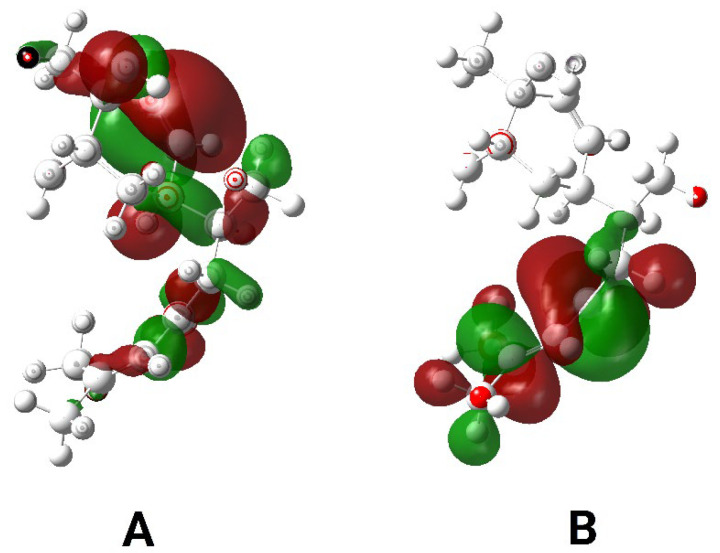
Frontier molecular orbital surfaces of bisacurone. (**A**) Highest Occupied Molecular Orbital (HOMO); (**B**) Lowest Unoccupied Molecular Orbital (LUMO). Red and green isosurfaces represent the positive and negative phases of the molecular orbital wave function, respectively.

Density Functional Theory was used to calculate the frontier molecular orbital energies of bisacurone [Supplementary Materials]. The highest occupied molecular orbital represents the ability of the molecule to donate electrons. The lowest unoccupied molecular orbital represents its ability to accept electrons (Figure 4). The E_HOMO_ value was −6.754 eV. The E_LUMO_ value was −1.464 eV. These values produced an energy gap of 5.29 eV (Table 1). This gap indicates moderate electronic stability with acceptable chemical reactivity.

The dipole moment of bisacurone was 6.518 Debye. This value shows clear molecular polarity and a strong tendency to interact with biological targets. The dipole moment remains slightly lower than the 7.0 Debye level that usually indicates very high reactivity. Even so, bisacurone still shows notable chemical activity.

Conceptual DFT parameters were calculated from the E_HOMO_ and E_LUMO_ values. The electronegativity (χ) of bisacurone was 4.109 eV. The chemical potential (μ) showed a value of −4.109 eV. The absolute hardness (η) reached 2.645 eV. The global softness (σ) reached 0.378 eV^−1^. These values suggest balanced resistance to charge transfer and moderate polarizability. The electrophilicity index (ω) was 3.19 eV (Table 1). This result indicates a moderate ability to accept electrons.

#### 2.3.2. Molecular Docking Studies

**Figure 5:**
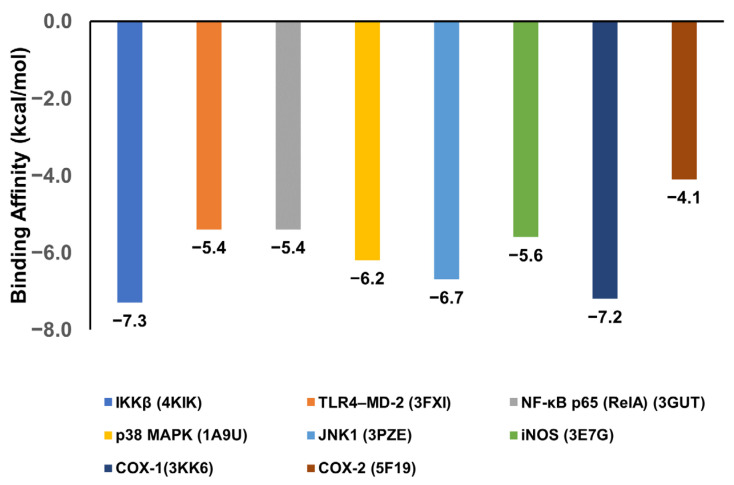
Docking scores of bisacurone with receptors.

**Figure 6:**
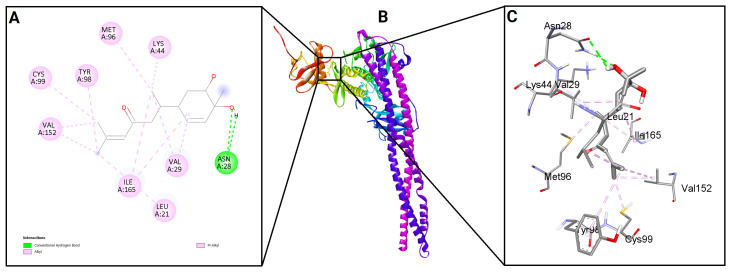
2D (**A**) and 3D (**C**) views of interaction and binding pose (**B**) of bisacurone with IKKβ (4KIK).

**Figure 7:**
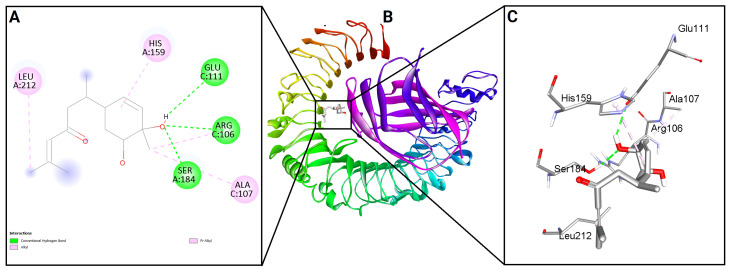
2D (**A**) and 3D (**C**) views of interaction and binding pose (**B**) of bisacurone with TLR4–MD-2 (3FXI).

**Figure 8:**
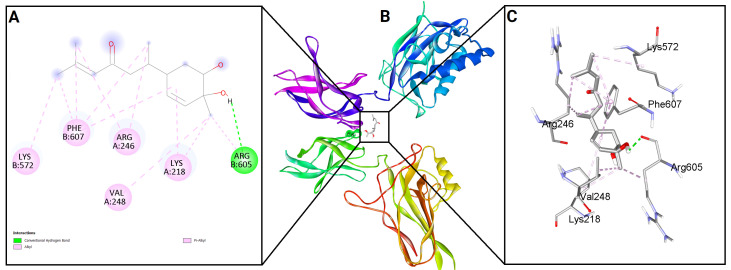
2D (**A**) and 3D (**C**) views of interaction and binding pose (**B**) of bisacurone with NF-κB p65 (RelA) (3GUT).

**Figure 9:**
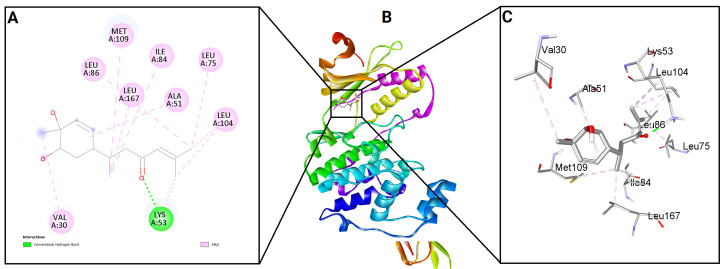
2D (**A**) and 3D (**C**) views of interaction and binding pose (**B**) of bisacurone with p38 MAPK (1A9U).

**Figure 10:**
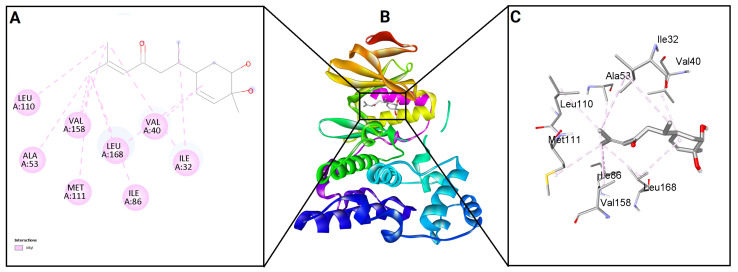
2D (**A**) and 3D (**C**) views of interaction and binding pose (**B**) of bisacurone with JNK1 (3PZE).

**Figure 11:**
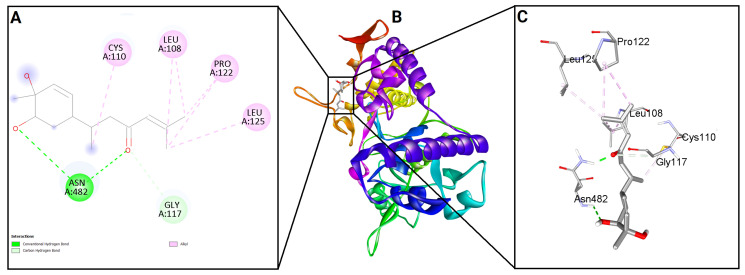
2D (**A**) and 3D (**C**) views of interaction and binding pose (**B**) of bisacurone with iNOS (3E7G).

**Figure 12:**
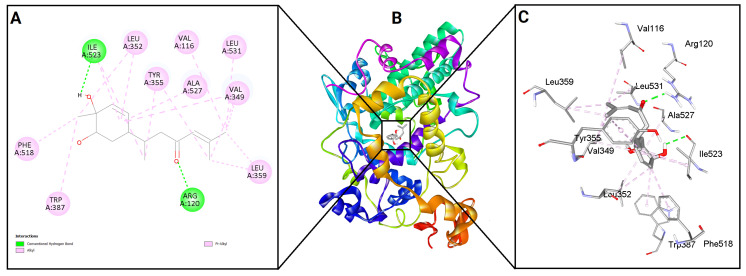
2D (**A**) and 3D (**C**) views of interaction and binding pose (**B**) of bisacurone with COX-1 (3KK6).

**Figure 13:**
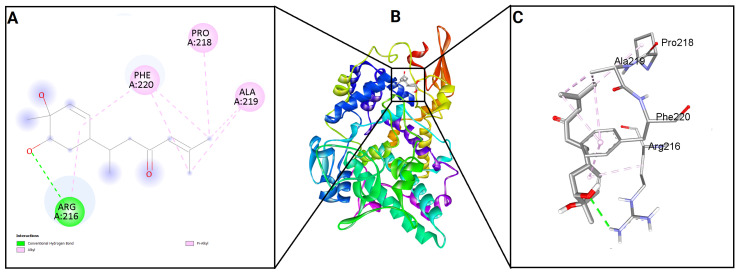
2D (**A**) and 3D (**C**) views of interaction and binding pose (**B**) of bisacurone with COX-2 (5F19).

To evaluate the binding potential of bisacurone, molecular docking was performed against eight key inflammatory receptors: IKKβ, TLR4–MD-2, NF-κB p65 (RelA), p38 MAPK, JNK1, iNOS, COX-1, and COX-2 (Figure 5). The docking poses and interactions for all receptors and ligands are shown in Figure 6, Figure 7, Figure 8, Figure 9, Figure 10, Figure 11, Figure 12 and Figure 13. Bisacurone exhibited the strongest binding affinity with IKKβ (−7.3 kcal/mol) and COX-1 (−7.2 kcal/mol), forming two hydrogen bonds with each. Moderate binding energies were observed for p38 MAPK (−6.2 kcal/mol), JNK1 (−6.7 kcal/mol), and iNOS (−5.6 kcal/mol), with iNOS forming three hydrogen bonds involving ASN482 and GLY117. TLR4–MD-2 and NF-κB p65 showed binding energies of −5.4 kcal/mol, with TLR4–MD-2 forming three hydrogen bonds and NF-κB p65 forming one. COX-2 displayed the lowest binding energy (−4.1 kcal/mol) with a single hydrogen bond to ARG216. Table 2 summarizes the docking scores, hydrogen bond interactions, and key amino acid residues involved in ligand binding.

#### 2.3.3. In Silico Pharmacokinetics and Toxicity Analysis

Bisacurone adheres to all major drug-likeness criteria, including Lipinski’s, Veber’s, Ghose’s, Egan’s, and Muegge’s rules. Its molecular weight remains well below the 500 g/mol threshold, supporting favorable absorption and distribution properties. The compound features two hydrogen-bond donors and three hydrogen-bond acceptors, both comfortably within the recommended limits for oral bioavailability.

Lipophilicity values, as measured by iLOGP and MLOGP, fall within the acceptable range, indicating balanced hydrophilic–lipophilic behavior conducive to membrane permeability. The water solubility prediction also supports its potential for oral administration. With only four rotatable bonds, bisacurone meets Veber’s criteria for molecular flexibility, further enhancing its drug-like profile.

Importantly, the compound shows zero violations across all five drug-likeness filters, suggesting a structurally sound and pharmacokinetically favorable candidate for further development (Table 3).

The skin permeation value (Log Kp) reflects how easily a compound can penetrate the skin barrier. Bisacurone shows a Log Kp of −6.5 cm/s, indicating low skin permeability. Swiss ADME also provides insights into absorption and distribution. Bisacurone demonstrates high gastrointestinal absorption and is capable of crossing the blood–brain barrier. It does not act as a P-glycoprotein substrate, which supports its potential for effective cellular uptake. Regarding metabolism, bisacurone does not inhibit any of the major cytochrome P450 enzymes, including CYP1A2, CYP2C19, CYP2C9, CYP2D6, and CYP3A4. This suggests a low risk of metabolic interference and drug–drug interactions. Overall, the compound exhibits favorable ADME characteristics with minimal inhibitory effects and strong absorption potential (Table 4).

Toxicity predictions for bisacurone from ProTox-III indicate a favorable safety profile. The compound has an LD_50_ of 1170 mg/kg and is categorized under Toxicity Class 4, which corresponds to moderate toxicity. Despite this classification, bisacurone remains inactive across all tested organ-specific toxicity endpoints. It shows no signs of hepatotoxicity, carcinogenicity, immunotoxicity, mutagenicity, cytotoxicity, nephrotoxicity, respiratory toxicity, or cardiotoxicity. These results suggest that bisacurone poses minimal risk for systemic toxicity and may be considered safe for further pharmacological evaluation. The findings are summarized in Table 5.

#### 2.3.4. Molecular Dynamics Simulation

**Figure 14:**
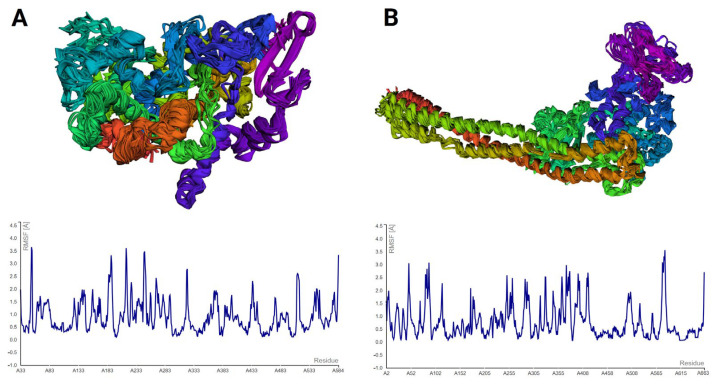
The superimposed multi-model structures from the simulation and the RMSF profiles from MD analysis are shown for (**A**) COX-1 and (**B**) IKKβ.

**Figure 15:**
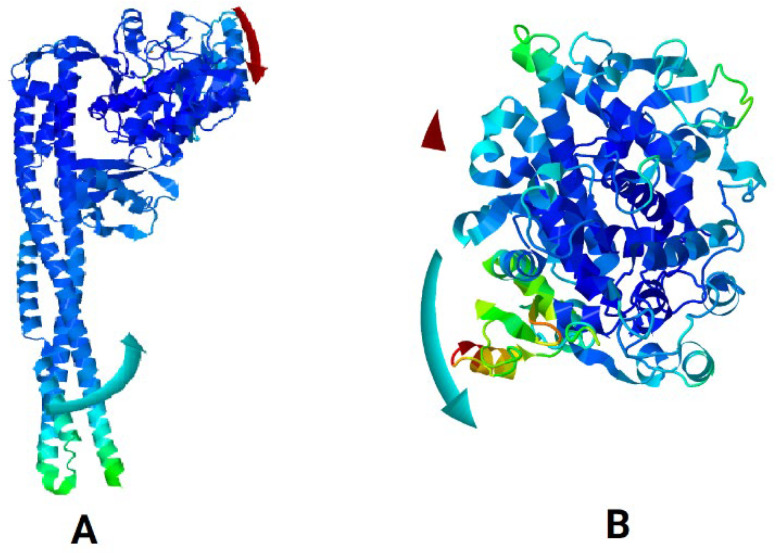
Molecular mobility of the docked complexes was assessed using NMA: (**A**) IKKβ–bisacurone and (**B**) COX-1–bisacurone. Colored affine arrows represent the direction and extent of motion, with longer arrows indicating higher mobility.

**Figure 16:**
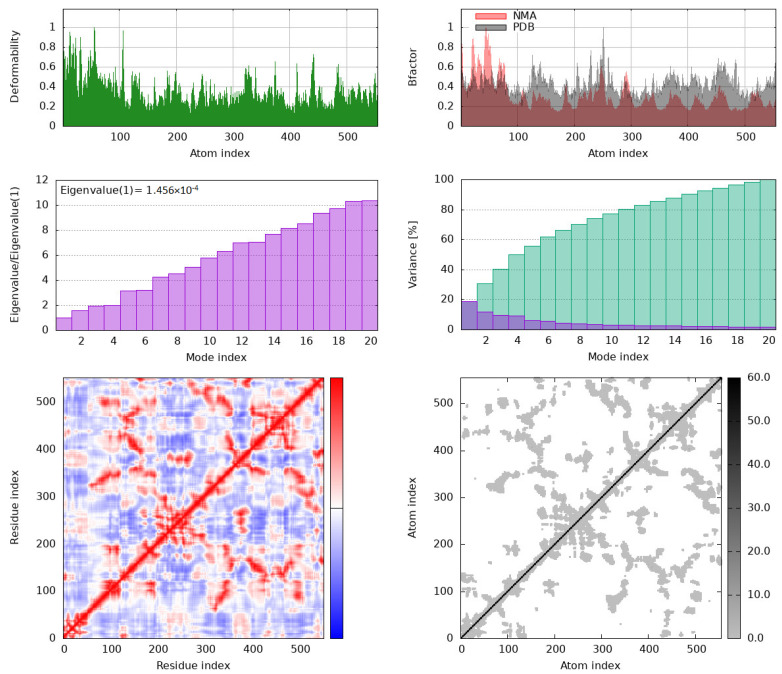
iMODS-based molecular dynamics simulation outputs for the COX-1–bisacurone complex. Deformability plot indicating the relative flexibility of each atom index. Comparison of B-factors derived from NMA (red) and the experimental PDB structure (gray). Eigenvalues associated with each combined mode, where the first mode eigenvalue is 1.456 × 10^−4^. Individual (purple) and cumulative (green) variance percentages for the first 20 modes. Covariance matrix illustrating correlated (red), uncorrelated (white), and anti-correlated (blue) motions between residue pairs.

**Figure 17:**
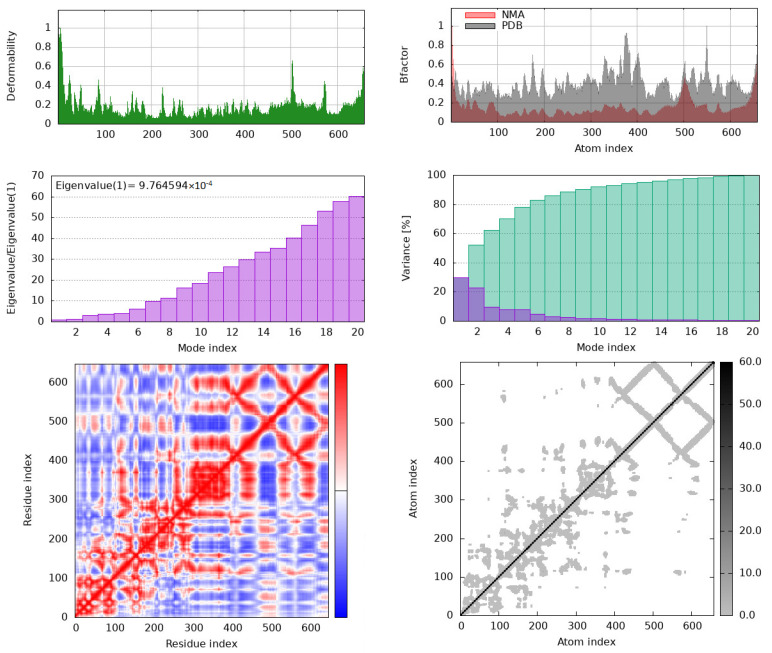
iMODS-based molecular dynamics simulation outputs for the IKKβ–bisacurone complex.

Molecular dynamics (MD) simulations were done to study the stability of protein–ligand complexes. The root mean square fluctuation (RMSF) profiles of IKKβ and COX-1 bound to bisacurone were calculated using CABS-flex. Higher RMSF values indicate more flexibility. Lower values show limited movement. The protein structures in PDB format were submitted to CABS-flex with default settings. The server produced 10 modeled structures and a graph of RMSF values for each residue in the complexes (Figure 14). In IKKβ, the highest fluctuation occurred at residue 583 (3.56 Å). The lowest fluctuation occurred at residue 388 of chain A (0.064 Å). In COX-1, the highest fluctuation occurred at residue 52 (3.646 Å). The lowest fluctuation occurred at residue 199 of chain A (0.104 Å) [Supplementary Materials]. The iMOD server was used for MD simulations to study the movement and stability of the docked complexes. Normal mode analysis (NMA) was performed to observe large-scale movements in the structures. Both COX-1–bisacurone and IKKβ–bisacurone complexes showed similar behavior. The backbones were stable, and hinge regions were flexible (Figure 15). In COX-1, deformability peaks indicated hinge regions that allow the protein to adapt during ligand binding while the backbone remained stable (Figure 16). IKKβ also exhibited hinge points with a strong, rigid core. B factor analysis showed higher mobility in loops and terminal regions, consistent with experimental data, although minor differences appeared in unresolved areas (Figure 17). Eigenvalue analysis gave low values for both complexes (COX-1: 1.456786 × 10^4^; IKKβ: 9.764594 × 10^6^). Low values indicate that the complexes can change shape with little energy. The first ten normal modes explained most of the movement. Covariance maps showed that residues near the binding sites moved together, supporting ligand binding. Residues in distant regions moved in opposite ways to balance the structure. Elastic network models confirmed that the cores and binding pockets were rigid. Outer regions were more flexible. These results show that both complexes keep a balance between stability and flexibility. The proteins remain stable while allowing structural adjustment. This balance helps with strong ligand binding and proper protein function.

## 3. Discussion

This study demonstrates that bisacurone exerts significant anti-inflammatory activity through a coordinated in vivo and in silico mechanism. In the carrageenan-induced paw edema model, bisacurone, a sesquiterpenoid isolated from *Curcuma longa* (Ryudai gold), markedly reduced paw swelling at the peak inflammatory phase and preserved normal histoarchitecture by limiting dermal thickening and inflammatory cell infiltration, indicating effective suppression of acute inflammatory progression. Although indomethacin and bisacurone produced similar levels of edema inhibition, bisacurone may be considered a superior candidate due to its natural bioactive origin and multi-target mechanistic profile with a potentially improved safety margin. The in vivo results are mechanistically supported by the in silico analyses, which revealed stable and energetically favorable interactions of bisacurone with key regulators of inflammatory signaling, particularly IKKβ and COX-1, along with moderate affinity toward MAPKs and iNOS. These key regulators of inflammation play a crucial role in the production of pro-inflammatory factors such as IL-1β, IL-6, TNFα, COX2, and iNOS [17,18,19,20,21]. Bisacurone has been shown to reduce the levels of these inflammatory mediators by inhibiting the aforementioned pathways [17,18,19,20,21]. Our in silico findings further support the mechanistic plausibility, offering comprehensive validation across all inflammatory models.

The carrageenan-induced paw edema model is a well-established experimental system for evaluating acute inflammatory responses driven by mediator-dependent vascular permeability and leukocyte recruitment [1,3]. Carrageenan-induced inflammation exhibits a biphasic response, with an early phase mediated by histamine, serotonin, and bradykinin, followed by a late phase sustained by prostaglandins, nitric oxide, and pro-inflammatory cytokines [4,8]. In this study, bisacurone exerted maximal inhibition during the peak inflammatory window, indicating preferential modulation of late-phase inflammatory mechanisms. This temporal profile suggests that bisacurone acts downstream of initial mediator release and interferes with regulatory pathways responsible for inflammatory amplification and persistence. Histopathological analysis corroborated these functional findings. Carrageenan challenge produced pronounced dermal thickening, interstitial edema, and dense inflammatory cell infiltration, reflecting active leukocyte recruitment and tissue disruption. Bisacurone treatment markedly reduced inflammatory cell infiltration and preserved tissue architecture, indicating suppression of cellular inflammatory responses rather than simple attenuation of exudative edema. Because excessive leukocyte recruitment amplifies cytokine signaling and perpetuates tissue damage, regulation of this process represents a critical control point in inflammation [9]. Comparable in vivo anti-inflammatory effects of bisacurone have been reported in other experimental models, supporting the biological relevance of the present findings. Yang et al. (2022) demonstrated that bisacurone significantly attenuated renal inflammation in a rat model of diabetic nephropathy, accompanied by reduced expression of pro-inflammatory cytokines and suppression of NF-κB activation [19]. Similarly, Yan et al. (2023) reported that topical bisacurone treatment accelerated burn wound healing in rats, with marked reductions in inflammatory cell infiltration and tissue edema, indicating effective control of local inflammatory responses [20]. In a myocardial ischemia–reperfusion injury model, bisacurone was shown to ameliorate tissue damage by limiting inflammation and apoptosis, further underscoring its capacity to protect tissues from inflammation-driven injury [21]. Although these models differ in pathology, a common feature across studies is the consistent reduction in leukocyte infiltration and inflammatory mediator burden, aligning closely with the histopathological improvements observed in the present work.

Integration of molecular docking results provides a mechanistic framework for interpreting these in vivo effects. Docking analyses revealed favorable predicted binding of bisacurone to key inflammatory regulatory proteins involved in late-phase signaling, including enzymes associated with prostaglandin biosynthesis and regulators of nitric oxide and cytokine signaling pathways. Predicted interactions with prostaglandin-related enzymes are consistent with the pronounced inhibition observed during the prostaglandin-dependent phase of carrageenan inflammation [7], while engagement of nitric oxide– and MAPK-associated regulators provides a plausible explanation for reduced vascular permeability and inflammatory cell migration [6]. In addition, predicted modulation of NF-κB–associated regulatory proteins aligns with suppression of cytokine-driven inflammatory amplification [5,9]. Although docking does not establish direct biochemical inhibition, the concordance between predicted target engagement and observed physiological outcomes strengthens the biological plausibility of these mechanisms. Notably, bisacurone exhibits limited direct antioxidant activity, suggesting that its in vivo efficacy is more likely mediated through modulation of intracellular inflammatory signaling networks rather than nonspecific radical scavenging [17]. Collectively, these findings support bisacurone as a bioactive turmeric-derived compound that suppresses acute inflammation through coordinated regulation of late-phase inflammatory signaling processes.

The in silico results suggest that bisacurone is an electronically stable and moderately reactive sesquiterpenoid with a clear multi-target anti-inflammatory profile, mainly focused on IKKβ and COX, and supported by favorable pharmacokinetic and toxicity predictions. The DFT descriptors, including a HOMO–LUMO gap of 5.29 eV, moderate electrophilicity, balanced hardness and softness, and a dipole moment of 6.52 D, indicate selective and reversible non-covalent binding to protein pockets rather than nonspecific reactivity, which agrees with established conceptual DFT principles [22,23,24]. Docking across key inflammatory targets showed the strongest binding to IKKβ (−7.3 kcal/mol) and COX-1 (−7.2 kcal/mol), moderate affinity toward p38 MAPK and JNK1, and weaker but reasonable interactions with TLR4–MD-2, NF-κB p65, iNOS, and COX-2. This creates a clear target hierarchy where IKKβ and COX-1 act as primary targets, p38 and JNK1 act as supportive kinases, and the remaining proteins serve as secondary or context-dependent sites. This hierarchy matches in vivo studies showing that bisacurone reduces diabetic nephropathy and burn-related tissue injury by suppressing NF-κB activation, lowering iNOS and COX-2 expression, reducing TNF-α, IL-1β, and IL-6 levels, and activating Nrf2/HO-1 signaling [19,20]. It also aligns with reports on bisabolane-type sesquiterpenoids from *Curcuma longa* that regulate NF-κB and MAPK pathways and reduce NO, PGE_2_, and inflammatory cytokine release [25,26,27,28]. At the pathway level, the docking poses support a mechanism where stable binding at the ATP sites of IKKβ and MAPKs, along with occupation of the COX-1 active site and a functional region of iNOS, suppresses IκBα phosphorylation and MAPK signaling. This effect indirectly reduces NF-κB p65 activity and limits nitric oxide and prostaglandin production. The weaker binding to COX-2 and TLR4–MD-2 suggests that these proteins are not major direct targets, despite their regulation in vivo. ADME and toxicity predictions further support this profile. Bisacurone meets Lipinski, Veber, Ghose, Egan, and Muegge criteria without violations. It shows high intestinal absorption, blood–brain barrier permeability, no inhibition of major CYP enzymes, and no interaction with P-glycoprotein. ProTox-III classifies it as acute toxicity class 4, with no alerts for liver, kidney, heart, or genetic toxicity, which agrees with the known safety of Curcuma sesquiterpenoids at therapeutic levels [26,27,29,30,31,32]. Coarse-grained MD and normal mode analyses show that the IKKβ– and COX-1–bisacurone complexes retain stable cores with limited hinge flexibility, low eigenvalues, and correlated motions near the binding sites. This behavior supports adaptive binding without structural disruption [33,34]. A limitation of this study is that native-ligand docking scores could not be included for cross-comparison because such data were inconsistently reported across reference studies and were not generated within the present work. Although bisacurone has been shown to inhibit serum or cellular biomarkers of inflammation in previous studies [17,18,19,20,21], this study study offers in vivo functional validation of its anti-inflammatory effects, supported by histopathological analysis and in silico data. However, to further substantiate the proposed mechanisms and molecular targets, additional validation through molecular investigations at the protein and gene expression levels (such as Western blotting and RT-qPCR) will be necessary in future research.

## 4. Materials and Methods

### 4.1. Chemicals

This study used methanol (MeOH), ethyl acetate (EtOAc), and n-hexane, all acquired from Nacalai Tesque in Kyoto, Japan. Silica gel with a particle size of 63–200 μm was supplied by Kanto Chemical Co., Tokyo, Japan and Toyopearl HW-40F was purchased from Tosoh Corporation, Tokyo, Japan. Carrageenan came from Wako Pure Chemical Corporation, Osaka, Japan. All other reagents and solvents were of analytical grade.

### 4.2. Isolation and Structural Elucidation of Bisacurone

Ryudai gold variety of *Curcuma longa* was registered by the Ministry of Agriculture, Forestry, and Fisheries, Japan (Registration No. 21485, 29 February 2012). We have previously isolated, purified, and studied the biological activities of pure active compounds from this variety [16,35,36]. In our previous work, bioactivity-guided fractionation of *Curcuma longa* (Ryudai gold) led to the isolation of several compounds, including bisacurone [16,35]. While bisacurone was successfully isolated and structurally characterized, it exhibited comparatively weak antioxidant activity and was, therefore, not included in the antioxidant-focused analysis of that study [16]. Given emerging evidence that bisacurone exerts biological effects beyond direct radical scavenging, the compound was retained and subsequently evaluated for its anti-inflammatory activity in this study. The rhizomes used in this study were grown from the registered *Curcuma longa* Ryudai Gold cultivar. In brief, the rhizomes of RD were harvested when all the shoots withered completely. The rhizomes were washed, sliced, and dried in a hot-air oven at 50 °C for 72 h. We got 10% dry powder from fresh rhizomes of RD. The turmeric powder was macerated with MeOH for 2 days at room temperature (25 °C) with a magnetic stirring to prevent oxidation by air and shielding from sunlight. Then the solutions were filtered through double filter paper (Whatman™ No. 1). The filtered solutions were evaporated under reduced pressure at 40 °C. The yield of extracts was recorded and kept in the refrigerator at 4 °C for experimental analyses. The MeOH extract of RD (300 g) was partitioned with water, *n*-hexane, and EtOAc. The EtOAc fraction was subjected to chromatography on a silica gel (75 g) column (30 × 3 cm). Elution was carried out using *n*-hexane and EtOAc with increasing amounts of EtOAc [*n*-hexane:EtOAc; 100:0 (F1), 80:20 (F2), 60:40 (F3), 40:60 (F4), 20:80 (F5), and 0:100 (F6)]. Among the six fractions of EtOAc part of RD, F6 part of RD was passed through Sep-Pak C18 and obtained two fractions by using 60% (F6-1) and 80% (F6-2) MeOH. 60% (F6-1) MeOH fraction was divided into 6 fractions (F6-1.1 to F6-1.6) using Toyopearl HW-40F column (50 × 1.5 cm) chromatography, eluted with 40%, 50%, 60%, 70%, and 90% MeOH. Among these 6 fractions, fractions (F6-1.1) were purified by C18 reversed-phase HPLC (Inter Sustain C18 column) equipped with water and MeOH as the mobile phase with a flow rate of 4.0 mL/min and detected at 280 nm. The isolated peaks were dissolved in MeOH-*d_4_* and then subjected to spectral analysis. Nuclear magnetic resonance (NMR) spectra were recorded on BRUKER NMR spectrometers, Rheinstetten, Germany (500 MHz for ^1^H and 125 MHz for ^13^C) at room temperature. Mass Spectrometry (MS) experiments were carried out on a Waters Mass Spectrometer (Quattro Micro, Milford, MA, USA) using an electrospray ionization (ESI) probe under the following instrumental conditions: Column: COSMOSIL 5C18 AR-II, 2 × 150 mm. Solvent A: water (0.1% formic acid), Solvent B: acetonitrile, Flow rate: 0.2 mL/min, injection volume: 5 μL, run time: 30 min, pump mode: binary gradient, time program: 10% B (0 min) − 100% B (20 min) − 100% B (25 min) − 10% B (25.1 min) − 10% B (30 min), MS ionization mode: ES (+), capillary voltage: 4.0 kV, cone voltage: 20 V, source temp.: 120 °C, desolvation temp.: 350 °C, cone gas flow: 100 L/h, desolvation gas flow: 800 L/h.

The chemical structure of the isolated compound was established using ^1^H and ^13^C NMR spectroscopy. Bisacurone was obtained as a pale yellow oil. UV λ_max_ nm: 244. ESI-MS (+): *m*/*z* 235.2 [M-H_2_O+H]^+^, 275.2 [M+Na]^+^. ^1^H-NMR (CD_3_OD) δ: 6.19 (1H, s, H-10), 5.61 (1H, dd, *J* = 10, 2 Hz, H-2), 5.54 (1H, d, *J* = 10 Hz, H-3), 3.69 (1H, m, H-5), 2.53 (1H, dd, *J* = 15, 5 Hz, H-8a), 2.27 (1H, m, H-1), 2.24 (1H, dd, *J* = 15, 9 Hz, H-8b), 2.11 (3H, s, H-13), 2.10 (1H, m, H-7), 1.90 (3H, s, H-12), 1.81 (1H, m, H-6a), 1.67 (1H, m, H-6b), 1.22 (3H, s, H-15), 0.89 (3H, d, *J* = 7 Hz, H-14). ^13^C-NMR (CD_3_OD) δ: 203.6 (C-9), 157.3 (C-11). 133.5 (C-3), 132.5 (C-2), 125.0 (C-10), 74.0 (C-5), 70.8 (C-4), 49.7 (C-8), 37.7 (C-1), 34.6 (C-7), 29.3 (C-6), 27.7 (C-12), 24.8 (C-15), 20.9 (C-13), 17.2 (C-14). Based on these spectral features and comparison with literature reports, the compound was identified as bisacurone [37] (Figure 1). After repeated fractionation and purification reported in our previous report [16], 39.6 mg (1.32%) pure bisacurone was yielded from 46 g MeOH extract.

### 4.3. Experimental Animal

Experimental rats were supplied by the International Centre for Diarrhoeal Disease Research, Bangladesh (icddr,b), Dhaka. Before starting the experiments, the rats were allowed to adapt to the laboratory environment for one week. They were housed in group-specific rectangular plastic cages with compartments. The animal room was kept under controlled conditions, with good ventilation, a temperature of 28 ± 2 °C, relative humidity of 70–80%, and natural light cycles. Rats had free access to standard pellet feed, formulated according to National Research Council guidelines, and water throughout the study. All experimental protocols followed the regulations approved by the Bangladesh Agricultural University Animal Welfare and Ethics Committee (AWEEC/BAU/2020(2)).

### 4.4. Anti-Inflammatory Study

To study the anti-inflammatory effect of bisacurone, we used a paw edema model caused by carrageenan, as previously described by Islam et al. (2024) [38]. Male Wistar rats (*n* = 5) were given bisacurone at 100 µg/kg body weight by oral gavage. This dose was selected according to previous studies [19,21]. The compound was mixed in corn oil, and each rat received a total of 1 mL of the solution. Rats in the control group were given the same amount of corn oil without bisacurone. After thirty minutes, inflammation was triggered by injecting 50 µL of 1% carrageenan solution (in saline) into one hind paw. The other paw received the same amount of saline to serve as a control. Paw sizes were recorded at 1, 3, and 6 h after injection using a digital caliper. The swelling was expressed as a percentage using the formula:

Edema % = (V_d_ − V_c_)/V_c_ × 100%

where V_d_ represents the volume of the inflamed paw and V_c_ represents the volume of the control paw.

Indomethacin (10 mg/kg body weight) was used as a standard reference drug to validate the anti-inflammatory model to compare the efficacy of bisacurone.

### 4.5. Histopathological Examination of the Paw Tissues

At 3 h after carrageenan injection, one rat from each group was euthanized and paw tissues were harvested. The tissues were fixed in 10% formalin, dehydrated through a graded series of ethanol, cleared in xylene, and embedded in paraffin wax. Paraffin-embedded tissues were sectioned at a thickness of 5 µm and stained with hematoxylin and eosin for histopathological examination.

### 4.6. Statistical Analysis

Data are presented as mean ± standard deviation (SD). Group differences were analyzed using one-way analysis of variance (ANOVA), followed by Duncan’s multiple range post hoc test. Statistical significance was defined as *p* < 0.05. Statistical analyses were not applied to histological assessments, which were considered nonparametric.

### 4.7. In Silico Analysis

#### 4.7.1. Preparation of Ligand and Conceptual Density Functional Theory (DFT) Calculations

The three-dimensional structure of the ligand bisacurone (PubChem CID: 14287397) was obtained from the PubChem database (https://pubchem.ncbi.nlm.nih.gov (accessed on 16 December 2025)) in .sdf format. Density Functional Theory (DFT) calculations were performed using the B3LYP functional and the 6-311+G(2d,p) basis set. Water was included as a solvent using the SMD solvation model [39]. Geometry optimization and frequency analysis were conducted to confirm that the structure corresponded to a true energy minimum (Figure 1D).

DFT is a useful method for studying molecular properties by evaluating molecular orbital energies. It provides insight into how the chemical structure of a molecule relates to its activity. This approach is based on the principles of Hohenberg and Kohn, which describe how electron density determines molecular behavior [40]. Conceptual DFT, a branch of DFT that focuses on electron density, was also used to better understand the chemical characteristics of bisacurone in a straightforward manner [41].

The optimized bisacurone structure was analyzed to determine its molecular orbital energies. From the electron density, several molecular descriptors were calculated, such as the electrophilicity index, chemical potential, molecular dipole moment, and electronegativity. All calculations were carried out using Gaussian 09 (Revision D.01) and visualized with GaussView 6.0.16 [42,43]. After optimization, the structure was converted to PDB format, making it suitable for molecular docking and interaction studies (Figure 1D).

#### 4.7.2. Preparation of Macromolecule

The 3D structures of the targets IKKβ (PDB ID: 4KIK), TLR4–MD-2 (3FXI), NF-κB p65 (3GUT), p38 MAPK (1A9U), JNK1 (3PZE), iNOS (3E7G), COX-1 (3KK6) and COX-2 (5IKV) were employed for the interaction study specifically downloaded from the RCSB Protein Data Bank (http://www.rcsb.org/pdb/home/home.do (accessed on 16 December 2025))in legacy PDB format [44,45,46,47,48,49,50,51]. Using BIOVIA Discovery Studio 2025 and PyMol 2.3.3, removal of heteroatoms, ligands and water molecules were done. Addition of polar hydrogen and energy minimization were done by Swiss PDB viewer 4.1.0 [52]. Energy was minimized by GROMOS 43B1 force field. The protein was saved in pdbqt format for docking.

#### 4.7.3. Molecular Docking

Energy minimization was performed using Open Babel with the UFF force field. After that, the molecular docking simulations were performed using the Autodock Vina 1.1.2 within PyRx 0.8 [53]. Bisacurone was docked to all prepared macromolecules. The exhaustiveness was set to 8 for all docking experiments. The grid boxes were fitted in the active site of the receptors. For IKKβ, the grid box was 28.1861 × 23.1842 × 23.5532 Å, centered at x = −16.7769, y = −30.8336, z = −74.9540. For TLR4–MD-2, the grid box was 81.4799 × 89.4334 × 85.2511 Å, centered at x = 14.4311, y = 10.2900, z = 23.6132. For NF-κB p65, the grid box was 25.0000 × 25.0000 × 25.0000 Å, centered at x = 46.4492, y = −21.4125, z = 48.7787. For p38 MAPK, the grid box was 20.8933 × 25.0000 × 18.9943 Å, centered at x = 5.1130, y = 12.7427, z = 28.4632. For JNK1, the grid box was 13.8988 × 16.3175 × 34.5323 Å, centered at x = 25.3914, y = 15.0176, z = 21.4669. For iNOS, the grid box was 17.7511 × 15.0058 × 16.5660 Å, centered at x = 70.4776, y = 6.9462, z = 80.5889. For COX-1, the grid box was 20.4396 × 24.6198 × 22.7854 Å, centered x = −32.292, y = 44.4581, z = −6.6831. For COX-2, the grid box was 13.9188 × 12.9941 × 13.4236 Å, centered x = 143.7035, y = 191.741, z = 204.6127. Best outputs were captured, and 3D and 2D figures were generated by BIOVIA Discovery Studio 2025 visualizer.

#### 4.7.4. In Silico Drug-Likeness and Toxicity Predictions

The drug-likeness of bisacurone was predicted using standard guidelines [31]. The SMILES structures of bisacurone were collected from the PubChem database and added to the Swiss ADME tool to estimate basic pharmacokinetic features. These predictions followed Lipinski’s Rule of Five and Veber’s Rule [30,31,32]. The study also predicted organ toxicity and other toxicological effects using the ProTox-III server [29].

#### 4.7.5. Molecular Dynamics Simulation Protocol

Molecular dynamics (MD) simulations of the protein–ligand complexes were performed using CABS-flex version 2.0 (https://biocomp.chem.uw.edu.pl/CABSflex2 (accessed on 16 December 2025)) and the iMOD server (https://imods.iqf.csic.es/ (accessed on 16 December 2025)) [33,34]. CABS-flex was used to evaluate protein flexibility by measuring root-mean-square fluctuations (RMSF). The simulation length was set to 10 ns, while all other settings remained at their default values. RMSF data were obtained from the MD trajectories or NMR ensembles using the standard analysis options provided by the server. The 10 ns molecular dynamics (MD) simulations in this study were designed as qualitative analyses, aimed at generating hypotheses rather than conducting production-level free-energy calculations.

To examine the stability and molecular motion of the docked protein–TMZ complexes, additional MD simulations were carried out with the iMOD server. iMODS was applied to study the structural dynamics of the complexes and to describe their collective molecular motions. The stability of each protein–TMZ complex was evaluated using parameters such as deformability, B-factor, eigenvalues, variance, covariance maps, and elastic network models [54]. Docked PDB structures were used as input files and uploaded directly to the iMODS server, with all simulation parameters kept at default settings.

## 5. Conclusions

This study provides integrated in vivo and in silico evidence that bisacurone, a sesquiterpenoid from the Ryudai gold variety of *Curcuma longa*, exhibits significant anti-inflammatory activity. Bisacurone effectively attenuated carrageenan-induced paw edema and preserved tissue architecture by reducing dermal thickening and inflammatory cell infiltration, confirming suppression of acute inflammatory progression at both functional and histopathological levels. Computational analyses support these findings by identifying stable and energetically favorable interactions with key inflammatory regulators, particularly IKKβ and COX-1, alongside additional engagement of MAPKs and iNOS, consistent with a multi-target mode of action. Favorable predicted pharmacokinetic and toxicity profiles further support its biological relevance. Collectively, these results suggest that bisacurone mediates anti-inflammatory effects primarily through modulation of inflammatory signaling pathways rather than direct antioxidant activity. Our results concluded that bisacurone holds promise both as a functional food-derived anti-inflammatory supplement and as a lead compound for further drug development. The in vivo efficacy of bisacurone was comparable to that of indomethacin, supporting its potential as a naturally derived bioactive anti-inflammatory candidate with the multi-target mechanistic activity. Further studies on chronic efficacy, bioavailability, and safety are warranted to support bisacurone’s development as a functional food-based anti-inflammatory agent.

## Figures and Tables

**Table 1:** Statistical insights of DFT-based molecular descriptors of bisacurone.

Total Energy (E_ γ_) (eV)	Molecular Dipole Moment (Debye)	E_HOMO_ (eV)	E_LUMO_ (eV)	E_gap_ (eV)	Absolute Hardness (η)	Global Softness (σ) (eV^−1^)	Electronegativity (χ)	Chemical Potential (μ)	Electrophilicity Index (ω) (eV)
−22,093.6	6.518061	−6.754	−1.464	5.29	2.645 eV	0.378	4.109 eV	−4.109	3.19

**Table 2:** Molecular docking results of bisacurone with receptors.

Receptors	Binding Energy (kcal/mol)	Information on Hydrogen Bond			Information on Other Interactions		
		Number of Hydrogen Bonds	Amino Acids Involved in H-Bonding	Hydrogen Bond Distance	Number of Bonds	Amino Acids Involved	Bond Type
IKKβ (4KIK)	−7.3	2	ASN28, ASN28	2.64462 Å 2.39063 Å	10	ILE165, VAL29, LYS44, MET96, LEU21, VAL152, CYS99, VAL152, TYR98	Hydrophobic
TLR4–MD-2 (3FXI)	−5.4	3	GLU111, SER184, ARG106	2.63259 Å 2.03719 Å 2.510782 Å	4	ARG106, ALA107, LEU212, HIS159	Hydrophobic
NF-κB p65 (RelA) (3GUT)	−5.4	1	ARG605	2.42814 Å	11	VAL248, ARG605, ARG246, LYS572, LYS218, PHE607	Hydrophobic
p38 MAPK (1A9U)	−6.2	1	LYS53	1.94967 Å	10	ALA51, VAL30, ILE84, ILE85, ILE86, ILE87, ILE88, ILE89, ILE90, ILE91	Hydrophobic
JNK1 (3PZE)	−6.7	0	-	-	11	VAL40, ALA53, LEU168, ILE32, ILE86, MET111, VAL158, LEU110	Hydrophobic
iNOS (3E7G)	−5.6	3	ASN482, ASN482, GLY117	2.67859 Å 1.94651 Å 3.35344 Å	6	CYS110, LEU108, PRO122, LEU125	Hydrophobic
COX-1 (3KK6)	−7.2	2	ARG120, ILE523	2.2323 Å 2.76692 Å	16	VAL116, VAL349, LEU352, LEU359, ILE523, LEU531, ALA527, TYR355, TRP387, PHE518.	Hydrophobic
COX-2 (5F19)	−4.1	1	ARG216	2.70304 Å	7	ARG216, ALA219, PRO218, PHE220	Hydrophobic

**Table 3:** Drug-likeness predictions of bisacurone by Swiss ADME.

Mol. Wt. (g/mol)	NHD	NHA	NRB	Lipophilicity		Log S (ESOL) Water Solubility	LV	VV	GV	EV	MV
				Log P (iLOGP)	Log P (MLOGP)						
252.35	2	3	4	2.74	1.88	−2.32	0	0	0	0	0

Key: NHD = number of hydrogen donors, NHA = number of hydrogen acceptors, NRB = number of rotatable bonds, LV = Lipinski’s Violations, VV = Veber’s Violations, GV = Ghose’sViolations, EV = Egan’s Violations, and MV = Muegge’s Vioations.

**Table 4:** ADME predictions of bisacurone by Swiss ADME.

(Log Kp) cm/s	GIA	BBB	Inhibitor Interaction					
			P-gp	CYP1A2 Inhibitor	CYP2C19 Inhibitor	CYP2C9 Inhibitor	CYP2D6 Inhibitor	CYP3A4 Inhibitor
−6.5	High	Yes	No	No	No	No	No	No

Key: BBB = blood–brain barrier permeability; GIA = gastrointestinal absorption; Log Kp = skin permeation value; CYP = cytochrome-P; P-gp = P-glycoprotein substrate.

**Table 5:** Toxicity prediction of bisacurone by ProTox-III.

LD50 (mg/kg)	Toxicity Class			Organ Toxicity					
		Hepato	Carcino	Immuno	Mutagen	Cytoto	Nephro	Respi	Cardio
1170	4	Inactive	Inactive	Inactive	Inactive	Inactive	Inactive	Inactive	Inactive

Key: Hepato = hepatotoxicity; Carcino = carcinogenicity; Immuno = immunotoxicity; Mutagen = mutagenicity; Cytoto = cytotoxicity; Cardio = cardiotoxicity; Nephro = nephrotoxicity; Respi = respiratory toxicity; LD = lethal dose.

## Data Availability

The data that support the findings of this study are available from the corresponding authors.

## Supplementary Materials

The following supporting information can be downloaded at: https://www.mdpi.com/article/10.3390/molecules31030548/s1, The computational and electronic properties of bisacurone were evaluated using Density Functional Theory (DFT). Geometry optimization details, molecular orbital, dipole moment, and frequency analyses are provided in Supplementary Materials. Structural stability assessments for COX-1 and IKKβ complexes are presented in Supplementary Tables S1 and S2.
