# Supplementary material for: Anti-Inflammatory Effects of Bisacurone Isolated from Curcuma longa (Ryudai Gold): An In Vivo and In Silico Study
Source: Molecules. 2026 Feb 4;31(3):548. doi: 10.3390/molecules31030548 (PMC12899999; doi:10.3390/molecules31030548)
Supplement: Supplementary file 1 [file molecules-31-00548-s001.zip › molecules-4095030-supplementary.pdf]

S-1: Supplementary LOG File of Optimization of Bisacurone using Density Functional Theory.

Entering Link 1 = C:\G09W\l1.exe PID= 16808.

Copyright (c) 1988,1990,1992,1993,1995,1998,2003,2009,2013,

Gaussian, Inc. All Rights Reserved.

This is part of the Gaussian(R) 09 program. It is based on

the Gaussian(R) 03 system (copyright 2003, Gaussian, Inc.),

the Gaussian(R) 98 system (copyright 1998, Gaussian, Inc.),

the Gaussian(R) 94 system (copyright 1995, Gaussian, Inc.),

the Gaussian 92(TM) system (copyright 1992, Gaussian, Inc.),

the Gaussian 90(TM) system (copyright 1990, Gaussian, Inc.),

the Gaussian 88(TM) system (copyright 1988, Gaussian, Inc.),

the Gaussian 86(TM) system (copyright 1986, Carnegie Mellon

University), and the Gaussian 82(TM) system (copyright 1983,

Carnegie Mellon University). Gaussian is a federally registered

trademark of Gaussian, Inc.

This software contains proprietary and confidential information,

including trade secrets, belonging to Gaussian, Inc.

This software is provided under written license and may be

used, copied, transmitted, or stored only in accord with that

written license.

The following legend is applicable only to US Government

contracts under FAR:

RESTRICTED RIGHTS LEGEND

Use, reproduction and disclosure by the US Government is

subject to restrictions as set forth in subparagraphs (a)

and (c) of the Commercial Computer Software - Restricted

Rights clause in FAR 52.227-19.

Gaussian, Inc.

340 Quinnipiac St., Bldg. 40, Wallingford CT 06492

Warning -- This program may not be used in any manner that competes with the business of Gaussian, Inc. or will provide assistance to any competitor of Gaussian, Inc. The licensee of this program is prohibited from giving any competitor of Gaussian, Inc. access to this program. By using this program, the user acknowledges that Gaussian, Inc. is engaged in the business of creating and licensing software in the field of computational chemistry and represents and warrants to the licensee that it is not a competitor of Gaussian, Inc. and that it will not use this program in any manner prohibited above.

Cite this work as:

Gaussian 09, Revision D.01,

M. J. Frisch, G. W. Trucks, H. B. Schlegel, G. E. Scuseria,

M. A. Robb, J. R. Cheeseman, G. Scalmani, V. Barone, B. Mennucci,

G. A. Petersson, H. Nakatsuji, M. Caricato, X. Li, H. P. Hratchian,

A. F. Izmaylov, J. Bloino, G. Zheng, J. L. Sonnenberg, M. Hada,

M. Ehara, K. Toyota, R. Fukuda, J. Hasegawa, M. Ishida, T. Nakajima,

Y. Honda, O. Kitao, H. Nakai, T. Vreven, J. A. Montgomery, Jr.,

J. E. Peralta, F. Ogliaro, M. Bearpark, J. J. Heyd, E. Brothers,

K. N. Kudin, V. N. Staroverov, T. Keith, R. Kobayashi, J. Normand,

K. Raghavachari, A. Rendell, J. C. Burant, S. S. Iyengar, J. Tomasi,

M. Cossi, N. Rega, J. M. Millam, M. Klene, J. E. Knox, J. B. Cross,

V. Bakken, C. Adamo, J. Jaramillo, R. Gomperts, R. E. Stratmann,

O. Yazyev, A. J. Austin, R. Cammi, C. Pomelli, J. W. Ochterski,

R. L. Martin, K. Morokuma, V. G. Zakrzewski, G. A. Voth,

P. Salvador, J. J. Dannenberg, S. Dapprich, A. D. Daniels,

O. Farkas, J. B. Foresman, J. V. Ortiz, J. Cioslowski,

and D. J. Fox, Gaussian, Inc., Wallingford CT, 2013.

****

Gaussian 09: IA32W-G09RevD.01 24-Apr-2013

19-Dec-2025

****

%chk=E:\Project Docking Bisacurone\working files\Bisacurone_op.chk

----

opt freq b3lyp/6-31+g(2d,p) scrf=(smd,solvent=water) geom=connectivi

ty

1/14=-1,18=20,19=15,26=3,38=1,57=2/1,3;

2/9=110,12=2,17=6,18=5,40=1/2;

3/5=1,6=6,7=112,11=2,16=1,25=1,30=1,70=32201,71=1,72=1,74=-5/1,2,3;

4//1;

5/5=2,38=5,53=1/2;

6/7=2,8=2,9=2,10=2,28=1/1;

7//1,2,3,16;

1/14=-1,18=20,19=15,26=3/3(2);

2/9=110/2;

99//99;

2/9=110/2;

3/5=1,6=6,7=112,11=2,16=1,25=1,30=1,70=32205,71=1,72=1,74=-5/1,2,3;

4/5=5,16=3,69=1/1;

5/5=2,38=5,53=1/2;

7//1,2,3,16;

1/14=-1,18=20,19=15,26=3/3(-5);

2/9=110/2;

6/7=2,8=2,9=2,10=2,19=2,28=1/1;

99/9=1/99;

OP+FQ

Symbolic Z-matrix:

Charge = 0 Multiplicity = 1

O	1.2533	2.2138	-0.3192
O	3.8828	0.2164	1.27
O	-2.3795	-1.0169	-1.4099
C	0.6576	-0.7273	-0.4434
C	0.8391	0.1282	0.8257
C	1.818	1.2879	0.6076
C	-0.0393	-2.0427	-0.0909
C	3.2036	0.7794	0.1405
C	1.9625	-0.9862	-1.1503
C	3.0986	-0.3283	-0.8781
C	-1.3685	-1.7944	0.6382
C	0.8815	-2.9023	0.7768
C	4.0725	1.919	-0.4031
C	-2.3517	-0.977	-0.183
C	-3.3342	-0.1714	0.5972
C	-3.8617	0.9991	0.1976
C	-4.8526	1.828	0.9663
C	-3.4821	1.5745	-1.1419
H	0.0386	-0.1641	-1.1528
H	-0.1293	0.5566	1.1118
H	1.1927	-0.4515	1.6819
H	1.9357	1.8227	1.5587
H	-0.2438	-2.5953	-1.0183
H	1.9762	-1.743	-1.9308
H	3.9987	-0.6057	-1.4215
H	-1.2124	-1.323	1.6144
H	-1.8555	-2.7585	0.8351

H	1.8528	-3.0958	0.3125
H	0.4168	-3.8846	0.9283
H	1.0496	-2.4928	1.7763
H	4.1664	2.7189	0.3405
H	5.0923	1.572	-0.6091
H	3.6655	2.3474	-1.3251
H	0.401	2.5108	0.0425
H	3.3881	-0.5623	1.5764
H	-3.5867	-0.5635	1.5786
H	-5.1223	1.3502	1.9139
H	-4.4328	2.8128	1.1954
H	-5.7707	1.9621	0.3854
H	-4.0954	1.1376	-1.9363
H	-3.6496	2.6579	-1.1542
H	-2.4212	1.4412	-1.3752

GradGradGradGradGradGradGradGradGradGradGradGradGradGradGradGradGradGrad

Berny optimization.

Initialization pass.

! Initial Parameters !

! (Angstroms and Degrees) !

-------------------------- --------------------------

! Name Definition Value Derivative Info. !

! R1 R(1,6) 1.4266 estimate D2E/DX2 !

! R2 R(1,34) 0.9723 estimate D2E/DX2 !

! R3 R(2,8) 1.4332 estimate D2E/DX2 !

! R4 R(2,35) 0.9721 estimate D2E/DX2 !

! R5 R(3,14) 1.2279 estimate D2E/DX2 !

! R6 R(4,5) 1.5412 estimate D2E/DX2 !

! R7 R(4,7) 1.5298 estimate D2E/DX2 !

!	R8	R(4,9)	1.5065	estimate D2E/DX2	!
!	R9	R(4,19)	1.0971	estimate D2E/DX2	!
!	R10	R(5,6)	1.5332	estimate D2E/DX2	!
!	R11	R(5,20)	1.0969	estimate D2E/DX2	!
!	R12	R(5,21)	1.0928	estimate D2E/DX2	!
!	R13	R(6,8)	1.5481	estimate D2E/DX2	!
!	R14	R(6,22)	1.0975	estimate D2E/DX2	!
!	R15	R(7,11)	1.5362	estimate D2E/DX2	!
!	R16	R(7,12)	1.5296	estimate D2E/DX2	!
!	R17	R(7,23)	1.0988	estimate D2E/DX2	!
!	R18	R(8,10)	1.5085	estimate D2E/DX2	!
!	R19	R(8,13)	1.5327	estimate D2E/DX2	!
!	R20	R(9,10)	1.3408	estimate D2E/DX2	!
!	R21	R(9,24)	1.0873	estimate D2E/DX2	!
!	R22	R(10,25)	1.0874	estimate D2E/DX2	!
!	R23	R(11,14)	1.5196	estimate D2E/DX2	!
!	R24	R(11,26)	1.0952	estimate D2E/DX2	!
!	R25	R(11,27)	1.0979	estimate D2E/DX2	!
!	R26	R(12,28)	1.0938	estimate D2E/DX2	!
!	R27	R(12,29)	1.0972	estimate D2E/DX2	!
!	R28	R(12,30)	1.0931	estimate D2E/DX2	!
!	R29	R(13,31)	1.0962	estimate D2E/DX2	!
!	R30	R(13,32)	1.0967	estimate D2E/DX2	!
!	R31	R(13,33)	1.0951	estimate D2E/DX2	!
!	R32	R(14,15)	1.491	estimate D2E/DX2	!
!	R33	R(15,16)	1.3446	estimate D2E/DX2	!
!	R34	R(15,36)	1.0866	estimate D2E/DX2	!
!	R35	R(16,17)	1.5033	estimate D2E/DX2	!
!	R36	R(16,18)	1.5065	estimate D2E/DX2	!
!	R37	R(17,37)	1.095	estimate D2E/DX2	!
!	R38	R(17,38)	1.0948	estimate D2E/DX2	!
!	R39	R(17,39)	1.0947	estimate D2E/DX2	!
!	R40	R(18,40)	1.0946	estimate D2E/DX2	!

! R41	R(18,41)	1.0963	estimate D2E/DX2	!
! R42	R(18,42)	1.0944	estimate D2E/DX2	!
! A1	A(6,1,34)	107.671	estimate D2E/DX2	!
! A2	A(8,2,35)	108.7784	estimate D2E/DX2	!
! A3	A(5,4,7)	109.9497	estimate D2E/DX2	!
! A4	A(5,4,9)	112.3195	estimate D2E/DX2	!
! A5	A(5,4,19)	108.2966	estimate D2E/DX2	!
! A6	A(7,4,9)	110.7904	estimate D2E/DX2	!
! A7	A(7,4,19)	109.4743	estimate D2E/DX2	!
! A8	A(9,4,19)	105.8742	estimate D2E/DX2	!
! A9	A(4,5,6)	112.2039	estimate D2E/DX2	!
! A10	A(4,5,20)	109.1227	estimate D2E/DX2	!
! A11	A(4,5,21)	112.8805	estimate D2E/DX2	!
! A12	A(6,5,20)	107.78	estimate D2E/DX2	!
! A13	A(6,5,21)	107.8251	estimate D2E/DX2	!
! A14	A(20,5,21)	106.768	estimate D2E/DX2	!
! A15	A(1,6,5)	109.3056	estimate D2E/DX2	!
! A16	A(1,6,8)	111.8054	estimate D2E/DX2	!
! A17	A(1,6,22)	106.8097	estimate D2E/DX2	!
! A18	A(5,6,8)	111.4641	estimate D2E/DX2	!
! A19	A(5,6,22)	108.2874	estimate D2E/DX2	!
! A20	A(8,6,22)	108.9991	estimate D2E/DX2	!
! A21	A(4,7,11)	111.3796	estimate D2E/DX2	!
! A22	A(4,7,12)	109.8585	estimate D2E/DX2	!
! A23	A(4,7,23)	108.8295	estimate D2E/DX2	!
! A24	A(11,7,12)	110.0269	estimate D2E/DX2	!
! A25	A(11,7,23)	108.7136	estimate D2E/DX2	!
! A26	A(12,7,23)	107.9513	estimate D2E/DX2	!
! A27	A(2,8,6)	108.3851	estimate D2E/DX2	!
! A28	A(2,8,10)	106.0625	estimate D2E/DX2	!
! A29	A(2,8,13)	107.6336	estimate D2E/DX2	!
! A30	A(6,8,10)	112.4967	estimate D2E/DX2	!
! A31	A(6,8,13)	111.7272	estimate D2E/DX2	!

! A32 A(10,8,13) 110.2396 estimate D2E/DX2 !

! A33 A(4,9,10) 123.6676 estimate D2E/DX2 !

! A34 A(4,9,24) 117.8649 estimate D2E/DX2 !

! A35 A(10,9,24) 118.4647 estimate D2E/DX2 !

! A36 A(8,10,9) 123.8061 estimate D2E/DX2 !

! A37 A(8,10,25) 117.8492 estimate D2E/DX2 !

! A38 A(9,10,25) 118.3447 estimate D2E/DX2 !

! A39 A(7,11,14) 112.9719 estimate D2E/DX2 !

! A40 A(7,11,26) 111.6704 estimate D2E/DX2 !

! A41 A(7,11,27) 109.0852 estimate D2E/DX2 !

! A42 A(14,11,26) 110.0211 estimate D2E/DX2 !

! A43 A(14,11,27) 106.3954 estimate D2E/DX2 !

! A44 A(26,11,27) 106.339 estimate D2E/DX2 !

! A45 A(7,12,28) 113.1525 estimate D2E/DX2 !

! A46 A(7,12,29) 109.0563 estimate D2E/DX2 !

! A47 A(7,12,30) 113.6238 estimate D2E/DX2 !

! A48 A(28,12,29) 106.0413 estimate D2E/DX2 !

! A49 A(28,12,30) 108.5319 estimate D2E/DX2 !

! A50 A(29,12,30) 105.9181 estimate D2E/DX2 !

! A51 A(8,13,31) 110.52 estimate D2E/DX2 !

! A52 A(8,13,32) 111.0087 estimate D2E/DX2 !

! A53 A(8,13,33) 112.2577 estimate D2E/DX2 !

! A54 A(31,13,32) 106.1791 estimate D2E/DX2 !

! A55 A(31,13,33) 108.512 estimate D2E/DX2 !

! A56 A(32,13,33) 108.1323 estimate D2E/DX2 !

! A57 A(3,14,11) 122.4898 estimate D2E/DX2 !

! A58 A(3,14,15) 121.7025 estimate D2E/DX2 !

! A59 A(11,14,15) 115.7352 estimate D2E/DX2 !

! A60 A(14,15,16) 124.9843 estimate D2E/DX2 !

! A61 A(14,15,36) 115.5173 estimate D2E/DX2 !

! A62 A(16,15,36) 119.4316 estimate D2E/DX2 !

! A63 A(15,16,17) 125.9173 estimate D2E/DX2 !

! A64 A(15,16,18) 119.8602 estimate D2E/DX2 !

! A65	A(17,16,18)	114.215	estimate D2E/DX2	!
! A66	A(16,17,37)	111.3629	estimate D2E/DX2	!
! A67	A(16,17,38)	110.5026	estimate D2E/DX2	!
! A68	A(16,17,39)	110.4277	estimate D2E/DX2	!
! A69	A(37,17,38)	107.8103	estimate D2E/DX2	!
! A70	A(37,17,39)	107.825	estimate D2E/DX2	!
! A71	A(38,17,39)	108.8113	estimate D2E/DX2	!
! A72	A(16,18,40)	110.5901	estimate D2E/DX2	!
! A73	A(16,18,41)	110.4214	estimate D2E/DX2	!
! A74	A(16,18,42)	112.7863	estimate D2E/DX2	!
! A75	A(40,18,41)	107.4992	estimate D2E/DX2	!
! A76	A(40,18,42)	109.8663	estimate D2E/DX2	!
! A77	A(41,18,42)	105.431	estimate D2E/DX2	!
! D1	D(34,1,6,5)	57.5969	estimate D2E/DX2	!
! D2	D(34,1,6,8)	-178.5029	estimate D2E/DX2	!
! D3	D(34,1,6,22)	-59.3489	estimate D2E/DX2	!
! D4	D(35,2,8,6)	64.3444	estimate D2E/DX2	!
! D5	D(35,2,8,10)	-56.6724	estimate D2E/DX2	!
! D6	D(35,2,8,13)	-174.6595	estimate D2E/DX2	!
! D7	D(7,4,5,6)	166.1797	estimate D2E/DX2	!
! D8	D(7,4,5,20)	-74.4398	estimate D2E/DX2	!
! D9	D(7,4,5,21)	44.0985	estimate D2E/DX2	!
! D10	D(9,4,5,6)	42.3172	estimate D2E/DX2	!
! D11	D(9,4,5,20)	161.6977	estimate D2E/DX2	!
! D12	D(9,4,5,21)	-79.764	estimate D2E/DX2	!
! D13	D(19,4,5,6)	-74.2448	estimate D2E/DX2	!
! D14	D(19,4,5,20)	45.1357	estimate D2E/DX2	!
! D15	D(19,4,5,21)	163.674	estimate D2E/DX2	!
! D16	D(5,4,7,11)	54.9241	estimate D2E/DX2	!
! D17	D(5,4,7,12)	-67.2448	estimate D2E/DX2	!
! D18	D(5,4,7,23)	174.7607	estimate D2E/DX2	!
! D19	D(9,4,7,11)	179.672	estimate D2E/DX2	!
! D20	D(9,4,7,12)	57.5031	estimate D2E/DX2	!

! D21	D(9,4,7,23)	-60.4914	estimate D2E/DX2	!
! D22	D(19,4,7,11)	-63.931	estimate D2E/DX2	!
! D23	D(19,4,7,12)	173.9001	estimate D2E/DX2	!
! D24	D(19,4,7,23)	55.9056	estimate D2E/DX2	!
! D25	D(5,4,9,10)	-13.1063	estimate D2E/DX2	!
! D26	D(5,4,9,24)	167.5185	estimate D2E/DX2	!
! D27	D(7,4,9,10)	-136.4995	estimate D2E/DX2	!
! D28	D(7,4,9,24)	44.1253	estimate D2E/DX2	!
! D29	D(19,4,9,10)	104.8994	estimate D2E/DX2	!
! D30	D(19,4,9,24)	-74.4759	estimate D2E/DX2	!
! D31	D(4,5,6,1)	66.6556	estimate D2E/DX2	!
! D32	D(4,5,6,8)	-57.4454	estimate D2E/DX2	!
! D33	D(4,5,6,22)	-177.3368	estimate D2E/DX2	!
! D34	D(20,5,6,1)	-53.508	estimate D2E/DX2	!
! D35	D(20,5,6,8)	-177.6089	estimate D2E/DX2	!
! D36	D(20,5,6,22)	62.4996	estimate D2E/DX2	!
! D37	D(21,5,6,1)	-168.4285	estimate D2E/DX2	!
! D38	D(21,5,6,8)	67.4706	estimate D2E/DX2	!
! D39	D(21,5,6,22)	-52.4209	estimate D2E/DX2	!
! D40	D(1,6,8,2)	161.8854	estimate D2E/DX2	!
! D41	D(1,6,8,10)	-81.1633	estimate D2E/DX2	!
! D42	D(1,6,8,13)	43.4555	estimate D2E/DX2	!
! D43	D(5,6,8,2)	-75.436	estimate D2E/DX2	!
! D44	D(5,6,8,10)	41.5153	estimate D2E/DX2	!
! D45	D(5,6,8,13)	166.1341	estimate D2E/DX2	!
! D46	D(22,6,8,2)	44.0342	estimate D2E/DX2	!
! D47	D(22,6,8,10)	160.9854	estimate D2E/DX2	!
! D48	D(22,6,8,13)	-74.3957	estimate D2E/DX2	!
! D49	D(4,7,11,14)	59.5435	estimate D2E/DX2	!
! D50	D(4,7,11,26)	-65.1072	estimate D2E/DX2	!
! D51	D(4,7,11,27)	177.6475	estimate D2E/DX2	!
! D52	D(12,7,11,14)	-178.385	estimate D2E/DX2	!
! D53	D(12,7,11,26)	56.9644	estimate D2E/DX2	!

! D54   D(12,7,11,27)   -60.2809   estimate D2E/DX2   !

! D55   D(23,7,11,14)   -60.3617   estimate D2E/DX2   !

! D56   D(23,7,11,26)   174.9877   estimate D2E/DX2   !

! D57   D(23,7,11,27)   57.7423   estimate D2E/DX2   !

! D58   D(4,7,12,28)   -55.9833   estimate D2E/DX2   !

! D59   D(4,7,12,29)   -173.7369   estimate D2E/DX2   !

! D60   D(4,7,12,30)   68.3721   estimate D2E/DX2   !

! D61   D(11,7,12,28)   -178.9526   estimate D2E/DX2   !

! D62   D(11,7,12,29)   63.2938   estimate D2E/DX2   !

! D63   D(11,7,12,30)   -54.5972   estimate D2E/DX2   !

! D64   D(23,7,12,28)   62.5542   estimate D2E/DX2   !

! D65   D(23,7,12,29)   -55.1994   estimate D2E/DX2   !

! D66   D(23,7,12,30)   -173.0904   estimate D2E/DX2   !

! D67   D(2,8,10,9)   105.61   estimate D2E/DX2   !

! D68   D(2,8,10,25)   -74.4405   estimate D2E/DX2   !

! D69   D(6,8,10,9)   -12.7163   estimate D2E/DX2   !

! D70   D(6,8,10,25)   167.2331   estimate D2E/DX2   !

! D71   D(13,8,10,9)   -138.1492   estimate D2E/DX2   !

! D72   D(13,8,10,25)   41.8003   estimate D2E/DX2   !

! D73   D(2,8,13,31)   -63.7515   estimate D2E/DX2   !

! D74   D(2,8,13,32)   53.7934   estimate D2E/DX2   !

! D75   D(2,8,13,33)   174.9506   estimate D2E/DX2   !

! D76   D(6,8,13,31)   55.1255   estimate D2E/DX2   !

! D77   D(6,8,13,32)   172.6704   estimate D2E/DX2   !

! D78   D(6,8,13,33)   -66.1725   estimate D2E/DX2   !

! D79   D(10,8,13,31)   -179.0057   estimate D2E/DX2   !

! D80   D(10,8,13,32)   -61.4609   estimate D2E/DX2   !

! D81   D(10,8,13,33)   59.6963   estimate D2E/DX2   !

! D82   D(4,9,10,8)   -1.8371   estimate D2E/DX2   !

! D83   D(4,9,10,25)   178.2136   estimate D2E/DX2   !

! D84   D(24,9,10,8)   177.5346   estimate D2E/DX2   !

! D85   D(24,9,10,25)   -2.4146   estimate D2E/DX2   !

! D86   D(7,11,14,3)   30.4428   estimate D2E/DX2   !

! D87 D(7,11,14,15) -152.6068 estimate D2E/DX2 !

! D88 D(26,11,14,3) 155.9871 estimate D2E/DX2 !

! D89 D(26,11,14,15) -27.0624 estimate D2E/DX2 !

! D90 D(27,11,14,3) -89.2213 estimate D2E/DX2 !

! D91 D(27,11,14,15) 87.7292 estimate D2E/DX2 !

! D92 D(3,14,15,16) -33.8586 estimate D2E/DX2 !

! D93 D(3,14,15,36) 149.1447 estimate D2E/DX2 !

! D94 D(11,14,15,16) 149.1647 estimate D2E/DX2 !

! D95 D(11,14,15,36) -27.8319 estimate D2E/DX2 !

! D96 D(14,15,16,17) -179.9993 estimate D2E/DX2 !

! D97 D(14,15,16,18) 1.0586 estimate D2E/DX2 !

! D98 D(36,15,16,17) -3.1115 estimate D2E/DX2 !

! D99 D(36,15,16,18) 177.9464 estimate D2E/DX2 !

! D100 D(15,16,17,37) -1.0364 estimate D2E/DX2 !

! D101 D(15,16,17,38) 118.7594 estimate D2E/DX2 !

! D102 D(15,16,17,39) -120.8008 estimate D2E/DX2 !

! D103 D(18,16,17,37) 177.9577 estimate D2E/DX2 !

! D104 D(18,16,17,38) -62.2466 estimate D2E/DX2 !

! D105 D(18,16,17,39) 58.1932 estimate D2E/DX2 !

! D106 D(15,16,18,40) 83.8437 estimate D2E/DX2 !

! D107 D(15,16,18,41) -157.2992 estimate D2E/DX2 !

! D108 D(15,16,18,42) -39.6304 estimate D2E/DX2 !

! D109 D(17,16,18,40) -95.2169 estimate D2E/DX2 !

! D110 D(17,16,18,41) 23.6402 estimate D2E/DX2 !

! D111 D(17,16,18,42) 141.309 estimate D2E/DX2 !

Trust Radius=3.00D-01 FncErr=1.00D-07 GrdErr=1.00D-06

Number of steps in this run= 240 maximum allowed number of steps= 252.

GradGradGradGradGradGradGradGradGradGradGradGradGradGradGradGradGradGrad

Input orientation:

Center Atomic Atomic Coordinates (Angstroms)

Number	Number	Type	X	Y	Z
1	8	0	1.253300	2.213800	-0.319200
2	8	0	3.882800	0.216400	1.270000
3	8	0	-2.379500	-1.016900	-1.409900
4	6	0	0.657600	-0.727300	-0.443400
5	6	0	0.839100	0.128200	0.825700
6	6	0	1.818000	1.287900	0.607600
7	6	0	-0.039300	-2.042700	-0.090900
8	6	0	3.203600	0.779400	0.140500
9	6	0	1.962500	-0.986200	-1.150300
10	6	0	3.098600	-0.328300	-0.878100
11	6	0	-1.368500	-1.794400	0.638200
12	6	0	0.881500	-2.902300	0.776800
13	6	0	4.072500	1.919000	-0.403100
14	6	0	-2.351700	-0.977000	-0.183000
15	6	0	-3.334200	-0.171400	0.597200
16	6	0	-3.861700	0.999100	0.197600
17	6	0	-4.852600	1.828000	0.966300
18	6	0	-3.482100	1.574500	-1.141900
19	1	0	0.038600	-0.164100	-1.152800
20	1	0	-0.129300	0.556600	1.111800
21	1	0	1.192700	-0.451500	1.681900
22	1	0	1.935700	1.822700	1.558700
23	1	0	-0.243800	-2.595300	-1.018300
24	1	0	1.976200	-1.743000	-1.930800
25	1	0	3.998700	-0.605700	-1.421500
26	1	0	-1.212400	-1.323000	1.614400
27	1	0	-1.855500	-2.758500	0.835100
28	1	0	1.852800	-3.095800	0.312500
29	1	0	0.416800	-3.884600	0.928300
30	1	0	1.049600	-2.492800	1.776300
31	1	0	4.166400	2.718900	0.340500

32	1	0	5.092300	1.572000	-0.609100
33	1	0	3.665500	2.347400	-1.325100
34	1	0	0.401000	2.510800	0.042500
35	1	0	3.388100	-0.562300	1.576400
36	1	0	-3.586700	-0.563500	1.578600
37	1	0	-5.122300	1.350200	1.913900
38	1	0	-4.432800	2.812800	1.195400
39	1	0	-5.770700	1.962100	0.385400
40	1	0	-4.095400	1.137600	-1.936300
41	1	0	-3.649600	2.657900	-1.154200
42	1	0	-2.421200	1.441200	-1.375200

Distance matrix (angstroms):

		1	2	3	4	5
1	O	0.000000				
2	O	3.664619	0.000000			
3	O	4.982397	6.922376	0.000000		
4	C	3.003390	3.772032	3.200307	0.000000	
5	C	2.414971	3.077221	4.082713	1.541245	0.000000
6	C	1.426582	2.418736	5.196289	2.551893	1.533205
7	C	4.454293	4.726357	2.875511	1.529772	2.514866
8	C	2.464244	1.433196	6.066419	3.015493	2.546453
9	C	3.381374	3.315366	4.349862	1.506486	2.531500
10	C	3.190576	2.350745	5.546761	2.511304	2.866473
11	C	4.884272	5.658502	2.412746	2.532517	2.933436
12	C	5.245372	4.356299	4.355515	2.503926	3.031191
13	C	2.835813	2.394597	7.159711	4.320425	3.895098
14	C	4.816200	6.511866	1.227863	3.030849	3.524223
15	C	5.251107	7.258659	2.377977	4.162492	4.190275
16	C	5.282594	7.857477	2.974093	4.880104	4.821877
17	C	6.251669	8.888009	4.456008	6.235312	5.941762
18	C	4.848665	7.867875	2.828941	4.787828	4.963467
19	H	2.797283	4.559892	2.576931	1.097089	2.154229

20	H	2.589528	4.029604	3.728039	2.164777	1.096895
21	H	3.333451	2.802212	4.758100	2.208913	1.092778
22	H	2.035961	2.540619	5.957925	3.484888	2.147359
23	H	5.085026	5.492795	2.684383	2.152314	3.462724
24	H	4.333140	4.209453	4.446423	2.232199	3.520324
25	H	4.086792	2.816639	6.391452	3.483449	3.946085
26	H	4.725192	5.333800	3.256104	2.843642	2.633758
27	H	5.976689	6.478214	2.889251	3.475055	3.948923
28	H	5.380548	4.001047	5.020045	2.758565	3.418353
29	H	6.280643	5.380345	4.637906	3.450810	4.036264
30	H	5.156035	3.952610	4.908042	2.863166	2.795996
31	H	3.029271	2.684568	7.737500	4.980208	4.244770
32	H	3.903059	2.613725	7.948049	4.998080	4.715180
33	H	2.616943	3.364955	6.918651	4.390745	4.187998
34	H	0.972343	4.346718	4.720732	3.284392	2.546000
35	H	3.982135	0.972102	6.510749	3.400360	2.745495
36	H	5.894119	7.516443	3.254848	4.704189	4.542358
37	H	6.810346	9.099007	4.916682	6.578762	6.181889
38	H	5.914774	8.711835	5.066580	6.413279	5.927621
39	H	7.063738	9.849876	4.857755	7.017323	6.873610
40	H	5.690502	8.647580	2.804154	5.319550	5.744286
41	H	4.993283	8.280985	3.896497	5.524200	5.519766
42	H	3.900512	6.945332	2.458699	3.879388	4.146985

		6	7	8	9	10
6	C	0.000000				
7	C	3.876901	0.000000			
8	C	1.548109	4.305136	0.000000		
9	C	2.877955	2.499145	2.514724	0.000000	
10	C	2.541524	3.661320	1.508499	1.340764	0.000000
11	C	4.433429	1.536233	5.270318	3.866197	4.940000
12	C	4.296910	1.529604	4.399084	2.924673	3.778849
13	C	2.550014	5.718342	1.532702	3.667505	2.494887
14	C	4.810532	2.547821	5.835319	4.421321	5.532608

15	C	5.354888	3.851183	6.622343	5.636727	6.601670
16	C	5.701798	4.893518	7.068946	6.299171	7.166931
17	C	6.702035	6.266406	8.166019	7.671069	8.442335
18	C	5.588734	5.103098	6.853854	6.016720	6.855352
19	H	2.893713	2.159361	3.546836	2.092187	3.076690
20	H	2.140326	2.865475	3.478690	3.445716	3.893853
21	H	2.137904	2.681895	2.816868	2.983262	3.193937
22	H	1.097477	4.643611	2.169640	3.902476	3.452120
23	H	4.687623	1.098753	4.961450	2.733932	4.041112
24	H	3.956622	2.745413	3.487018	1.087251	2.090295
25	H	3.529649	4.487862	2.233949	2.089124	1.087389
26	H	4.124775	2.191388	5.108182	4.223386	5.078061
27	H	5.469891	2.160655	6.212383	4.654029	5.777892
28	H	4.393759	2.202679	4.107483	2.569481	3.260154
29	H	5.368516	2.153925	5.489971	3.887221	4.806496
30	H	4.031128	2.207879	4.245333	3.415877	3.991153
31	H	2.762985	6.367641	2.174545	4.561516	3.451175
32	H	3.504584	6.298246	2.181124	4.078349	2.767371
33	H	2.875957	5.875523	2.195440	3.747486	2.771381
34	H	1.955176	4.576682	3.295742	4.011242	4.023063
35	H	2.612858	4.088832	1.973831	3.105949	2.482567
36	H	5.794938	4.190383	7.069631	6.198322	7.126284
37	H	7.062440	6.431787	8.531786	8.064892	8.842840
38	H	6.460908	6.673327	7.972586	7.799652	8.419495
39	H	7.621830	7.008155	9.055209	8.417436	9.246991
40	H	6.439124	5.474647	7.597158	6.467339	7.417701
41	H	5.905547	6.021670	7.222974	6.691423	7.384567
42	H	4.682501	4.411398	5.862909	5.015942	5.817769
		11	12	13	14	15
11	C	0.000000				
12	C	2.511803	0.000000			
13	C	6.669192	5.900812	0.000000		
14	C	1.519603	3.883501	7.050220	0.000000	

15	C	2.549468	5.026151	7.760772	1.490976	0.000000
16	C	3.770122	6.168820	8.009906	2.515935	1.344621
17	C	5.036704	7.435829	9.030003	3.929811	2.537592
18	C	4.357241	6.539431	7.598453	2.950838	2.468706
19	H	2.800980	3.454211	4.601489	2.704598	3.799781
20	H	2.699466	3.619106	4.669709	2.994578	3.326587
21	H	3.074481	2.631059	4.273142	4.039403	4.663459
22	H	4.984824	4.903910	2.902386	5.408663	5.716009
23	H	2.156475	2.140779	6.275972	2.785653	4.246846
24	H	4.217751	3.142203	4.487603	4.729934	6.087779
25	H	5.870452	4.452396	2.723361	6.480689	7.618083
26	H	1.095240	2.753213	6.520049	2.156007	2.619716
27	H	1.097920	2.741395	7.652015	2.111039	2.989354
28	H	3.489484	1.093819	5.530585	4.734200	5.961387
29	H	2.764126	1.097183	6.987027	4.165779	5.288429
30	H	2.762289	1.093137	5.775092	4.207772	5.098713
31	H	7.147980	6.525244	1.096174	7.511282	8.042309
32	H	7.391234	6.298485	1.096739	7.879854	8.689103
33	H	6.808099	6.303014	1.095107	6.968696	7.683449
34	H	4.692626	5.483770	3.745490	4.448928	4.631804
35	H	5.002353	3.521080	3.247102	6.017705	6.804480
36	H	2.705527	5.106631	8.291760	2.190763	1.086575
37	H	5.060329	7.444620	9.499283	4.181994	2.691889
38	H	5.561178	7.815330	8.700242	4.538011	3.235771
39	H	5.792630	8.250294	9.874825	4.544331	3.245491
40	H	4.760321	6.960692	8.347208	3.253626	2.951530
41	H	5.314047	7.428018	7.793648	3.979984	3.342427
42	H	3.953614	5.865571	6.583419	2.697010	2.706365

		16	17	18	19	20
16	C	0.000000				
17	C	1.503282	0.000000			
18	C	1.506467	2.527259	0.000000		
19	H	4.288234	5.690596	3.926599	0.000000	

20	H	3.868123	4.893587	4.166120	2.382438	0.000000
21	H	5.463913	6.500297	5.825142	3.074097	1.757545
22	H	6.011718	6.814102	6.058663	3.859870	2.463119
23	H	5.242836	6.689190	5.281013	2.451239	3.805905
24	H	6.791927	8.232724	6.435931	2.617729	4.356456
25	H	8.184299	9.485252	7.797039	3.993695	4.980857
26	H	3.797139	4.857972	4.598286	3.250453	2.226793
27	H	4.307064	5.480493	5.032817	3.777601	3.747828
28	H	7.031139	8.344678	7.237965	3.746103	4.231740
29	H	6.533759	7.771861	7.020607	4.279735	4.478410
30	H	6.229489	7.359441	6.752416	3.876157	3.336196
31	H	8.211487	9.084475	7.874434	5.251701	4.870676
32	H	9.008501	10.072162	8.590938	5.371176	5.590853
33	H	7.797132	8.836194	7.191601	4.414943	4.852417
34	H	4.525473	5.377726	4.166285	2.952146	2.289873
35	H	7.543120	8.602028	7.691210	4.338922	3.720201
36	H	2.103451	2.774290	3.461662	4.556629	3.664170
37	H	2.158256	1.094978	3.475411	6.191336	5.118908
38	H	2.147386	1.094783	2.810727	5.862542	4.859788
39	H	2.146373	1.094685	2.778591	6.374539	5.859051
40	H	2.151122	3.078164	1.094573	4.404345	5.035715
41	H	2.150341	2.575357	1.096341	4.643975	4.684307
42	H	2.178117	3.397638	1.094398	2.945686	3.495782

		21	22	23	24	25
21	H	0.000000				
22	H	2.395665	0.000000			
23	H	3.735036	5.559665	0.000000		
24	H	3.915794	4.989235	2.547052	0.000000	
25	H	4.186706	4.362876	4.703178	2.375572	0.000000
26	H	2.559018	4.450738	3.080267	4.786649	6.073448
27	H	3.915462	5.990341	2.461565	4.833567	6.633133
28	H	3.050133	5.074596	2.533231	2.622535	3.716482
29	H	3.599460	5.939506	2.426507	3.897773	5.394697

30	H	2.048486	4.410903	3.081100	3.894018	4.741757
31	H	4.549031	2.695034	7.038247	5.464827	3.766395
32	H	4.954807	3.837490	6.782905	4.737742	2.568722
33	H	4.794855	3.403501	6.309282	4.466764	2.973401
34	H	3.477016	2.264431	5.254838	4.946714	4.979891
35	H	2.200724	2.792491	4.904718	3.960804	3.059758
36	H	4.781828	6.015916	4.695432	6.682287	8.157247
37	H	6.571086	7.082710	6.925643	8.645132	9.906719
38	H	6.522161	6.455236	7.189975	8.461898	9.467022
39	H	7.483002	7.796452	7.299787	8.894212	10.261562
40	H	6.601561	7.004181	5.441699	6.720284	8.295696
41	H	6.415583	6.265218	6.262112	7.184751	8.319797
42	H	5.097887	5.266487	4.600194	5.457559	6.738476

		26	27	28	29	30
26	H	0.000000				
27	H	1.755433	0.000000			
28	H	3.772693	3.760103	0.000000		
29	H	3.112366	2.537742	1.750289	0.000000	
30	H	2.551722	3.065299	1.775232	1.748327	0.000000
31	H	6.847720	8.155355	6.258136	7.616510	6.240014
32	H	7.285206	8.313295	5.756046	7.348359	6.209360
33	H	6.775427	7.824201	5.966242	7.380358	6.315786
34	H	4.446566	5.786668	5.797809	6.456472	5.335050
35	H	4.663122	5.733076	3.220747	4.504035	3.038976
36	H	2.493075	2.892727	6.132187	5.242195	5.025591
37	H	4.745842	5.358844	8.425166	7.684801	7.271859
38	H	5.258458	6.149120	8.671784	8.273157	7.651375
39	H	5.751558	6.149397	9.149067	8.530170	8.264214
40	H	5.193622	5.279906	7.639363	7.334056	7.309872
41	H	5.427030	6.042640	8.095226	7.979770	7.563070
42	H	4.247318	4.779427	6.457536	6.459451	6.120029

		31	32	33	34	35
31	H	0.000000				

32	H	1.753400	0.000000			
33	H	1.778521	1.774728	0.000000		
34	H	3.782902	4.828480	3.543161	0.000000	
35	H	3.591584	3.497991	4.118496	4.551874	0.000000
36	H	8.509853	9.201708	8.336619	5.264283	6.974800
37	H	9.519920	10.523915	9.418649	5.946089	8.729174
38	H	8.642101	9.773604	8.494232	4.978554	8.526602
39	H	9.965978	10.915401	9.597715	6.205524	9.574690
40	H	8.714452	9.293223	7.878372	5.100876	8.439875
41	H	7.957871	8.825935	7.323681	4.226238	8.207014
42	H	6.926227	7.553589	6.153993	3.334476	6.817178
		36	37	38	39	40
36	H	0.000000				
37	H	2.476437	0.000000			
38	H	3.501732	1.769421	0.000000		
39	H	3.545735	1.769507	1.780384	0.000000	
40	H	3.937898	3.990459	3.567587	2.979383	0.000000
41	H	4.224875	3.645842	2.481535	2.711747	1.766842
42	H	3.755283	4.257041	3.540594	3.819712	1.791634
		41	42			
41	H	0.000000				
42	H	1.743034	0.000000			

Stoichiometry C15H24O3

Framework group C1[X(C15H24O3)]

Deg. of freedom 120

Full point group C1 NOp 1

Largest Abelian subgroup C1 NOp 1

Largest concise Abelian subgroup C1 NOp 1

Standard orientation:

Center Atomic Atomic Coordinates (Angstroms)

Number Number Type X Y Z

1	8	0	1.199324	-2.205277	0.279485
2	8	0	3.866055	-0.203098	-1.240140
3	8	0	-2.379676	1.050568	1.468819
4	6	0	0.653998	0.740092	0.498051
5	6	0	0.823404	-0.076862	-0.797836
6	6	0	1.781775	-1.259724	-0.615957
7	6	0	-0.019460	2.078039	0.187318
8	6	0	3.174936	-0.790585	-0.130516
9	6	0	1.961685	0.953425	1.214952
10	6	0	3.086873	0.285193	0.923294
11	6	0	-1.351214	1.876437	-0.551465
12	6	0	0.917776	2.949282	-0.650663
13	6	0	4.022994	-1.962005	0.377177
14	6	0	-2.350019	1.049947	0.241314
15	6	0	-3.344596	0.287097	-0.566081
16	6	0	-3.892962	-0.886459	-0.205434
17	6	0	-4.896362	-1.672821	-1.002102
18	6	0	-3.526088	-1.511396	1.115285
19	1	0	0.023940	0.164972	1.187882
20	1	0	-0.151619	-0.479011	-1.099142
21	1	0	1.188691	0.524077	-1.634257
22	1	0	1.892242	-1.765393	-1.583713
23	1	0	-0.216358	2.603760	1.131831
24	1	0	1.986768	1.684203	2.019594
25	1	0	3.990477	0.529288	1.476767
26	1	0	-1.201212	1.434276	-1.542194
27	1	0	-1.821164	2.854662	-0.717749
28	1	0	1.891291	3.110868	-0.178861
29	1	0	0.470341	3.943840	-0.770948
30	1	0	1.080888	2.569528	-1.662656
31	1	0	4.104677	-2.738905	-0.391818
32	1	0	5.048176	-1.639494	0.595864
33	1	0	3.606778	-2.412957	1.284186

34	1	0	0.342799	-2.475667	-0.092946
35	1	0	3.385447	0.593530	-1.521888
36	1	0	-3.588283	0.715069	-1.534637
37	1	0	-5.155844	-1.160005	-1.934125
38	1	0	-4.493073	-2.656778	-1.262367
39	1	0	-5.817835	-1.809812	-0.427249
40	1	0	-4.133437	-1.089941	1.922498
41	1	0	-3.712207	-2.591577	1.092206
42	1	0	-2.463541	-1.404035	1.354394

Rotational constants (GHZ): 0.6663695 0.2347885 0.1992504

Standard basis: 6-31+G(2d,p) (6D, 7F)

There are 570 symmetry adapted cartesian basis functions of A symmetry.

There are 570 symmetry adapted basis functions of A symmetry.

570 basis functions, 852 primitive gaussians, 570 cartesian basis functions

69 alpha electrons 69 beta electrons

nuclear repulsion energy 1412.1180923296 Hartrees.

NAtoms= 42 NActive= 42 NUniq= 42 SFac= 1.00D+00 NAtFMM= 60 NAOKFM=F Big=F

Integral buffers will be 262144 words long.

Raffenetti 2 integral format.

Two-electron integral symmetry is turned on.

Polarizable Continuum Model (PCM)

=================================

Model : PCM (using non-symmetric T matrix).

Atomic radii : SMD-Coulomb.

Polarization charges : Total charges.

Charge compensation : None.

Solution method : On-the-fly selection.

Cavity type : VdW (van der Waals Surface) (Alpha=1.000).

Cavity algorithm : GePol (No added spheres)

Default sphere list used, NSphG= 42.

Lebedev-Laikov grids with approx. 5.0 points / Ang**2.

Smoothing algorithm: Karplus/York (Gamma=1.0000).

Polarization charges: spherical gaussians, with

point-specific exponents (IZeta= 3).

Self-potential: point-specific (ISelfS= 7).

Self-field : sphere-specific E.n sum rule (ISelfD= 2).

1st derivatives : Analytical E(r).r(x)/FMM algorithm (CHGder, D1EAlg=3).

Cavity 1st derivative terms included.

Solvent : Water, Eps= 78.355300 Eps(inf)= 1.777849

Atomic radii for non-electrostatic terms: SMD-CDS.

Nuclear repulsion after PCM non-electrostatic terms = 1412.1291656598 Hartrees.

One-electron integrals computed using PRISM.

NBasis= 570 RedAO= T EigKep= 2.70D-06 NBF= 570

NBsUse= 570 1.00D-06 EigRej= -1.00D+00 NBFU= 570

ExpMin= 4.38D-02 ExpMax= 5.48D+03 ExpMxC= 8.25D+02 IAcc=2 IRadAn= 4 AccDes= 0.00D+00

Harris functional with IExCor= 402 and IRadAn= 4 diagonalized for initial guess.

HarFok: IExCor= 402 AccDes= 0.00D+00 IRadAn= 4 IDoV= 1 UseB2=F ITyADJ=14

ICtDFT= 3500011 ScaDFX= 1.000000 1.000000 1.000000 1.000000

FoFCou: FMM=F IPFlag= 0 FMFlag= 100000 FMFlg1= 0

NFxFlg= 0 DoJE=T BraDBF=F KetDBF=T FulRan=T

wScrn= 0.000000 ICntrl= 500 IOpCl= 0 I1Cent= 200000004 NGrid= 0

NMat0= 1 NMatS0= 1 NMatT0= 0 NMatD0= 1 NMtDS0= 0 NMtDT0= 0

Petite list used in FoFCou.

Requested convergence on RMS density matrix=1.00D-08 within 128 cycles.

Requested convergence on MAX density matrix=1.00D-06.

Requested convergence on energy=1.00D-06.

No special actions if energy rises.

Inv3: Mode=1 IEnd= 21902412.

Iteration 1 A*A^-1 deviation from unit magnitude is 2.89D-15 for 1607.

Iteration 1 A*A^-1 deviation from orthogonality is 2.98D-15 for 2663 1547.

Iteration 1 A^-1*A deviation from unit magnitude is 2.44D-15 for 1308.

Iteration 1 A^-1*A deviation from orthogonality is 1.65D-09 for 2658 2645.

Iteration 2 A*A^-1 deviation from unit magnitude is 3.11D-15 for 526.

Iteration 2 A*A^-1 deviation from orthogonality is 4.97D-15 for 1952 67.

Iteration 2 A^-1*A deviation from unit magnitude is 1.55D-15 for 344.

Iteration 2 A^-1*A deviation from orthogonality is 4.26D-16 for 577 543.

Error on total polarization charges = 0.08079

SCF Done: E(RB3LYP) = -811.782446437 A.U. after 13 cycles

NFock= 13 Conv=0.89D-08 -V/T= 2.0079

SMD-CDS (non-electrostatic) energy (kcal/mol) = 6.95

(included in total energy above)

****

Population analysis using the SCF density.

****

Orbital symmetries:

Occupied (A) (A) (A) (A) (A) (A) (A) (A) (A) (A) (A) (A)

(A) (A) (A) (A) (A) (A) (A) (A) (A) (A) (A) (A)

(A) (A) (A) (A) (A) (A) (A) (A) (A) (A) (A) (A)

(A) (A) (A) (A) (A) (A) (A) (A) (A) (A) (A) (A)

(A) (A) (A) (A) (A) (A) (A) (A) (A) (A) (A) (A)

(A) (A) (A) (A) (A) (A) (A) (A) (A)

Virtual (A) (A) (A) (A) (A) (A) (A) (A) (A) (A) (A) (A)

(A) (A) (A) (A) (A) (A) (A) (A) (A) (A) (A) (A)

(A) (A) (A) (A) (A) (A) (A) (A) (A) (A) (A) (A)

(A) (A) (A) (A) (A) (A) (A) (A) (A) (A) (A) (A)

(A) (A) (A) (A) (A) (A) (A) (A) (A) (A) (A) (A)

(A) (A) (A) (A) (A) (A) (A) (A) (A) (A) (A) (A)

(A) (A) (A) (A) (A) (A) (A) (A) (A) (A) (A) (A)

(A) (A) (A) (A) (A) (A) (A) (A) (A) (A) (A) (A)

(A) (A) (A) (A) (A) (A) (A) (A) (A) (A) (A) (A)

(A) (A) (A) (A) (A) (A) (A) (A) (A) (A) (A) (A)
(A) (A) (A) (A) (A) (A) (A) (A) (A) (A) (A) (A)
(A) (A) (A) (A) (A) (A) (A) (A) (A) (A) (A) (A)
(A) (A) (A) (A) (A) (A) (A) (A) (A) (A) (A) (A)
(A) (A) (A) (A) (A) (A) (A) (A) (A) (A) (A) (A)
(A) (A) (A) (A) (A) (A) (A) (A) (A) (A) (A) (A)
(A) (A) (A) (A) (A) (A) (A) (A) (A) (A) (A) (A)
(A) (A) (A) (A) (A) (A) (A) (A) (A) (A) (A) (A)
(A) (A) (A) (A) (A) (A) (A) (A) (A) (A) (A) (A)
(A) (A) (A) (A) (A) (A) (A) (A) (A) (A) (A) (A)
(A) (A) (A) (A) (A) (A) (A) (A) (A) (A) (A) (A)
(A) (A) (A) (A) (A) (A) (A) (A) (A) (A) (A) (A)
(A) (A) (A) (A) (A) (A) (A) (A) (A) (A) (A) (A)
(A) (A) (A) (A) (A) (A) (A) (A) (A) (A) (A) (A)
(A) (A) (A) (A) (A) (A) (A) (A) (A) (A) (A) (A)
(A) (A) (A) (A) (A) (A) (A) (A) (A) (A) (A) (A)
(A) (A) (A) (A) (A) (A) (A) (A) (A) (A) (A) (A)
(A) (A) (A) (A) (A) (A) (A) (A) (A) (A) (A) (A)
(A) (A) (A) (A) (A) (A) (A) (A) (A) (A) (A) (A)
(A) (A) (A) (A) (A) (A) (A) (A) (A) (A) (A) (A)
(A) (A) (A) (A) (A) (A) (A) (A) (A) (A) (A) (A)
(A) (A) (A) (A) (A) (A) (A) (A) (A) (A) (A) (A)
(A) (A) (A) (A) (A) (A) (A) (A) (A) (A) (A) (A)
(A) (A) (A) (A) (A) (A) (A) (A) (A) (A) (A) (A)
(A) (A) (A) (A) (A) (A) (A) (A) (A) (A) (A) (A)
(A) (A) (A) (A) (A) (A) (A) (A) (A) (A) (A) (A)
(A) (A) (A) (A) (A) (A) (A) (A) (A) (A) (A) (A)
(A) (A) (A) (A) (A) (A) (A) (A) (A) (A) (A) (A)
(A) (A) (A) (A) (A) (A) (A) (A) (A) (A) (A) (A)
(A) (A) (A) (A) (A) (A) (A) (A) (A) (A) (A) (A)
(A) (A) (A) (A) (A) (A) (A) (A) (A) (A) (A) (A)
(A) (A) (A) (A) (A) (A) (A) (A) (A) (A) (A) (A)
(A) (A) (A) (A) (A) (A) (A) (A) (A)

The electronic state is 1-A.

Alpha occ. eigenvalues -- -19.15421 -19.15033 -19.14016 -10.27547 -10.24505

Alpha occ. eigenvalues -- -10.22975 -10.21413 -10.18495 -10.18229 -10.18181

Alpha occ. eigenvalues -- -10.18094 -10.18024 -10.17982 -10.17601 -10.17256

Alpha occ. eigenvalues -- -10.17221 -10.17193 -10.16417 -1.04669 -1.03692

Alpha occ. eigenvalues --   -1.02402 -0.83919 -0.81894 -0.79524 -0.75296

Alpha occ. eigenvalues --   -0.73787 -0.72638 -0.69262 -0.68329 -0.67656

Alpha occ. eigenvalues --   -0.62282 -0.60302 -0.58355 -0.54801 -0.54309

Alpha occ. eigenvalues --   -0.50966 -0.50632 -0.49062 -0.47943 -0.47240

Alpha occ. eigenvalues --   -0.46101 -0.45234 -0.44582 -0.44065 -0.43168

Alpha occ. eigenvalues --   -0.42621 -0.41630 -0.40520 -0.40333 -0.39522

Alpha occ. eigenvalues --   -0.38986 -0.38398 -0.38365 -0.37273 -0.37047

Alpha occ. eigenvalues --   -0.36269 -0.35929 -0.35041 -0.34547 -0.34319

Alpha occ. eigenvalues --   -0.32929 -0.32223 -0.31741 -0.31518 -0.28115

Alpha occ. eigenvalues --   -0.27255 -0.25781 -0.25586 -0.24581

Alpha virt. eigenvalues --   -0.05153   0.00045   0.00603   0.01384   0.01765

Alpha virt. eigenvalues --   0.02217   0.02881   0.03371   0.03814   0.04285

Alpha virt. eigenvalues --   0.04525   0.05038   0.05400   0.05929   0.06355

Alpha virt. eigenvalues --   0.06705   0.06979   0.07398   0.07839   0.08119

Alpha virt. eigenvalues --   0.08333   0.08982   0.09328   0.09629   0.10196

Alpha virt. eigenvalues --   0.10534   0.10888   0.11268   0.11596   0.11993

Alpha virt. eigenvalues --   0.12116   0.12324   0.13245   0.13492   0.14187

Alpha virt. eigenvalues --   0.14268   0.14870   0.15198   0.15673   0.16278

Alpha virt. eigenvalues --   0.16443   0.16731   0.17696   0.18023   0.18183

Alpha virt. eigenvalues --   0.18724   0.19022   0.19131   0.19395   0.19835

Alpha virt. eigenvalues --   0.20024   0.20272   0.20578   0.20963   0.21256

Alpha virt. eigenvalues --   0.21511   0.21733   0.22135   0.22706   0.22822

Alpha virt. eigenvalues --   0.23327   0.23539   0.23752   0.23958   0.24174

Alpha virt. eigenvalues --   0.24662   0.25091   0.25843   0.25901   0.25998

Alpha virt. eigenvalues --   0.26322   0.26582   0.26881   0.27077   0.27650

Alpha virt. eigenvalues --   0.28104   0.28355   0.28488   0.28943   0.29286

Alpha virt. eigenvalues --   0.29919   0.30110   0.30797   0.31329   0.31416

Alpha virt. eigenvalues --   0.32005   0.32571   0.32951   0.33242   0.33773

Alpha virt. eigenvalues --	0.34388	0.34718	0.35275	0.35569	0.35731
Alpha virt. eigenvalues --	0.36367	0.36602	0.37658	0.38038	0.38706
Alpha virt. eigenvalues --	0.39222	0.39308	0.40092	0.40771	0.41637
Alpha virt. eigenvalues --	0.42417	0.43188	0.43752	0.44157	0.45376
Alpha virt. eigenvalues --	0.46352	0.46885	0.47832	0.48417	0.49391
Alpha virt. eigenvalues --	0.51918	0.53230	0.58913	0.59391	0.59748
Alpha virt. eigenvalues --	0.60475	0.62478	0.63110	0.63763	0.64096
Alpha virt. eigenvalues --	0.64438	0.64750	0.65325	0.65540	0.66133
Alpha virt. eigenvalues --	0.66731	0.66816	0.68050	0.68215	0.68473
Alpha virt. eigenvalues --	0.68923	0.69687	0.70241	0.70546	0.71400
Alpha virt. eigenvalues --	0.71929	0.72979	0.73392	0.74394	0.75641
Alpha virt. eigenvalues --	0.75736	0.76145	0.76806	0.78078	0.78292
Alpha virt. eigenvalues --	0.78713	0.79561	0.80776	0.81015	0.81427
Alpha virt. eigenvalues --	0.82364	0.82614	0.83624	0.83984	0.84620
Alpha virt. eigenvalues --	0.85029	0.85714	0.85996	0.86423	0.86968
Alpha virt. eigenvalues --	0.87691	0.88892	0.89583	0.89806	0.90202
Alpha virt. eigenvalues --	0.90513	0.91152	0.91634	0.92728	0.92914
Alpha virt. eigenvalues --	0.94001	0.95386	0.96126	0.96979	0.97707
Alpha virt. eigenvalues --	0.98922	0.99450	1.01330	1.01793	1.02505
Alpha virt. eigenvalues --	1.03804	1.04661	1.05148	1.05701	1.06334
Alpha virt. eigenvalues --	1.07579	1.08614	1.09334	1.09705	1.10729
Alpha virt. eigenvalues --	1.11288	1.11647	1.12308	1.12507	1.13116
Alpha virt. eigenvalues --	1.15073	1.15888	1.16731	1.16921	1.18613
Alpha virt. eigenvalues --	1.19591	1.19989	1.21832	1.22038	1.22630
Alpha virt. eigenvalues --	1.23485	1.24300	1.25766	1.26081	1.27502
Alpha virt. eigenvalues --	1.27922	1.28711	1.30172	1.30664	1.31406
Alpha virt. eigenvalues --	1.32910	1.33449	1.33625	1.34796	1.36327
Alpha virt. eigenvalues --	1.37064	1.37991	1.39379	1.40289	1.40775
Alpha virt. eigenvalues --	1.41902	1.44319	1.44625	1.46736	1.46868
Alpha virt. eigenvalues --	1.47905	1.48452	1.50070	1.51656	1.52260
Alpha virt. eigenvalues --	1.53463	1.54276	1.55766	1.57336	1.57718
Alpha virt. eigenvalues --	1.58131	1.58963	1.59192	1.59673	1.60799
Alpha virt. eigenvalues --	1.61933	1.62433	1.64393	1.65022	1.66607

Alpha virt. eigenvalues -- 1.67330 1.67906 1.68708 1.69151 1.71133

Alpha virt. eigenvalues -- 1.72017 1.72986 1.73388 1.74948 1.76318

Alpha virt. eigenvalues -- 1.77600 1.79020 1.80593 1.81950 1.83250

Alpha virt. eigenvalues -- 1.84674 1.85215 1.87506 1.88054 1.89274

Alpha virt. eigenvalues -- 1.90336 1.91419 1.92847 1.93831 1.94959

Alpha virt. eigenvalues -- 1.95615 1.97146 1.98341 1.99729 2.01526

Alpha virt. eigenvalues -- 2.02158 2.02632 2.03581 2.05220 2.05327

Alpha virt. eigenvalues -- 2.06631 2.07957 2.09378 2.10119 2.10597

Alpha virt. eigenvalues -- 2.11074 2.12002 2.13536 2.16003 2.16597

Alpha virt. eigenvalues -- 2.19686 2.21047 2.33279 2.34190 2.36071

Alpha virt. eigenvalues -- 2.36784 2.37831 2.39503 2.39824 2.43542

Alpha virt. eigenvalues -- 2.44013 2.44655 2.45293 2.48031 2.48168

Alpha virt. eigenvalues -- 2.51404 2.51899 2.52777 2.55749 2.58582

Alpha virt. eigenvalues -- 2.61151 2.62393 2.62717 2.63646 2.64450

Alpha virt. eigenvalues -- 2.66367 2.67192 2.68679 2.71747 2.73272

Alpha virt. eigenvalues -- 2.74426 2.74650 2.80127 2.81536 2.82883

Alpha virt. eigenvalues -- 2.85702 2.87224 2.88247 2.88629 2.89310

Alpha virt. eigenvalues -- 2.90073 2.91545 2.92234 2.92269 2.92829

Alpha virt. eigenvalues -- 2.93372 2.95017 2.95298 2.96709 2.97226

Alpha virt. eigenvalues -- 2.98121 2.99849 3.00553 3.01387 3.02106

Alpha virt. eigenvalues -- 3.02319 3.02700 3.04204 3.04947 3.05101

Alpha virt. eigenvalues -- 3.05573 3.07076 3.07758 3.08785 3.10173

Alpha virt. eigenvalues -- 3.11172 3.11552 3.12619 3.14297 3.15230

Alpha virt. eigenvalues -- 3.16043 3.17985 3.19947 3.20563 3.26152

Alpha virt. eigenvalues -- 3.39915 3.43835 3.45274 3.66538 3.73155

Alpha virt. eigenvalues -- 3.80169 3.88791 3.93032 4.01255 4.01919

Alpha virt. eigenvalues -- 4.10933 4.14202 4.16086 4.19020 4.20378

Alpha virt. eigenvalues -- 4.21657 4.26686 4.27404 4.28947 4.30202

Alpha virt. eigenvalues -- 4.31161 4.31884 4.33359 4.35458 4.36122

Alpha virt. eigenvalues -- 4.37048 4.37890 4.40051 4.40393 4.41581

Alpha virt. eigenvalues -- 4.43826 4.44896 4.46046 4.47267 4.48184

Alpha virt. eigenvalues -- 4.49651 4.50439 4.51989 4.52824 4.54629

Alpha virt. eigenvalues -- 4.54815 4.57015 4.57458 4.58209 4.59221

Alpha virt. eigenvalues -- 4.59833 4.60880 4.62112 4.62517 4.63967

Alpha virt. eigenvalues -- 4.65414 4.66144 4.67523 4.68851 4.69173

Alpha virt. eigenvalues -- 4.69807 4.70889 4.71896 4.72788 4.73864

Alpha virt. eigenvalues -- 4.74766 4.75466 4.75786 4.76606 4.77258

Alpha virt. eigenvalues -- 4.77604 4.78540 4.79392 4.79528 4.80350

Alpha virt. eigenvalues -- 4.80651 4.82178 4.82892 4.83191 4.84584

Alpha virt. eigenvalues -- 4.86552 4.86628 4.87238 4.89445 4.91023

Alpha virt. eigenvalues -- 4.91249 4.91949 4.93747 4.95091 4.96061

Alpha virt. eigenvalues -- 4.99404 5.00724 5.04510 5.04709 5.06162

Alpha virt. eigenvalues -- 5.09010 5.12492 5.14874 5.15523 5.16784

Alpha virt. eigenvalues -- 5.19952 5.24872 5.26435 5.34084 5.44314

Alpha virt. eigenvalues -- 5.48904 5.51749 5.62514 5.88069 6.15098

Alpha virt. eigenvalues -- 6.90655 6.92353 6.98388 11.12824 11.45953

Alpha virt. eigenvalues -- 11.65148 11.74298 11.79683 11.87926 12.00729

Alpha virt. eigenvalues -- 12.04096 12.21774 12.31259 12.48247 12.56260

Alpha virt. eigenvalues -- 12.72398 12.87562 13.17093 13.37427 13.44302

Alpha virt. eigenvalues -- 13.56101

Condensed to atoms (all electrons):

		1	2	3	4	5	6
1	O	8.320403	0.004226	-0.000863	-0.061609	0.009911	0.060268
2	O	0.004226	8.370278	0.000013	0.043501	0.078699	-0.059835
3	O	-0.000863	0.000013	8.263656	-0.015508	0.006563	0.004801
4	C	-0.061609	0.043501	-0.015508	12.816841	-0.885505	0.186184
5	C	0.009911	0.078699	0.006563	-0.885505	7.379303	-0.520604
6	C	0.060268	-0.059835	0.004801	0.186184	-0.520604	6.894474
7	C	0.051165	-0.021279	-0.126273	-4.803117	1.334152	-0.151229
8	C	-0.016181	0.047172	-0.000594	-0.944003	0.308522	-1.406097
9	C	0.022663	-0.057378	-0.029974	-3.948665	-0.623976	-0.127791
10	C	0.034090	0.066316	0.004334	0.153634	-0.262078	0.308238
11	C	-0.034072	0.007326	0.138361	2.574669	-0.751474	0.088123
12	C	-0.007637	0.013726	0.018252	0.848967	-0.437407	-0.009092
13	C	0.001430	-0.102656	-0.000266	-0.150218	-0.217567	0.507058
14	C	-0.001430	0.000442	0.371707	-0.806165	0.479573	0.028286

15	C	0.010419	-0.000164	-0.105827	0.259380	-0.149260	-0.063847
16	C	-0.005994	0.000139	-0.039832	-0.009529	0.041137	0.045668
17	C	0.000513	0.000029	-0.051274	-0.032837	0.046643	-0.002000
18	C	0.003129	-0.000023	0.000736	-0.019838	0.014203	-0.012320
19	H	0.003478	-0.000442	-0.009241	0.181525	0.035980	0.044822
20	H	-0.012487	0.000532	-0.000016	-0.075574	0.412873	-0.014943
21	H	0.004456	-0.008840	-0.000107	-0.150496	0.390341	0.037085
22	H	-0.030689	-0.001934	-0.000015	0.042307	0.016139	0.217702
23	H	0.000099	0.000024	0.004458	0.122277	-0.054934	-0.016597
24	H	0.000794	-0.000118	-0.000278	-0.146617	0.004504	-0.029702
25	H	-0.000874	0.005833	-0.000001	0.054828	-0.005040	0.012344
26	H	0.000108	-0.000005	0.001227	0.088514	-0.016072	-0.009862
27	H	-0.000007	0.000002	-0.007104	0.058639	-0.004120	0.002251
28	H	-0.000010	-0.000201	0.000244	0.003095	0.003764	0.006033
29	H	-0.000002	0.000004	0.000029	-0.017278	-0.000223	-0.000324
30	H	-0.000073	0.000207	0.000123	0.152074	-0.052443	-0.001861
31	H	0.001388	0.002253	0.000000	0.005672	-0.011879	0.013622
32	H	-0.000545	-0.001178	0.000000	0.008384	-0.003847	0.010042
33	H	-0.000516	0.003976	0.000000	0.009639	-0.002824	0.026361
34	H	0.271097	-0.000566	0.000025	-0.001882	-0.066159	0.034440
35	H	-0.000063	0.262294	-0.000001	-0.013737	0.012654	-0.050744
36	H	0.000005	0.000000	0.000248	0.004249	-0.001130	0.000401
37	H	0.000000	0.000000	-0.000134	-0.000445	0.000344	0.000113
38	H	0.000002	0.000000	0.000226	-0.000490	0.000753	-0.000096
39	H	0.000000	0.000000	-0.000233	0.000229	-0.000180	0.000017
40	H	-0.000009	0.000000	-0.001301	0.001314	-0.001826	0.000153
41	H	-0.000073	0.000000	0.001216	0.003968	-0.001010	0.000328
42	H	0.000414	0.000001	0.000413	-0.018165	0.012497	-0.006071

		7	8	9	10	11	12
1	O	0.051165	-0.016181	0.022663	0.034090	-0.034072	-0.007637
2	O	-0.021279	0.047172	-0.057378	0.066316	0.007326	0.013726
3	O	-0.126273	-0.000594	-0.029974	0.004334	0.138361	0.018252
4	C	-4.803117	-0.944003	-3.948665	0.153634	2.574669	0.848967

5	C	1.334152	0.308522	-0.623976	-0.262078	-0.751474	-0.437407
6	C	-0.151229	-1.406097	-0.127791	0.308238	0.088123	-0.009092
7	C	14.697179	0.087553	0.483031	0.481891	-5.124021	-2.545389
8	C	0.087553	9.611773	0.810534	-1.548245	-0.087670	0.078027
9	C	0.483031	0.810534	11.267288	-1.164768	-0.211085	0.068420
10	C	0.481891	-1.548245	-1.164768	7.994453	-0.110831	-0.144597
11	C	-5.124021	-0.087670	-0.211085	-0.110831	9.906421	1.313928
12	C	-2.545389	0.078027	0.068420	-0.144597	1.313928	6.907959
13	C	0.138471	-1.619173	0.148434	0.267839	-0.043141	-0.061820
14	C	1.900496	-0.020418	-0.436859	-0.013983	-2.260665	-0.546149
15	C	-0.024505	0.007238	0.159092	0.035462	0.402101	-0.072809
16	C	-0.317901	-0.024156	-0.131055	0.006551	-0.039890	0.022287
17	C	-0.011863	-0.002900	-0.024821	0.001323	-0.095023	-0.012564
18	C	-0.000332	0.003180	0.007696	0.000084	0.035189	0.007586
19	H	0.193116	-0.015028	0.014629	-0.040875	-0.178748	-0.070991
20	H	-0.002624	-0.016744	0.026698	0.015111	0.007490	0.002235
21	H	0.155162	0.077575	-0.017081	-0.051166	-0.075529	-0.066314
22	H	0.008488	0.084274	-0.016750	0.018881	-0.002942	0.001963
23	H	0.052012	0.002210	-0.042603	0.040924	0.174849	0.050774
24	H	0.010772	0.023581	0.427732	0.002276	0.014092	-0.005953
25	H	-0.003077	-0.070571	-0.089017	0.410073	-0.000447	0.000197
26	H	-0.229727	0.000868	-0.012269	0.000178	0.581491	-0.009347
27	H	-0.156090	-0.001203	-0.008472	-0.000779	0.427777	-0.003546
28	H	-0.085821	-0.000107	0.005648	-0.019415	-0.010192	0.466144
29	H	-0.186393	0.002144	0.038496	-0.010712	0.071635	0.486158
30	H	-0.317965	-0.013953	-0.056186	0.028036	0.118942	0.518616
31	H	-0.000063	-0.072265	-0.008815	0.014405	0.000117	0.000138
32	H	0.000634	-0.046101	-0.015316	-0.006574	-0.000016	-0.000030
33	H	0.000212	-0.124490	-0.012419	0.055807	-0.000090	-0.000225
34	H	-0.002095	0.013873	0.015830	-0.002642	-0.001344	-0.000233
35	H	-0.004239	0.084026	0.033328	-0.076681	-0.000922	0.001793
36	H	-0.011474	0.000063	-0.000562	-0.000226	0.055616	0.002616
37	H	-0.000997	-0.000003	-0.000058	0.000000	-0.002887	-0.000055

38	H	0.000261	0.000001	-0.000092	0.000004	0.001376	-0.000011
39	H	0.000230	0.000002	0.000028	0.000000	-0.000198	-0.000001
40	H	0.002504	0.000053	0.001958	-0.000009	0.003947	0.000110
41	H	-0.000929	0.000091	0.000307	-0.000055	0.003858	-0.000025
42	H	0.009547	-0.000719	-0.005570	0.001123	-0.011265	0.000087
		13	14	15	16	17	18
1	O	0.001430	-0.001430	0.010419	-0.005994	0.000513	0.003129
2	O	-0.102656	0.000442	-0.000164	0.000139	0.000029	-0.000023
3	O	-0.000266	0.371707	-0.105827	-0.039832	-0.051274	0.000736
4	C	-0.150218	-0.806165	0.259380	-0.009529	-0.032837	-0.019838
5	C	-0.217567	0.479573	-0.149260	0.041137	0.046643	0.014203
6	C	0.507058	0.028286	-0.063847	0.045668	-0.002000	-0.012320
7	C	0.138471	1.900496	-0.024505	-0.317901	-0.011863	-0.000332
8	C	-1.619173	-0.020418	0.007238	-0.024156	-0.002900	0.003180
9	C	0.148434	-0.436859	0.159092	-0.131055	-0.024821	0.007696
10	C	0.267839	-0.013983	0.035462	0.006551	0.001323	0.000084
11	C	-0.043141	-2.260665	0.402101	-0.039890	-0.095023	0.035189
12	C	-0.061820	-0.546149	-0.072809	0.022287	-0.012564	0.007586
13	C	6.543686	0.004499	0.005491	-0.001958	0.000123	-0.000651
14	C	0.004499	8.728088	-2.584951	1.059310	-0.169282	-0.118626
15	C	0.005491	-2.584951	11.167330	-2.948655	0.299552	-0.435923
16	C	-0.001958	1.059310	-2.948655	8.595355	-0.916646	0.352004
17	C	0.000123	-0.169282	0.299552	-0.916646	6.770481	-0.266864
18	C	-0.000651	-0.118626	-0.435923	0.352004	-0.266864	6.034429
19	H	0.018562	0.096880	0.001885	-0.007811	0.000233	-0.002019
20	H	-0.003136	-0.010565	-0.018463	0.015265	-0.001040	-0.000791
21	H	0.009342	0.018272	-0.000507	-0.000662	0.000202	0.000394
22	H	-0.069778	-0.000662	0.002013	-0.001240	-0.000048	0.000149
23	H	-0.000797	-0.082519	0.015691	0.000128	0.000075	0.000083
24	H	-0.008515	-0.000882	0.000791	-0.000112	0.000000	-0.000038
25	H	-0.015515	0.000139	-0.000030	0.000027	0.000001	-0.000004
26	H	-0.000246	-0.186179	0.125197	-0.019414	0.005713	0.000740
27	H	-0.000021	0.005549	-0.011296	0.022946	0.001208	-0.001007

28	H	-0.000192	-0.005024	-0.000309	-0.000324	-0.000001	-0.000016
29	H	-0.000061	-0.027519	0.000718	0.000358	-0.000011	0.000011
30	H	-0.000612	-0.025380	0.003188	0.001555	0.000013	0.000070
31	H	0.412939	0.000010	-0.000013	0.000010	0.000001	0.000004
32	H	0.415121	-0.000003	-0.000006	0.000000	0.000000	-0.000002
33	H	0.410446	0.000049	0.000010	-0.000005	0.000000	0.000012
34	H	0.002613	-0.003321	0.001657	0.000002	-0.000400	0.001194
35	H	0.021414	0.000032	0.000041	-0.000030	0.000001	-0.000001
36	H	0.000010	0.031370	0.229170	0.032839	-0.040099	-0.008975
37	H	0.000000	-0.001700	-0.159489	0.003062	0.497166	0.016520
38	H	0.000000	-0.020514	0.131108	-0.185972	0.461536	-0.035817
39	H	0.000000	0.008348	0.061214	-0.149067	0.460628	-0.027543
40	H	-0.000002	-0.005107	0.014524	-0.039496	-0.031916	0.409090
41	H	-0.000004	-0.026124	0.124743	-0.156717	-0.020612	0.420579
42	H	-0.000055	0.030544	-0.096385	-0.011257	-0.009069	0.456873

		19	20	21	22	23	24
1	O	0.003478	-0.012487	0.004456	-0.030689	0.000099	0.000794
2	O	-0.000442	0.000532	-0.008840	-0.001934	0.000024	-0.000118
3	O	-0.009241	-0.000016	-0.000107	-0.000015	0.004458	-0.000278
4	C	0.181525	-0.075574	-0.150496	0.042307	0.122277	-0.146617
5	C	0.035980	0.412873	0.390341	0.016139	-0.054934	0.004504
6	C	0.044822	-0.014943	0.037085	0.217702	-0.016597	-0.029702
7	C	0.193116	-0.002624	0.155162	0.008488	0.052012	0.010772
8	C	-0.015028	-0.016744	0.077575	0.084274	0.002210	0.023581
9	C	0.014629	0.026698	-0.017081	-0.016750	-0.042603	0.427732
10	C	-0.040875	0.015111	-0.051166	0.018881	0.040924	0.002276
11	C	-0.178748	0.007490	-0.075529	-0.002942	0.174849	0.014092
12	C	-0.070991	0.002235	-0.066314	0.001963	0.050774	-0.005953
13	C	0.018562	-0.003136	0.009342	-0.069778	-0.000797	-0.008515
14	C	0.096880	-0.010565	0.018272	-0.000662	-0.082519	-0.000882
15	C	0.001885	-0.018463	-0.000507	0.002013	0.015691	0.000791
16	C	-0.007811	0.015265	-0.000662	-0.001240	0.000128	-0.000112
17	C	0.000233	-0.001040	0.000202	-0.000048	0.000075	0.000000

18	C	-0.002019	-0.000791	0.000394	0.000149	0.000083	-0.000038
19	H	0.612247	-0.006380	0.004632	-0.000221	-0.007127	-0.001118
20	H	-0.006380	0.554547	-0.031778	-0.003446	-0.000130	-0.000120
21	H	0.004632	-0.031778	0.545878	-0.003142	0.000024	-0.000051
22	H	-0.000221	-0.003446	-0.003142	0.619253	0.000003	0.000015
23	H	-0.007127	-0.000130	0.000024	0.000003	0.620644	0.002819
24	H	-0.001118	-0.000120	-0.000051	0.000015	0.002819	0.570685
25	H	-0.000162	0.000020	-0.000003	-0.000107	-0.000004	-0.008727
26	H	-0.000130	-0.001758	0.000280	0.000008	0.004601	0.000000
27	H	-0.000051	0.000155	0.000009	0.000000	-0.004184	-0.000003
28	H	0.000067	-0.000005	0.000145	0.000000	-0.002283	-0.000044
29	H	-0.000124	-0.000010	-0.000021	0.000000	-0.004827	0.000030
30	H	-0.000169	0.000126	0.001034	0.000011	0.004840	0.000099
31	H	-0.000002	-0.000002	-0.000007	0.001745	0.000000	0.000003
32	H	0.000000	0.000002	-0.000003	-0.000067	0.000000	-0.000008
33	H	0.000012	-0.000003	-0.000003	-0.000019	0.000000	-0.000012
34	H	-0.000095	0.004122	0.000064	-0.005976	-0.000001	-0.000006
35	H	0.000033	-0.000112	0.002277	-0.000806	-0.000001	-0.000066
36	H	0.000003	0.000008	0.000005	0.000000	-0.000003	0.000000
37	H	0.000000	-0.000001	0.000000	0.000000	0.000000	0.000000
38	H	0.000000	-0.000001	0.000000	0.000000	0.000000	0.000000
39	H	0.000000	0.000000	0.000000	0.000000	0.000000	0.000000
40	H	-0.000010	0.000001	0.000000	0.000000	0.000000	0.000000
41	H	0.000000	-0.000002	0.000000	0.000000	0.000000	0.000000
42	H	0.000702	0.000089	0.000001	0.000000	-0.000001	0.000000

		25	26	27	28	29	30
1	O	-0.000874	0.000108	-0.000007	-0.000010	-0.000002	-0.000073
2	O	0.005833	-0.000005	0.000002	-0.000201	0.000004	0.000207
3	O	-0.000001	0.001227	-0.007104	0.000244	0.000029	0.000123
4	C	0.054828	0.088514	0.058639	0.003095	-0.017278	0.152074
5	C	-0.005040	-0.016072	-0.004120	0.003764	-0.000223	-0.052443
6	C	0.012344	-0.009862	0.002251	0.006033	-0.000324	-0.001861
7	C	-0.003077	-0.229727	-0.156090	-0.085821	-0.186393	-0.317965

8	C	-0.070571	0.000868	-0.001203	-0.000107	0.002144	-0.013953
9	C	-0.089017	-0.012269	-0.008472	0.005648	0.038496	-0.056186
10	C	0.410073	0.000178	-0.000779	-0.019415	-0.010712	0.028036
11	C	-0.000447	0.581491	0.427777	-0.010192	0.071635	0.118942
12	C	0.000197	-0.009347	-0.003546	0.466144	0.486158	0.518616
13	C	-0.015515	-0.000246	-0.000021	-0.000192	-0.000061	-0.000612
14	C	0.000139	-0.186179	0.005549	-0.005024	-0.027519	-0.025380
15	C	-0.000030	0.125197	-0.011296	-0.000309	0.000718	0.003188
16	C	0.000027	-0.019414	0.022946	-0.000324	0.000358	0.001555
17	C	0.000001	0.005713	0.001208	-0.000001	-0.000011	0.000013
18	C	-0.000004	0.000740	-0.001007	-0.000016	0.000011	0.000070
19	H	-0.000162	-0.000130	-0.000051	0.000067	-0.000124	-0.000169
20	H	0.000020	-0.001758	0.000155	-0.000005	-0.000010	0.000126
21	H	-0.000003	0.000280	0.000009	0.000145	-0.000021	0.001034
22	H	-0.000107	0.000008	0.000000	0.000000	0.000000	0.000011
23	H	-0.000004	0.004601	-0.004184	-0.002283	-0.004827	0.004840
24	H	-0.008727	0.000000	-0.000003	-0.000044	0.000030	0.000099
25	H	0.566739	0.000000	0.000000	0.000029	0.000000	-0.000001
26	H	0.000000	0.517752	-0.029837	0.000023	-0.000285	0.002288
27	H	0.000000	-0.029837	0.514815	0.000028	0.001941	-0.000275
28	H	0.000029	0.000023	0.000028	0.521267	-0.026434	-0.029397
29	H	0.000000	-0.000285	0.001941	-0.026434	0.539651	-0.028577
30	H	-0.000001	0.002288	-0.000275	-0.029397	-0.028577	0.527532
31	H	0.000018	0.000000	0.000000	0.000000	0.000000	0.000000
32	H	0.001908	0.000000	0.000000	0.000000	0.000000	0.000000
33	H	-0.000054	0.000000	0.000000	0.000000	0.000000	0.000000
34	H	0.000008	-0.000003	0.000000	0.000000	0.000000	0.000000
35	H	-0.000295	0.000002	0.000000	-0.000056	0.000004	-0.000055
36	H	0.000000	-0.001169	-0.000148	0.000000	0.000000	0.000001
37	H	0.000000	0.000001	0.000000	0.000000	0.000000	0.000000
38	H	0.000000	0.000002	0.000000	0.000000	0.000000	0.000000
39	H	0.000000	0.000000	0.000000	0.000000	0.000000	0.000000
40	H	0.000000	-0.000001	0.000002	0.000000	0.000000	0.000000

41	H	0.000000	0.000000	0.000000	0.000000	0.000000	0.000000
42	H	0.000000	0.000029	-0.000007	0.000000	0.000000	0.000000

		31	32	33	34	35	36
1	O	0.001388	-0.000545	-0.000516	0.271097	-0.000063	0.000005
2	O	0.002253	-0.001178	0.003976	-0.000566	0.262294	0.000000
3	O	0.000000	0.000000	0.000000	0.000025	-0.000001	0.000248
4	C	0.005672	0.008384	0.009639	-0.001882	-0.013737	0.004249
5	C	-0.011879	-0.003847	-0.002824	-0.066159	0.012654	-0.001130
6	C	0.013622	0.010042	0.026361	0.034440	-0.050744	0.000401
7	C	-0.000063	0.000634	0.000212	-0.002095	-0.004239	-0.011474
8	C	-0.072265	-0.046101	-0.124490	0.013873	0.084026	0.000063
9	C	-0.008815	-0.015316	-0.012419	0.015830	0.033328	-0.000562
10	C	0.014405	-0.006574	0.055807	-0.002642	-0.076681	-0.000226
11	C	0.000117	-0.000016	-0.000090	-0.001344	-0.000922	0.055616
12	C	0.000138	-0.000030	-0.000225	-0.000233	0.001793	0.002616
13	C	0.412939	0.415121	0.410446	0.002613	0.021414	0.000010
14	C	0.000010	-0.000003	0.000049	-0.003321	0.000032	0.031370
15	C	-0.000013	-0.000006	0.000010	0.001657	0.000041	0.229170
16	C	0.000010	0.000000	-0.000005	0.000002	-0.000030	0.032839
17	C	0.000001	0.000000	0.000000	-0.000400	0.000001	-0.040099
18	C	0.000004	-0.000002	0.000012	0.001194	-0.000001	-0.008975
19	H	-0.000002	0.000000	0.000012	-0.000095	0.000033	0.000003
20	H	-0.000002	0.000002	-0.000003	0.004122	-0.000112	0.000008
21	H	-0.000007	-0.000003	-0.000003	0.000064	0.002277	0.000005
22	H	0.001745	-0.000067	-0.000019	-0.005976	-0.000806	0.000000
23	H	0.000000	0.000000	0.000000	-0.000001	-0.000001	-0.000003
24	H	0.000003	-0.000008	-0.000012	-0.000006	-0.000066	0.000000
25	H	0.000018	0.001908	-0.000054	0.000008	-0.000295	0.000000
26	H	0.000000	0.000000	0.000000	-0.000003	0.000002	-0.001169
27	H	0.000000	0.000000	0.000000	0.000000	0.000000	-0.000148
28	H	0.000000	0.000000	0.000000	0.000000	-0.000056	0.000000
29	H	0.000000	0.000000	0.000000	0.000000	0.000004	0.000000
30	H	0.000000	0.000000	0.000000	0.000000	-0.000055	0.000001

31	H	0.518444	-0.028213	-0.025210	-0.000061	-0.000118	0.000000
32	H	-0.028213	0.512046	-0.025758	-0.000001	-0.000034	0.000000
33	H	-0.025210	-0.025758	0.495648	0.000128	-0.000138	0.000000
34	H	-0.000061	-0.000001	0.000128	0.358064	-0.000004	0.000000
35	H	-0.000118	-0.000034	-0.000138	-0.000004	0.355661	0.000000
36	H	0.000000	0.000000	0.000000	0.000000	0.000000	0.554920
37	H	0.000000	0.000000	0.000000	0.000000	0.000000	0.003823
38	H	0.000000	0.000000	0.000000	0.000000	0.000000	-0.000038
39	H	0.000000	0.000000	0.000000	0.000000	0.000000	0.000063
40	H	0.000000	0.000000	0.000000	0.000000	0.000000	-0.000134
41	H	0.000000	0.000000	0.000000	0.000004	0.000000	-0.000114
42	H	0.000000	0.000000	0.000000	0.000049	0.000000	-0.000077

		37	38	39	40	41	42
1	O	0.000000	0.000002	0.000000	-0.000009	-0.000073	0.000414
2	O	0.000000	0.000000	0.000000	0.000000	0.000000	0.000001
3	O	-0.000134	0.000226	-0.000233	-0.001301	0.001216	0.000413
4	C	-0.000445	-0.000490	0.000229	0.001314	0.003968	-0.018165
5	C	0.000344	0.000753	-0.000180	-0.001826	-0.001010	0.012497
6	C	0.000113	-0.000096	0.000017	0.000153	0.000328	-0.006071
7	C	-0.000997	0.000261	0.000230	0.002504	-0.000929	0.009547
8	C	-0.000003	0.000001	0.000002	0.000053	0.000091	-0.000719
9	C	-0.000058	-0.000092	0.000028	0.001958	0.000307	-0.005570
10	C	0.000000	0.000004	0.000000	-0.000009	-0.000055	0.001123
11	C	-0.002887	0.001376	-0.000198	0.003947	0.003858	-0.011265
12	C	-0.000055	-0.000011	-0.000001	0.000110	-0.000025	0.000087
13	C	0.000000	0.000000	0.000000	-0.000002	-0.000004	-0.000055
14	C	-0.001700	-0.020514	0.008348	-0.005107	-0.026124	0.030544
15	C	-0.159489	0.131108	0.061214	0.014524	0.124743	-0.096385
16	C	0.003062	-0.185972	-0.149067	-0.039496	-0.156717	-0.011257
17	C	0.497166	0.461536	0.460628	-0.031916	-0.020612	-0.009069
18	C	0.016520	-0.035817	-0.027543	0.409090	0.420579	0.456873
19	H	0.000000	0.000000	0.000000	-0.000010	0.000000	0.000702
20	H	-0.000001	-0.000001	0.000000	0.000001	-0.000002	0.000089

21	H	0.000000	0.000000	0.000000	0.000000	0.000000	0.000001
22	H	0.000000	0.000000	0.000000	0.000000	0.000000	0.000000
23	H	0.000000	0.000000	0.000000	0.000000	0.000000	-0.000001
24	H	0.000000	0.000000	0.000000	0.000000	0.000000	0.000000
25	H	0.000000	0.000000	0.000000	0.000000	0.000000	0.000000
26	H	0.000001	0.000002	0.000000	-0.000001	0.000000	0.000029
27	H	0.000000	0.000000	0.000000	0.000002	0.000000	-0.000007
28	H	0.000000	0.000000	0.000000	0.000000	0.000000	0.000000
29	H	0.000000	0.000000	0.000000	0.000000	0.000000	0.000000
30	H	0.000000	0.000000	0.000000	0.000000	0.000000	0.000000
31	H	0.000000	0.000000	0.000000	0.000000	0.000000	0.000000
32	H	0.000000	0.000000	0.000000	0.000000	0.000000	0.000000
33	H	0.000000	0.000000	0.000000	0.000000	0.000000	0.000000
34	H	0.000000	0.000000	0.000000	0.000000	0.000004	0.000049
35	H	0.000000	0.000000	0.000000	0.000000	0.000000	0.000000
36	H	0.003823	-0.000038	0.000063	-0.000134	-0.000114	-0.000077
37	H	0.516424	-0.025572	-0.025737	-0.000142	0.000240	-0.000148
38	H	-0.025572	0.498754	-0.025214	-0.000037	0.001596	0.000054
39	H	-0.025737	-0.025214	0.498569	0.000315	-0.000126	0.000017
40	H	-0.000142	-0.000037	0.000315	0.505234	-0.028332	-0.022942
41	H	0.000240	0.001596	-0.000126	-0.028332	0.537112	-0.030430
42	H	-0.000148	0.000054	0.000017	-0.022942	-0.030430	0.506993

Mulliken charges:

		1
1	O	-0.626923
2	O	-0.652375
3	O	-0.427792
4	C	0.481786
5	C	-0.518999
6	C	-0.045803
7	C	0.520522
8	C	0.787860
9	C	-0.490231

10	C	-0.487398
11	C	-0.884857
12	C	-0.815789
13	C	-0.611096
14	C	0.590436
15	C	-0.385578
16	C	0.809077
17	C	-0.856172
18	C	-0.833166
19	H	0.131938
20	H	0.160855
21	H	0.158531
22	H	0.124892
23	H	0.119473
24	H	0.144174
25	H	0.141765
26	H	0.187284
27	H	0.192828
28	H	0.173343
29	H	0.161621
30	H	0.168190
31	H	0.175878
32	H	0.179566
33	H	0.189464
34	H	0.381615
35	H	0.374543
36	H	0.148741
37	H	0.179676
38	H	0.198181
39	H	0.198640
40	H	0.192059
41	H	0.170510
42	H	0.192729

Sum of Mulliken charges = 0.00000

Mulliken charges with hydrogens summed into heavy atoms:

		1
1	O	-0.245308
2	O	-0.277832
3	O	-0.427792
4	C	0.613724
5	C	-0.199612
6	C	0.079090
7	C	0.639995
8	C	0.787860
9	C	-0.346057
10	C	-0.345633
11	C	-0.504744
12	C	-0.312634
13	C	-0.066189
14	C	0.590436
15	C	-0.236837
16	C	0.809077
17	C	-0.279675
18	C	-0.277868

Electronic spatial extent (au): <R**2>= 5778.3736

Charge= 0.0000 electrons

Dipole moment (field-independent basis, Debye):

X= -4.2599 Y= 0.3036 Z= -4.9140 Tot= 6.5104

Quadrupole moment (field-independent basis, Debye-Ang):

XX= -117.8926 YY= -107.5187 ZZ= -116.4299

XY= 13.7510 XZ= 13.8104 YZ= -2.0266

Traceless Quadrupole moment (field-independent basis, Debye-Ang):

XX= -3.9456 YY= 6.4284 ZZ= -2.4828

XY= 13.7510 XZ= 13.8104 YZ= -2.0266

Octapole moment (field-independent basis, Debye-Ang**2):

XXX= -107.0015 YYY= -1.4304 ZZZ= -15.2946 XYY= -17.4896

XXY= -23.0815 XXZ= 1.7730 XZZ= 5.1190 YZZ= -0.2974

YYZ= -13.7769 XYZ= 8.3833

Hexadecapole moment (field-independent basis, Debye-Ang**3):

XXXX= -5503.8948 YYYY= -1724.2352 ZZZZ= -679.2936 XXXY= 156.1484

XXXZ= 209.3007 YYYX= 16.5089 YYYZ= 26.8304 ZZZX= 10.6383

ZZZY= -8.8834 XXYY= -1157.6624 XXZZ= -1057.7545 YYZZ= -401.9806

XXYZ= -40.8875 YYXZ= 3.1241 ZZXY= 32.3880

N-N= 1.412129165660D+03 E-N=-4.712593650925D+03 KE= 8.054211761012D+02

D1PCM: PCM CHGder 1st derivatives, ID1Alg=3 FixD1E=F DoIter=F DoCFld=F I1PDM=0.

Calling FoFJK, ICntrl= 2127 FMM=F ISym2X=0 I1Cent= 0 IOpClX= 0 NMat=1 NMatS=1 NMatT=0.

***** Axes restored to original set *****

Center	Atomic	Forces (Hartrees/Bohr)		
Number	Number	X	Y	Z
1	8	-0.000452514	0.007420713	-0.009924285
2	8	0.005668423	0.001054371	0.014808820
3	8	0.004783891	-0.003141244	-0.003737661
4	6	0.005167992	0.005883174	-0.000421130
5	6	0.003825548	0.009941301	-0.005129762
6	6	-0.009966742	-0.004074021	0.010146671
7	6	0.001795536	-0.005070298	0.003462625
8	6	-0.001600161	0.000781466	-0.013975749
9	6	0.006441415	0.007556739	0.004385426
10	6	-0.011566789	-0.002182397	-0.010243649
11	6	-0.000772118	-0.001961768	-0.007454904
12	6	-0.005244241	-0.017423854	0.002640062
13	6	0.004773369	0.001795731	0.006155417
14	6	-0.014735459	0.003008671	0.014092225
15	6	0.002261250	-0.005824203	0.005949416
16	6	0.000866225	0.005046008	-0.016224092
17	6	0.003832597	-0.012222340	0.010466728
18	6	0.001980419	0.014120029	-0.000525696

19	1	-0.002576674	-0.000854773	0.001510297
20	1	0.002021684	0.000274798	0.001567564
21	1	-0.000640803	-0.002549390	-0.001015893
22	1	0.006247751	-0.002054571	0.000847098
23	1	0.000072935	0.001599872	0.001104770
24	1	-0.001987483	-0.002356122	0.000585880
25	1	0.002639854	0.003653365	0.001300514
26	1	-0.001073183	-0.000854879	-0.001622363
27	1	-0.001396595	-0.000863007	0.000668533
28	1	0.000025793	0.002577152	-0.000756722
29	1	-0.000045429	-0.000988729	0.000835406
30	1	0.000465440	0.002105144	-0.000533679
31	1	-0.002174235	0.000944647	-0.000003285
32	1	-0.002100198	-0.003063579	-0.000997559
33	1	-0.000836759	-0.002246298	0.002351222
34	1	0.005563030	-0.002709547	-0.002094366
35	1	0.002796471	0.003684922	-0.002837263
36	1	0.001490307	-0.000146250	0.002207370
37	1	-0.000591619	0.000048663	0.000412685
38	1	-0.000672917	0.001716747	-0.002358083
39	1	0.000501863	0.002215844	-0.002322008
40	1	0.001730927	-0.000007085	-0.001622504
41	1	-0.003552820	-0.000267767	0.000436243
42	1	-0.002965981	-0.004567233	-0.002134316

Cartesian Forces: Max 0.017423854 RMS 0.005278542

GradGradGradGradGradGradGradGradGradGradGradGradGradGradGradGradGradGradGrad

Berny optimization.

FormGI is forming the generalized inverse of G from B-inverse, IUseBI=4.

Internal Forces: Max 0.024062570 RMS 0.004325413

Search for a local minimum.

Step number 1 out of a maximum of 240

All quantities printed in internal units (Hartrees-Bohrs-Radians)

Mixed Optimization -- RFO/linear search

Second derivative matrix not updated -- first step.

ITU= 0

Eigenvalues --- 0.00287 0.00333 0.00372 0.00374 0.00455

Eigenvalues --- 0.00507 0.00520 0.00664 0.00680 0.00723

Eigenvalues --- 0.00885 0.01232 0.01253 0.01266 0.01302

Eigenvalues --- 0.01340 0.01732 0.01893 0.02821 0.03149

Eigenvalues --- 0.03235 0.03698 0.03910 0.04245 0.04456

Eigenvalues --- 0.04636 0.04706 0.04849 0.04887 0.05017

Eigenvalues --- 0.05300 0.05310 0.05375 0.05590 0.05667

Eigenvalues --- 0.06177 0.06442 0.06919 0.06936 0.07230

Eigenvalues --- 0.07286 0.07318 0.08227 0.08633 0.09169

Eigenvalues --- 0.09479 0.12372 0.12897 0.15098 0.15989

Eigenvalues --- 0.15998 0.16000 0.16000 0.16000 0.16000

Eigenvalues --- 0.16000 0.16000 0.16000 0.16000 0.16000

Eigenvalues --- 0.16000 0.16000 0.16000 0.16000 0.16000

Eigenvalues --- 0.16000 0.16238 0.16389 0.16665 0.17597

Eigenvalues --- 0.19017 0.20573 0.21934 0.22004 0.24968

Eigenvalues --- 0.24996 0.24998 0.25000 0.26504 0.27479

Eigenvalues --- 0.28061 0.28855 0.29175 0.29444 0.29460

Eigenvalues --- 0.30405 0.30542 0.31205 0.31708 0.32035

Eigenvalues --- 0.33343 0.33820 0.33913 0.33963 0.33996

Eigenvalues --- 0.34006 0.34028 0.34045 0.34090 0.34109

Eigenvalues --- 0.34214 0.34229 0.34244 0.34266 0.34277

Eigenvalues --- 0.34289 0.34309 0.34375 0.34453 0.34494

Eigenvalues --- 0.35116 0.35132 0.35211 0.40533 0.41484

Eigenvalues --- 0.52901 0.52950 0.54676 0.56182 0.91982

RFO step: Lambda=-2.22606115D-02 EMin= 2.86652115D-03

Linear search not attempted -- first point.

Iteration 1 RMS(Cart)= 0.18449242 RMS(Int)= 0.00520280

Iteration 2 RMS(Cart)= 0.01381166 RMS(Int)= 0.00044934

Iteration 3 RMS(Cart)= 0.00010178 RMS(Int)= 0.00044845

Iteration 4 RMS(Cart)= 0.00000005 RMS(Int)= 0.00044845

Variable	Old X	-DE/DX	Delta X	Delta X	Delta X	New X
			(Linear)	(Quad)	(Total)	
R1	2.69585	0.00884	0.00000	0.02022	0.02022	2.71607
R2	1.83746	-0.00649	0.00000	-0.01176	-0.01176	1.82570
R3	2.70835	0.01159	0.00000	0.02710	0.02710	2.73544
R4	1.83701	-0.00527	0.00000	-0.00954	-0.00954	1.82746
R5	2.32033	0.00373	0.00000	0.00396	0.00396	2.32428
R6	2.91253	0.00405	0.00000	0.01042	0.01071	2.92325
R7	2.89085	0.02374	0.00000	0.07496	0.07496	2.96581
R8	2.84685	0.00173	0.00000	0.00632	0.00671	2.85355
R9	2.07320	0.00004	0.00000	0.00011	0.00011	2.07331
R10	2.89734	0.00175	0.00000	0.00961	0.00960	2.90694
R11	2.07283	-0.00127	0.00000	-0.00350	-0.00350	2.06933
R12	2.06505	0.00035	0.00000	0.00095	0.00095	2.06600
R13	2.92550	-0.00227	0.00000	-0.01163	-0.01203	2.91347
R14	2.07393	0.00040	0.00000	0.00111	0.00111	2.07504
R15	2.90306	0.00483	0.00000	0.01553	0.01553	2.91859
R16	2.89053	0.00606	0.00000	0.01911	0.01911	2.90965
R17	2.07634	-0.00175	0.00000	-0.00485	-0.00485	2.07149
R18	2.85065	0.00178	0.00000	0.00532	0.00500	2.85565
R19	2.89639	-0.00477	0.00000	-0.01520	-0.01520	2.88119
R20	2.53368	-0.00258	0.00000	-0.00356	-0.00350	2.53017
R21	2.05461	0.00120	0.00000	0.00320	0.00320	2.05781
R22	2.05487	0.00060	0.00000	0.00162	0.00162	2.05649
R23	2.87163	0.00077	0.00000	0.00235	0.00235	2.87399
R24	2.06970	-0.00197	0.00000	-0.00540	-0.00540	2.06431
R25	2.07477	0.00150	0.00000	0.00414	0.00414	2.07891
R26	2.06702	-0.00011	0.00000	-0.00031	-0.00031	2.06671
R27	2.07338	0.00102	0.00000	0.00282	0.00282	2.07620
R28	2.06573	0.00037	0.00000	0.00100	0.00100	2.06673
R29	2.07147	0.00050	0.00000	0.00137	0.00137	2.07284
R30	2.07254	-0.00079	0.00000	-0.00219	-0.00219	2.07035

R31	2.06945	-0.00255	0.00000	-0.00699	-0.00699	2.06246
R32	2.81754	-0.00611	0.00000	-0.01717	-0.01717	2.80036
R33	2.54097	0.00900	0.00000	0.01541	0.01541	2.55638
R34	2.05333	0.00170	0.00000	0.00454	0.00454	2.05787
R35	2.84079	-0.00338	0.00000	-0.00988	-0.00988	2.83091
R36	2.84681	0.00625	0.00000	0.01843	0.01843	2.86524
R37	2.06921	0.00048	0.00000	0.00132	0.00132	2.07053
R38	2.06884	0.00080	0.00000	0.00219	0.00219	2.07103
R39	2.06865	0.00108	0.00000	0.00296	0.00296	2.07161
R40	2.06844	0.00021	0.00000	0.00056	0.00056	2.06901
R41	2.07178	0.00028	0.00000	0.00077	0.00077	2.07255
R42	2.06811	-0.00186	0.00000	-0.00510	-0.00510	2.06301
A1	1.87921	-0.00122	0.00000	-0.00671	-0.00671	1.87250
A2	1.89854	-0.00129	0.00000	-0.00706	-0.00706	1.89148
A3	1.91898	0.01341	0.00000	0.08948	0.08969	2.00867
A4	1.96034	-0.00626	0.00000	-0.02244	-0.02348	1.93687
A5	1.89013	-0.00251	0.00000	-0.02418	-0.02367	1.86646
A6	1.93366	-0.00171	0.00000	-0.00087	-0.00201	1.93164
A7	1.91069	-0.00558	0.00000	-0.04474	-0.04415	1.86654
A8	1.84785	0.00195	0.00000	-0.00359	-0.00440	1.84345
A9	1.95833	0.00268	0.00000	0.01453	0.01463	1.97296
A10	1.90455	-0.00094	0.00000	0.01070	0.01063	1.91518
A11	1.97014	-0.00105	0.00000	-0.02168	-0.02171	1.94842
A12	1.88112	0.00019	0.00000	-0.00571	-0.00598	1.87514
A13	1.88190	-0.00117	0.00000	0.00574	0.00587	1.88778
A14	1.86345	0.00023	0.00000	-0.00403	-0.00398	1.85947
A15	1.90774	0.00450	0.00000	0.03229	0.03202	1.93977
A16	1.95137	-0.00701	0.00000	-0.05631	-0.05627	1.89510
A17	1.86418	0.00348	0.00000	0.03628	0.03442	1.89860
A18	1.94542	0.00209	0.00000	0.01000	0.01020	1.95561
A19	1.88997	0.00092	0.00000	0.01507	0.01463	1.90460
A20	1.90239	-0.00375	0.00000	-0.03449	-0.03435	1.86804
A21	1.94394	0.00381	0.00000	0.04110	0.04026	1.98420

A22	1.91739	0.00858	0.00000	0.06943	0.06906	1.98646
A23	1.89943	-0.00466	0.00000	-0.03627	-0.03502	1.86442
A24	1.92033	-0.00696	0.00000	-0.01887	-0.02233	1.89800
A25	1.89741	0.00005	0.00000	-0.03101	-0.03131	1.86610
A26	1.88411	-0.00106	0.00000	-0.02798	-0.02802	1.85609
A27	1.89168	0.00095	0.00000	0.00276	0.00253	1.89421
A28	1.85114	0.00478	0.00000	0.05414	0.05445	1.90560
A29	1.87856	-0.00559	0.00000	-0.04054	-0.04090	1.83767
A30	1.96344	-0.00367	0.00000	-0.02046	-0.02096	1.94247
A31	1.95001	0.00115	0.00000	-0.00459	-0.00461	1.94540
A32	1.92404	0.00240	0.00000	0.01036	0.01073	1.93478
A33	2.15841	0.00489	0.00000	0.02089	0.02136	2.17977
A34	2.05713	-0.00494	0.00000	-0.02421	-0.02444	2.03269
A35	2.06760	0.00004	0.00000	0.00331	0.00307	2.07067
A36	2.16082	0.00131	0.00000	0.00466	0.00440	2.16523
A37	2.05686	-0.00533	0.00000	-0.02797	-0.02787	2.02899
A38	2.06550	0.00402	0.00000	0.02331	0.02342	2.08892
A39	1.97173	0.01112	0.00000	0.04966	0.04956	2.02129
A40	1.94902	-0.00286	0.00000	-0.00566	-0.00591	1.94311
A41	1.90390	-0.00195	0.00000	-0.00746	-0.00722	1.89668
A42	1.92023	-0.00351	0.00000	-0.00857	-0.00896	1.91128
A43	1.85695	-0.00455	0.00000	-0.02416	-0.02416	1.83279
A44	1.85597	0.00108	0.00000	-0.00827	-0.00851	1.84745
A45	1.97488	-0.00281	0.00000	-0.01845	-0.01866	1.95623
A46	1.90339	0.00237	0.00000	0.01928	0.01937	1.92276
A47	1.98311	-0.00204	0.00000	-0.01457	-0.01479	1.96832
A48	1.85077	0.00112	0.00000	0.01242	0.01252	1.86329
A49	1.89424	0.00122	0.00000	-0.00351	-0.00395	1.89029
A50	1.84862	0.00058	0.00000	0.00866	0.00873	1.85735
A51	1.92894	0.00047	0.00000	0.00607	0.00610	1.93504
A52	1.93747	-0.00345	0.00000	-0.01780	-0.01796	1.91951
A53	1.95927	-0.00281	0.00000	-0.02019	-0.02038	1.93889
A54	1.85318	0.00311	0.00000	0.02647	0.02653	1.87971

A55	1.89389	0.00082	0.00000	0.00218	0.00219	1.89608
A56	1.88727	0.00231	0.00000	0.00619	0.00582	1.89308
A57	2.13785	-0.00604	0.00000	-0.02229	-0.02229	2.11556
A58	2.12411	0.00721	0.00000	0.02642	0.02641	2.15052
A59	2.01996	-0.00119	0.00000	-0.00445	-0.00446	2.01550
A60	2.18139	0.01193	0.00000	0.04912	0.04911	2.23049
A61	2.01616	-0.00668	0.00000	-0.02872	-0.02874	1.98742
A62	2.08447	-0.00526	0.00000	-0.02097	-0.02099	2.06348
A63	2.19767	-0.02368	0.00000	-0.08691	-0.08695	2.11072
A64	2.09196	0.02406	0.00000	0.08846	0.08843	2.18039
A65	1.99343	-0.00038	0.00000	-0.00133	-0.00136	1.99207
A66	1.94365	0.00204	0.00000	0.01706	0.01704	1.96068
A67	1.92863	-0.00059	0.00000	-0.00568	-0.00585	1.92278
A68	1.92733	-0.00176	0.00000	-0.01307	-0.01319	1.91413
A69	1.88165	0.00090	0.00000	0.01146	0.01139	1.89303
A70	1.88190	0.00108	0.00000	0.01068	0.01070	1.89260
A71	1.89912	-0.00164	0.00000	-0.02035	-0.02062	1.87850
A72	1.93016	0.00167	0.00000	0.00259	0.00257	1.93273
A73	1.92722	-0.00168	0.00000	-0.00399	-0.00404	1.92317
A74	1.96849	0.00033	0.00000	0.00316	0.00310	1.97159
A75	1.87622	-0.00077	0.00000	-0.00699	-0.00700	1.86922
A76	1.91753	-0.00334	0.00000	-0.02731	-0.02734	1.89019
A77	1.84012	0.00376	0.00000	0.03306	0.03297	1.87309
D1	1.00526	0.00115	0.00000	0.01167	0.01260	1.01785
D2	-3.11546	0.00221	0.00000	0.00872	0.00881	-3.10665
D3	-1.03583	-0.00416	0.00000	-0.04262	-0.04364	-1.07948
D4	1.12302	0.00069	0.00000	0.02998	0.02996	1.15298
D5	-0.98912	0.00184	0.00000	0.02232	0.02192	-0.96720
D6	-3.04838	-0.00062	0.00000	0.00259	0.00302	-3.04536
D7	2.90038	-0.00101	0.00000	0.01008	0.01014	2.91052
D8	-1.29922	0.00028	0.00000	0.01917	0.01925	-1.27997
D9	0.76966	-0.00072	0.00000	0.00775	0.00778	0.77745
D10	0.73857	-0.00423	0.00000	-0.03884	-0.03859	0.69998

D11	2.82216	-0.00294	0.00000	-0.02975	-0.02948	2.79267
D12	-1.39215	-0.00394	0.00000	-0.04117	-0.04095	-1.43310
D13	-1.29582	-0.00146	0.00000	-0.00644	-0.00652	-1.30233
D14	0.78777	-0.00016	0.00000	0.00265	0.00259	0.79036
D15	2.85665	-0.00116	0.00000	-0.00877	-0.00888	2.84778
D16	0.95861	0.00081	0.00000	0.02818	0.02837	0.98698
D17	-1.17364	0.00114	0.00000	-0.02344	-0.02554	-1.19918
D18	3.05015	0.00021	0.00000	-0.00831	-0.00914	3.04101
D19	3.13587	0.00115	0.00000	0.06270	0.06431	-3.08301
D20	1.00362	0.00148	0.00000	0.01108	0.01040	1.01402
D21	-1.05577	0.00055	0.00000	0.02620	0.02680	-1.02897
D22	-1.11581	-0.00081	0.00000	0.03100	0.03249	-1.08331
D23	3.03513	-0.00048	0.00000	-0.02063	-0.02142	3.01371
D24	0.97574	-0.00141	0.00000	-0.00550	-0.00501	0.97072
D25	-0.22875	0.00318	0.00000	0.02558	0.02525	-0.20350
D26	2.92375	0.00411	0.00000	0.02627	0.02589	2.94964
D27	-2.38237	-0.00846	0.00000	-0.07398	-0.07352	-2.45589
D28	0.77013	-0.00753	0.00000	-0.07330	-0.07288	0.69725
D29	1.83084	-0.00203	0.00000	-0.01798	-0.01770	1.81314
D30	-1.29985	-0.00110	0.00000	-0.01730	-0.01706	-1.31691
D31	1.16336	-0.00536	0.00000	-0.04363	-0.04385	1.11951
D32	-1.00261	-0.00106	0.00000	-0.00163	-0.00164	-1.00425
D33	-3.09511	0.00171	0.00000	0.02518	0.02520	-3.06991
D34	-0.93389	-0.00595	0.00000	-0.06198	-0.06210	-0.99599
D35	-3.09986	-0.00165	0.00000	-0.01997	-0.01989	-3.11975
D36	1.09082	0.00112	0.00000	0.00684	0.00695	1.09777
D37	-2.93963	-0.00573	0.00000	-0.05729	-0.05739	-2.99702
D38	1.17758	-0.00142	0.00000	-0.01528	-0.01517	1.16241
D39	-0.91492	0.00134	0.00000	0.01152	0.01167	-0.90325
D40	2.82543	-0.00573	0.00000	-0.04052	-0.04015	2.78528
D41	-1.41657	-0.00139	0.00000	0.01586	0.01606	-1.40051
D42	0.75844	-0.00013	0.00000	0.01030	0.01084	0.76928
D43	-1.31661	-0.00344	0.00000	-0.03211	-0.03213	-1.34873

D44	0.72458	0.00090	0.00000	0.02428	0.02408	0.74866
D45	2.89959	0.00216	0.00000	0.01871	0.01887	2.91846
D46	0.76854	-0.00343	0.00000	-0.02954	-0.03006	0.73848
D47	2.80973	0.00091	0.00000	0.02684	0.02615	2.83588
D48	-1.29845	0.00216	0.00000	0.02128	0.02094	-1.27751
D49	1.03923	-0.00282	0.00000	-0.01494	-0.01466	1.02457
D50	-1.13633	-0.00452	0.00000	-0.03757	-0.03738	-1.17372
D51	3.10053	-0.00293	0.00000	-0.01938	-0.01912	3.08141
D52	-3.11340	0.00583	0.00000	0.08787	0.08738	-3.02602
D53	0.99422	0.00414	0.00000	0.06525	0.06466	1.05888
D54	-1.05210	0.00573	0.00000	0.08343	0.08292	-0.96918
D55	-1.05351	0.00056	0.00000	0.02469	0.02501	-1.02850
D56	3.05411	-0.00113	0.00000	0.00206	0.00229	3.05641
D57	1.00779	0.00045	0.00000	0.02025	0.02055	1.02835
D58	-0.97709	0.00357	0.00000	0.05029	0.05010	-0.92700
D59	-3.03228	0.00233	0.00000	0.03331	0.03326	-2.99902
D60	1.19332	0.00126	0.00000	0.01844	0.01851	1.21183
D61	-3.12331	-0.00232	0.00000	-0.03517	-0.03501	3.12487
D62	1.10468	-0.00356	0.00000	-0.05216	-0.05184	1.05285
D63	-0.95290	-0.00463	0.00000	-0.06703	-0.06659	-1.01949
D64	1.09178	0.00219	0.00000	0.02943	0.02904	1.12082
D65	-0.96341	0.00095	0.00000	0.01245	0.01221	-0.95120
D66	-3.02100	-0.00012	0.00000	-0.00242	-0.00254	-3.02354
D67	1.84324	0.00029	0.00000	-0.01289	-0.01323	1.83002
D68	-1.29923	0.00068	0.00000	-0.00177	-0.00214	-1.30137
D69	-0.22194	-0.00183	0.00000	-0.03886	-0.03869	-0.26064
D70	2.91877	-0.00144	0.00000	-0.02774	-0.02761	2.89116
D71	-2.41116	-0.00245	0.00000	-0.02552	-0.02526	-2.43641
D72	0.72955	-0.00206	0.00000	-0.01440	-0.01417	0.71538
D73	-1.11267	0.00105	0.00000	0.01544	0.01522	-1.09746
D74	0.93887	0.00304	0.00000	0.04097	0.04062	0.97949
D75	3.05346	0.00160	0.00000	0.02222	0.02208	3.07555
D76	0.96212	-0.00065	0.00000	-0.00942	-0.00909	0.95303

D77	3.01367	0.00134	0.00000	0.01611	0.01631	3.02998
D78	-1.15493	-0.00010	0.00000	-0.00264	-0.00222	-1.15715
D79	-3.12424	-0.00277	0.00000	-0.03173	-0.03180	3.12715
D80	-1.07269	-0.00078	0.00000	-0.00620	-0.00639	-1.07909
D81	1.04190	-0.00222	0.00000	-0.02495	-0.02493	1.01697
D82	-0.03206	0.00034	0.00000	0.01614	0.01638	-0.01569
D83	3.11041	-0.00006	0.00000	0.00499	0.00496	3.11537
D84	3.09856	-0.00063	0.00000	0.01529	0.01557	3.11413
D85	-0.04214	-0.00102	0.00000	0.00414	0.00415	-0.03799
D86	0.53133	-0.00010	0.00000	0.00202	0.00183	0.53315
D87	-2.66349	-0.00011	0.00000	-0.00372	-0.00382	-2.66732
D88	2.72249	0.00174	0.00000	0.02513	0.02502	2.74751
D89	-0.47233	0.00173	0.00000	0.01939	0.01937	-0.45296
D90	-1.55721	-0.00116	0.00000	-0.00177	-0.00159	-1.55880
D91	1.53116	-0.00117	0.00000	-0.00750	-0.00725	1.52392
D92	-0.59094	0.00073	0.00000	0.02860	0.02854	-0.56240
D93	2.60307	0.00125	0.00000	0.04222	0.04215	2.64522
D94	2.60341	0.00118	0.00000	0.03590	0.03596	2.63938
D95	-0.48576	0.00170	0.00000	0.04951	0.04957	-0.43619
D96	-3.14158	0.00065	0.00000	0.01677	0.01674	-3.12484
D97	0.01848	0.00009	0.00000	0.00101	0.00104	0.01952
D98	-0.05431	0.00009	0.00000	0.00256	0.00253	-0.05178
D99	3.10575	-0.00047	0.00000	-0.01320	-0.01318	3.09257
D100	-0.01809	-0.00068	0.00000	-0.01352	-0.01315	-0.03123
D101	2.07274	0.00139	0.00000	0.00828	0.00861	2.08135
D102	-2.10837	-0.00220	0.00000	-0.02941	-0.02889	-2.13726
D103	3.10595	0.00010	0.00000	0.00237	0.00193	3.10788
D104	-1.08641	0.00217	0.00000	0.02418	0.02369	-1.06272
D105	1.01566	-0.00142	0.00000	-0.01352	-0.01381	1.00185
D106	1.46335	-0.00024	0.00000	0.00888	0.00931	1.47265
D107	-2.74539	-0.00120	0.00000	-0.00069	-0.00031	-2.74570
D108	-0.69168	0.00262	0.00000	0.04031	0.04074	-0.65094
D109	-1.66185	-0.00046	0.00000	-0.00409	-0.00449	-1.66634

D110 0.41260 -0.00142 0.00000 -0.01366 -0.01411 0.39849

D111 2.46631 0.00240 0.00000 0.02733 0.02694 2.49325

Item	Value	Threshold	Converged?
Maximum Force	0.024063	0.000450	NO
RMS Force	0.004325	0.000300	NO
Maximum Displacement	0.750821	0.001800	NO
RMS Displacement	0.190986	0.001200	NO

Predicted change in Energy=-1.307602D-02

GradGradGradGradGradGradGradGradGradGradGradGradGradGradGradGradGradGrad

Input orientation:

Center Number	Atomic Number	Atomic Type	Coordinates (Angstroms) X	Y	Z
1	8	0	1.542812	2.238655	-0.506246
2	8	0	4.031546	0.313255	1.336031
3	8	0	-2.351456	-0.968399	-1.495132
4	6	0	0.747320	-0.693386	-0.379051
5	6	0	0.959127	0.258250	0.821978
6	6	0	1.992604	1.362317	0.540413
7	6	0	-0.036403	-2.013257	-0.052159
8	6	0	3.364160	0.782820	0.140431
9	6	0	2.059037	-1.006572	-1.058405
10	6	0	3.212714	-0.367364	-0.827919
11	6	0	-1.428388	-1.770100	0.571200
12	6	0	0.742797	-2.993266	0.844034
13	6	0	4.278511	1.859179	-0.434024
14	6	0	-2.394104	-0.945921	-0.266119
15	6	0	-3.417625	-0.193560	0.496998
16	6	0	-4.022532	0.966266	0.152189
17	6	0	-5.028755	1.608732	1.057130
18	6	0	-3.772903	1.725992	-1.135998

19	1	0	0.153964	-0.144593	-1.120998
20	1	0	0.014099	0.750187	1.075032
21	1	0	1.276191	-0.285344	1.715981
22	1	0	2.146664	1.952265	1.453634
23	1	0	-0.199452	-2.529727	-1.005205
24	1	0	2.044651	-1.808198	-1.795285
25	1	0	4.115494	-0.679926	-1.349042
26	1	0	-1.346127	-1.333145	1.569000
27	1	0	-1.927262	-2.740933	0.708504
28	1	0	1.735016	-3.217589	0.442458
29	1	0	0.206393	-3.949427	0.915567
30	1	0	0.861225	-2.627895	1.868041
31	1	0	4.376920	2.700826	0.262490
32	1	0	5.279881	1.448306	-0.603564
33	1	0	3.893899	2.235413	-1.383598
34	1	0	0.699391	2.615092	-0.222839
35	1	0	3.531136	-0.434350	1.690809
36	1	0	-3.644532	-0.622899	1.471706
37	1	0	-5.211329	1.018906	1.962272
38	1	0	-4.685907	2.605862	1.355923
39	1	0	-5.979362	1.730171	0.524813
40	1	0	-4.389426	1.326094	-1.947615
41	1	0	-4.046917	2.780758	-1.012559
42	1	0	-2.728473	1.696404	-1.452377

Distance matrix (angstroms):

		1	2	3	4	5
1	O	0.000000				
2	O	3.646224	0.000000			
3	O	5.140857	7.099355	0.000000		
4	C	3.040699	3.839398	3.305099	0.000000	
5	C	2.454968	3.115612	4.222988	1.546915	0.000000
6	C	1.437281	2.427103	5.333530	2.573337	1.538284

7	C	4.558384	4.887529	2.921193	1.569439	2.629628
8	C	2.419701	1.447535	6.197588	3.049081	2.554186
9	C	3.332096	3.371359	4.432227	1.510036	2.519011
10	C	3.111813	2.411723	5.636169	2.527042	2.862203
11	C	5.104804	5.893744	2.400938	2.606913	3.142821
12	C	5.462259	4.689457	4.375635	2.604881	3.258779
13	C	2.762837	2.363043	7.285439	4.357514	3.893434
14	C	5.069367	6.741023	1.229957	3.153581	3.725338
15	C	5.614989	7.513387	2.388655	4.285330	4.411995
16	C	5.746786	8.166767	3.041232	5.078203	5.076103
17	C	6.784279	9.156698	4.508168	6.381647	6.142787
18	C	5.377381	8.307598	3.067447	5.182545	5.327293
19	H	2.826072	4.613270	2.663788	1.097146	2.141431
20	H	2.655737	4.049557	3.892960	2.176207	1.095041
21	H	3.373418	2.845112	4.892610	2.198946	1.093282
22	H	2.070697	2.500593	6.120342	3.509469	2.163082
23	H	5.101166	5.609392	2.703497	2.158840	3.528982
24	H	4.276737	4.272407	4.485656	2.220719	3.506942
25	H	3.980844	2.864101	6.475029	3.505091	3.944130
26	H	5.040870	5.628879	3.245401	2.930310	2.899099
27	H	6.189775	6.725269	2.859690	3.539578	4.164039
28	H	5.541442	4.305739	5.050976	2.832317	3.581550
29	H	6.488445	5.742739	4.608751	3.545482	4.275502
30	H	5.457573	4.357102	4.938236	2.967275	3.071427
31	H	2.972663	2.640506	7.862792	5.010614	4.237985
32	H	3.820970	2.570741	8.054357	5.018103	4.702906
33	H	2.509456	3.333170	7.020065	4.414515	4.169727
34	H	0.966118	4.339561	4.875223	3.312510	2.591101
35	H	3.990669	0.967052	6.711209	3.478657	2.801748
36	H	6.245744	7.734143	3.254772	4.766407	4.732044
37	H	7.293818	9.290902	4.907330	6.627159	6.320869
38	H	6.511486	9.013902	5.133567	6.589015	6.137001
39	H	7.609516	10.143176	4.952188	7.206862	7.099119

40	H	6.172662	9.095106	3.102058	5.738018	6.117022
41	H	5.638732	8.767327	4.142903	5.954471	5.898225
42	H	4.408296	7.442190	2.691681	4.352501	4.565017

		6	7	8	9	10
6	C	0.000000				
7	C	3.982777	0.000000			
8	C	1.541742	4.406696	0.000000		
9	C	2.858718	2.533143	2.518428	0.000000	
10	C	2.520476	3.723913	1.511145	1.338911	0.000000
11	C	4.638552	1.544449	5.447153	3.924375	5.046288
12	C	4.541509	1.539718	4.650314	3.049379	3.973824
13	C	2.534122	5.810342	1.524659	3.678104	2.499715
14	C	5.022117	2.596871	6.025896	4.523478	5.664517
15	C	5.629673	3.878857	6.860981	5.751008	6.763653
16	C	6.040648	4.980817	7.388978	6.507158	7.422126
17	C	7.044658	6.266810	8.483130	7.845515	8.682176
18	C	6.015287	5.396121	7.311396	6.440842	7.299034
19	H	2.900286	2.161149	3.571646	2.091943	3.080823
20	H	2.138925	2.984918	3.478139	3.437954	3.886036
21	H	2.147090	2.799090	2.825411	2.971571	3.198167
22	H	1.098064	4.770594	2.138785	3.882362	3.423832
23	H	4.726737	1.096185	4.998477	2.724628	4.043528
24	H	3.938320	2.722375	3.493061	1.088946	2.091930
25	H	3.499638	4.549498	2.218707	2.102424	1.088245
26	H	4.412554	2.192279	5.357698	4.313357	5.240323
27	H	5.677170	2.164132	6.382686	4.692606	5.866325
28	H	4.588191	2.198405	4.329966	2.691872	3.452714
29	H	5.616575	2.178118	5.741646	3.998656	4.990880
30	H	4.354814	2.206962	4.569715	3.553523	4.231749
31	H	2.748420	6.465215	2.172402	4.567510	3.458057
32	H	3.481705	6.367837	2.160182	4.075187	2.760466
33	H	2.842365	5.938956	2.171016	3.739376	2.747224
34	H	1.955607	4.689577	3.254256	3.957683	3.946892

35	H	2.630305	4.272964	1.978145	3.170599	2.539660
36	H	6.048610	4.156182	7.271183	6.251349	7.237084
37	H	7.350936	6.327069	8.770054	8.129284	8.981726
38	H	6.842073	6.703494	8.342930	8.023273	8.717651
39	H	7.980464	7.047334	9.399289	8.637835	9.524904
40	H	6.849957	5.804552	8.048179	6.914817	7.868548
41	H	6.395273	6.323700	7.761779	7.185308	7.914986
42	H	5.135309	4.792645	6.363321	5.511946	6.320348

		11	12	13	14	15
11	C	0.000000				
12	C	2.506914	0.000000			
13	C	6.837459	6.138479	0.000000		
14	C	1.520849	3.906944	7.240205	0.000000	
15	C	2.539300	5.026718	8.019417	1.481888	0.000000
16	C	3.793790	6.234172	8.369483	2.546216	1.352776
17	C	4.961378	7.384755	9.429288	3.901106	2.481481
18	C	4.542459	6.825208	8.083055	3.129999	2.545109
19	H	2.830131	3.510415	4.636693	2.804567	3.921296
20	H	2.947280	3.820707	4.657502	3.236497	3.605761
21	H	3.290862	2.894415	4.270296	4.223287	4.850387
22	H	5.236000	5.176943	2.848980	5.654698	6.039953
23	H	2.138310	2.126590	6.296101	2.805564	4.250991
24	H	4.202821	3.172572	4.504755	4.773302	6.139876
25	H	5.967448	4.640712	2.703866	6.604418	7.771248
26	H	1.092383	2.764992	6.770495	2.148455	2.595948
27	H	1.100111	2.685378	7.808843	2.095209	2.958889
28	H	3.481225	1.093655	5.745535	4.765730	5.974733
29	H	2.746009	1.098677	7.221043	4.144878	5.235956
30	H	2.767659	1.093668	6.091895	4.240382	5.110215
31	H	7.333899	6.779950	1.096898	7.708758	8.317896
32	H	7.532535	6.512160	1.095583	8.045883	8.919281
33	H	6.942053	6.498528	1.091407	7.135028	7.930633
34	H	4.938405	5.709096	3.664165	4.717244	5.035524

35	H	5.256866	3.878133	3.214617	6.260970	7.054676
36	H	2.652974	5.026059	8.518634	2.165167	1.088978
37	H	4.901456	7.266333	9.823714	4.094267	2.614282
38	H	5.511466	7.815567	9.171817	4.527531	3.191090
39	H	5.741551	8.221933	10.303397	4.543250	3.203750
40	H	4.969767	7.265665	8.815230	3.459883	3.038072
41	H	5.484095	7.728367	8.396235	4.144527	3.394310
42	H	4.219210	6.270265	7.082469	2.915629	2.801243

		16	17	18	19	20
16	C	0.000000				
17	C	1.498055	0.000000			
18	C	1.516220	2.529966	0.000000		
19	H	4.505345	5.888886	4.349667	0.000000	
20	H	4.146410	5.115448	4.492465	2.375446	0.000000
21	H	5.664666	6.616189	6.137802	3.054121	1.753858
22	H	6.381609	7.194573	6.465189	3.872518	2.477128
23	H	5.308245	6.685974	5.558579	2.413954	3.889836
24	H	6.949893	8.357318	6.838793	2.607095	4.348241
25	H	8.437482	9.728562	8.249887	4.004036	4.974214
26	H	3.802339	4.741139	4.750218	3.301366	2.536630
27	H	4.294528	5.353540	5.173200	3.797310	4.011377
28	H	7.123079	8.331854	7.567542	3.793076	4.370921
29	H	6.529210	7.636750	7.228699	4.315914	4.706249
30	H	6.301842	7.300579	7.032476	3.949856	3.571823
31	H	8.577392	9.502150	8.326204	5.276723	4.847620
32	H	9.345502	10.442779	9.072678	5.392596	5.570773
33	H	8.163287	9.271659	7.687696	4.440777	4.827381
34	H	5.015558	5.955060	4.650351	2.952971	2.373175
35	H	7.834984	8.823122	8.124463	4.404033	3.761895
36	H	2.099870	2.658594	3.511964	4.623793	3.927887
37	H	2.166197	1.095677	3.488311	6.296559	5.307024
38	H	2.139466	1.095942	2.795964	6.092987	5.060878
39	H	2.133445	1.096249	2.761661	6.621263	6.097925

40	H	2.161774	3.084983	1.094871	4.846505	5.372067
41	H	2.156297	2.573182	1.096746	5.120239	4.997311
42	H	2.186852	3.405380	1.091698	3.436208	3.847705
		21	22	23	24	25
21	H	0.000000				
22	H	2.415253	0.000000			
23	H	3.823562	5.624802	0.000000		
24	H	3.903666	4.970610	2.486127	0.000000	
25	H	4.196630	4.319690	4.707307	2.400109	0.000000
26	H	2.827728	4.796543	3.061569	4.800153	6.226634
27	H	4.160173	6.259250	2.442690	4.786966	6.707916
28	H	3.229620	5.283875	2.512179	2.662659	3.913551
29	H	3.900082	6.235715	2.422731	3.913154	5.576663
30	H	2.383876	4.775140	3.064345	3.936033	4.973377
31	H	4.543607	2.636893	7.064624	5.477702	3.754313
32	H	4.941191	3.781942	6.783004	4.742545	2.537895
33	H	4.776400	3.344082	6.293271	4.465422	2.923953
34	H	3.536135	2.311817	5.281019	4.883421	4.878037
35	H	2.260003	2.769286	5.057310	4.031123	3.105234
36	H	4.938333	6.337961	4.651844	6.666706	8.256989
37	H	6.621906	7.434375	6.820383	8.646444	10.041935
38	H	6.635912	6.864456	7.216472	8.643752	9.776395
39	H	7.623926	8.181952	7.341325	9.071257	10.546375
40	H	6.936706	7.394665	5.771601	7.158515	8.758772
41	H	6.721711	6.717809	6.557766	7.666706	8.872120
42	H	5.477514	5.681315	4.945310	5.931487	7.245517
		26	27	28	29	30
26	H	0.000000				
27	H	1.749297	0.000000			
28	H	3.783341	3.702736	0.000000		
29	H	3.111627	2.460856	1.759576	0.000000	
30	H	2.576472	3.022079	1.773002	1.755693	0.000000
31	H	7.122721	8.339927	6.483802	7.876908	6.582794

32	H	7.507362	8.438848	5.952379	7.561982	6.499905
33	H	6.993591	7.938943	6.142515	7.558843	6.589539
34	H	4.794100	6.037690	5.961150	6.680712	5.646846
35	H	4.960884	6.006610	3.539895	4.900071	3.459983
36	H	2.407608	2.831530	6.060635	5.119052	4.947619
37	H	4.541650	5.147176	8.277042	7.425067	7.084062
38	H	5.168685	6.051239	8.716378	8.191478	7.643634
39	H	5.651645	6.036889	9.165083	8.406788	8.221352
40	H	5.357219	5.445909	7.991639	7.559794	7.600170
41	H	5.557240	6.159876	8.457422	8.191685	7.851194
42	H	4.496417	5.000131	6.903656	6.789404	6.527685

		31	32	33	34	35
31	H	0.000000				
32	H	1.770366	0.000000			
33	H	1.777505	1.774524	0.000000		
34	H	3.710406	4.742070	3.420001	0.000000	
35	H	3.547503	3.444800	4.087934	4.580385	0.000000
36	H	8.766586	9.393709	8.552815	5.676770	7.181488
37	H	9.881934	10.808949	9.776498	6.500701	8.866585
38	H	9.129044	10.222350	9.014171	5.611951	8.767829
39	H	10.404978	11.319154	10.068694	6.778481	9.823150
40	H	9.144576	9.763038	8.352153	5.525615	8.892278
41	H	8.520162	9.430370	7.968164	4.814410	8.664411
42	H	7.378092	8.057033	6.644628	3.755796	7.321369

		36	37	38	39	40
36	H	0.000000				
37	H	2.321859	0.000000			
38	H	3.394519	1.778245	0.000000		
39	H	3.447459	1.778218	1.769352	0.000000	
40	H	4.005645	4.007131	3.555147	2.967166	0.000000
41	H	4.233009	3.648232	2.459391	2.683578	1.762864
42	H	3.842993	4.275910	3.541922	3.805089	1.772330

		41	42

41 H 0.000000

42 H 1.762827 0.000000

Stoichiometry C15H24O3

Framework group C1[X(C15H24O3)]

Deg. of freedom 120

Full point group C1 NOp 1

Largest Abelian subgroup C1 NOp 1

Largest concise Abelian subgroup C1 NOp 1

Standard orientation:

Center Number	Atomic Number	Atomic Type	Coordinates (Angstroms) X	Y	Z
1	8	0	1.502201	-2.219082	0.390670
2	8	0	3.997729	-0.178659	-1.313433
3	8	0	-2.398660	0.916469	1.565412
4	6	0	0.705226	0.714756	0.447877
5	6	0	0.923023	-0.157953	-0.810650
6	6	0	1.956015	-1.277351	-0.595723
7	6	0	-0.077965	2.052521	0.202647
8	6	0	3.325377	-0.724078	-0.153338
9	6	0	2.013698	0.984354	1.151742
10	6	0	3.168833	0.361692	0.885967
11	6	0	-1.467002	1.849190	-0.441214
12	6	0	0.704514	3.088215	-0.625481
13	6	0	4.237927	-1.834625	0.355115
14	6	0	-2.435842	0.972697	0.337303
15	6	0	-3.455443	0.270308	-0.476979
16	6	0	-4.061059	-0.909465	-0.209830
17	6	0	-5.062810	-1.493099	-1.158531
18	6	0	-3.816612	-1.749995	1.028184
19	1	0	0.108971	0.119344	1.150511
20	1	0	-0.020529	-0.633073	-1.098898

21	1	0	1.243667	0.441890	-1.666595
22	1	0	2.114536	-1.807562	-1.544139
23	1	0	-0.245599	2.506851	1.186062
24	1	0	1.995484	1.737157	1.938355
25	1	0	4.069075	0.640627	1.430061
26	1	0	-1.380011	1.477043	-1.464561
27	1	0	-1.965942	2.826614	-0.518316
28	1	0	1.694785	3.286783	-0.205937
29	1	0	0.167762	4.046772	-0.638055
30	1	0	0.827737	2.789198	-1.670237
31	1	0	4.340012	-2.629915	-0.393401
32	1	0	5.238247	-1.435032	0.555084
33	1	0	3.849373	-2.271036	1.276929
34	1	0	0.660308	-2.576954	0.079979
35	1	0	3.498373	0.589916	-1.621858
36	1	0	-3.678326	0.761070	-1.423207
37	1	0	-5.241782	-0.846613	-2.024865
38	1	0	-4.717941	-2.468912	-1.519007
39	1	0	-6.015683	-1.648761	-0.639334
40	1	0	-4.437008	-1.403131	1.860972
41	1	0	-4.089337	-2.794810	0.836254
42	1	0	-2.773616	-1.740288	1.350473

Rotational constants (GHZ): 0.6601111 0.2162124 0.1858571

Standard basis: 6-31+G(2d,p) (6D, 7F)

There are 570 symmetry adapted cartesian basis functions of A symmetry.

There are 570 symmetry adapted basis functions of A symmetry.

570 basis functions, 852 primitive gaussians, 570 cartesian basis functions

69 alpha electrons 69 beta electrons

nuclear repulsion energy 1387.6555956329 Hartrees.

NAtoms= 42 NActive= 42 NUniq= 42 SFac= 1.00D+00 NAtFMM= 60 NAOKFM=F Big=F

Integral buffers will be 262144 words long.

Raffenetti 2 integral format.

Two-electron integral symmetry is turned on.

Polarizable Continuum Model (PCM)

=================================

Model : PCM (using non-symmetric T matrix).

Atomic radii : SMD-Coulomb.

Polarization charges : Total charges.

Charge compensation : None.

Solution method : On-the-fly selection.

Cavity type : VdW (van der Waals Surface) (Alpha=1.000).

Cavity algorithm : GePol (No added spheres)

Default sphere list used, NSphG= 42.

Lebedev-Laikov grids with approx. 5.0 points / Ang**2.

Smoothing algorithm: Karplus/York (Gamma=1.0000).

Polarization charges: spherical gaussians, with

point-specific exponents (IZeta= 3).

Self-potential: point-specific (ISelfS= 7).

Self-field : sphere-specific E.n sum rule (ISelfD= 2).

1st derivatives : Analytical E(r).r(x)/FMM algorithm (CHGder, D1EAlg=3).

Cavity 1st derivative terms included.

Solvent : Water, Eps= 78.355300 Eps(inf)= 1.777849

Atomic radii for non-electrostatic terms: SMD-CDS.

Nuclear repulsion after PCM non-electrostatic terms = 1387.6671777030 Hartrees.

One-electron integrals computed using PRISM.

NBasis= 570 RedAO= T EigKep= 2.44D-06 NBF= 570

NBsUse= 570 1.00D-06 EigRej= -1.00D+00 NBFU= 570

Initial guess from the checkpoint file: "E:\Project Docking Bisacurone\working files\Bisacurone_op.chk"

B after Tr= 0.000000 0.000000 0.000000

Rot= 0.999869 -0.013242 -0.000233 -0.009345 Ang= -1.86 deg.

ExpMin= 4.38D-02 ExpMax= 5.48D+03 ExpMxC= 8.25D+02 IAcc=2 IRadAn= 4 AccDes= 0.00D+00

Harris functional with IExCor= 402 and IRadAn= 4 diagonalized for initial guess.

HarFok: IExCor= 402 AccDes= 0.00D+00 IRadAn= 4 IDoV= 1 UseB2=F ITyADJ=14

ICtDFT= 3500011 ScaDFX= 1.000000 1.000000 1.000000 1.000000

FoFCou: FMM=F IPFlag= 0 FMFlag= 100000 FMFlg1= 0

NFxFlg= 0 DoJE=T BraDBF=F KetDBF=T FulRan=T

wScrn= 0.000000 ICntrl= 500 IOpCl= 0 I1Cent= 200000004 NGrid= 0

NMat0= 1 NMatS0= 1 NMatT0= 0 NMatD0= 1 NMtDS0= 0 NMtDT0= 0

Petite list used in FoFCou.

Requested convergence on RMS density matrix=1.00D-08 within 128 cycles.

Requested convergence on MAX density matrix=1.00D-06.

Requested convergence on energy=1.00D-06.

No special actions if energy rises.

Inv3: Mode=1 IEnd= 23135187.

Iteration 1 A*A^-1 deviation from unit magnitude is 2.44D-15 for 280.

Iteration 1 A*A^-1 deviation from orthogonality is 2.92D-15 for 1907 1270.

Iteration 1 A^-1*A deviation from unit magnitude is 2.44D-15 for 206.

Iteration 1 A^-1*A deviation from orthogonality is 3.22D-13 for 2674 2670.

Error on total polarization charges = 0.08137

SCF Done: E(RB3LYP) = -811.792048749 A.U. after 13 cycles

NFock= 13 Conv=0.44D-08 -V/T= 2.0082

SMD-CDS (non-electrostatic) energy (kcal/mol) = 7.27

(included in total energy above)

S2: Supplementary LOG File of Molecular Orbital analysis by DFT theory of Bisacurone.

Entering Link 1 = C:\G09W\l1.exe PID= 18936.

Copyright (c) 1988,1990,1992,1993,1995,1998,2003,2009,2013,

Gaussian, Inc. All Rights Reserved.

This is part of the Gaussian(R) 09 program. It is based on

the Gaussian(R) 03 system (copyright 2003, Gaussian, Inc.),

the Gaussian(R) 98 system (copyright 1998, Gaussian, Inc.),

the Gaussian(R) 94 system (copyright 1995, Gaussian, Inc.),

the Gaussian 92(TM) system (copyright 1992, Gaussian, Inc.),

the Gaussian 90(TM) system (copyright 1990, Gaussian, Inc.),

the Gaussian 88(TM) system (copyright 1988, Gaussian, Inc.),

the Gaussian 86(TM) system (copyright 1986, Carnegie Mellon

University), and the Gaussian 82(TM) system (copyright 1983,

Carnegie Mellon University). Gaussian is a federally registered

trademark of Gaussian, Inc.

This software contains proprietary and confidential information,

including trade secrets, belonging to Gaussian, Inc.

This software is provided under written license and may be

used, copied, transmitted, or stored only in accord with that

written license.

The following legend is applicable only to US Government

contracts under FAR:

RESTRICTED RIGHTS LEGEND

Use, reproduction and disclosure by the US Government is

subject to restrictions as set forth in subparagraphs (a)

and (c) of the Commercial Computer Software - Restricted

Rights clause in FAR 52.227-19.

Gaussian, Inc.

340 Quinnipiac St., Bldg. 40, Wallingford CT 06492

Warning -- This program may not be used in any manner that competes with the business of Gaussian, Inc. or will provide assistance to any competitor of Gaussian, Inc. The licensee of this program is prohibited from giving any competitor of Gaussian, Inc. access to this program. By using this program, the user acknowledges that Gaussian, Inc. is engaged in the business of creating and licensing software in the field of computational chemistry and represents and warrants to the licensee that it is not a competitor of Gaussian, Inc. and that it will not use this program in any manner prohibited above.

Cite this work as:

Gaussian 09, Revision D.01,

M. J. Frisch, G. W. Trucks, H. B. Schlegel, G. E. Scuseria,

M. A. Robb, J. R. Cheeseman, G. Scalmani, V. Barone, B. Mennucci,

G. A. Petersson, H. Nakatsuji, M. Caricato, X. Li, H. P. Hratchian,

A. F. Izmaylov, J. Bloino, G. Zheng, J. L. Sonnenberg, M. Hada,

M. Ehara, K. Toyota, R. Fukuda, J. Hasegawa, M. Ishida, T. Nakajima,

Y. Honda, O. Kitao, H. Nakai, T. Vreven, J. A. Montgomery, Jr.,

J. E. Peralta, F. Ogliaro, M. Bearpark, J. J. Heyd, E. Brothers,

K. N. Kudin, V. N. Staroverov, T. Keith, R. Kobayashi, J. Normand,

K. Raghavachari, A. Rendell, J. C. Burant, S. S. Iyengar, J. Tomasi,

M. Cossi, N. Rega, J. M. Millam, M. Klene, J. E. Knox, J. B. Cross,

V. Bakken, C. Adamo, J. Jaramillo, R. Gomperts, R. E. Stratmann,

O. Yazyev, A. J. Austin, R. Cammi, C. Pomelli, J. W. Ochterski,

R. L. Martin, K. Morokuma, V. G. Zakrzewski, G. A. Voth,

P. Salvador, J. J. Dannenberg, S. Dapprich, A. D. Daniels,

O. Farkas, J. B. Foresman, J. V. Ortiz, J. Cioslowski,

and D. J. Fox, Gaussian, Inc., Wallingford CT, 2013.

****

Gaussian 09: IA32W-G09RevD.01 24-Apr-2013

19-Dec-2025

****

%chk=E:\Project Docking Bisacurone\working files\Bisacurone_op_mo.chk

----

b3lyp/6-311+g(2d,p) scrf=(smd,solvent=water,read) geom=connectivity

----

1/38=1,40=2,57=2/1;

2/12=2,17=6,18=5,40=1/2;

3/5=4,6=6,7=112,11=2,16=1,25=1,30=1,70=32203,72=1,74=-5/1,2,3;

4//1;

5/5=2,38=5,53=1/2;

6/7=2,8=2,9=2,10=2,28=1/1;

99/5=1,9=1/99;

--

MO

--

Symbolic Z-matrix:

Charge = 0 Multiplicity = 1

O 1.2533 2.2138 -0.3192

O 3.8828 0.2164 1.27

O -2.3795 -1.0169 -1.4099

C 0.6576 -0.7273 -0.4434

C 0.8391 0.1282 0.8257

C 1.818 1.2879 0.6076

C -0.0393 -2.0427 -0.0909

C	3.2036	0.7794	0.1405
C	1.9625	-0.9862	-1.1503
C	3.0986	-0.3283	-0.8781
C	-1.3685	-1.7944	0.6382
C	0.8815	-2.9023	0.7768
C	4.0725	1.919	-0.4031
C	-2.3517	-0.977	-0.183
C	-3.3342	-0.1714	0.5972
C	-3.8617	0.9991	0.1976
C	-4.8526	1.828	0.9663
C	-3.4821	1.5745	-1.1419
H	0.0386	-0.1641	-1.1528
H	-0.1293	0.5566	1.1118
H	1.1927	-0.4515	1.6819
H	1.9357	1.8227	1.5587
H	-0.2438	-2.5953	-1.0183
H	1.9762	-1.743	-1.9308
H	3.9987	-0.6057	-1.4215
H	-1.2124	-1.323	1.6144
H	-1.8555	-2.7585	0.8351
H	1.8528	-3.0958	0.3125
H	0.4168	-3.8846	0.9283
H	1.0496	-2.4928	1.7763
H	4.1664	2.7189	0.3405
H	5.0923	1.572	-0.6091
H	3.6655	2.3474	-1.3251
H	0.401	2.5108	0.0425
H	3.3881	-0.5623	1.5764
H	-3.5867	-0.5635	1.5786
H	-5.1223	1.3502	1.9139
H	-4.4328	2.8128	1.1954
H	-5.7707	1.9621	0.3854
H	-4.0954	1.1376	-1.9363

H	-3.6496	2.6579	-1.1542
H	-2.4212	1.4412	-1.3752

Input orientation:

Center Number	Atomic Number	Atomic Type	Coordinates (Angstroms) X	Y	Z
1	8	0	1.253300	2.213800	-0.319200
2	8	0	3.882800	0.216400	1.270000
3	8	0	-2.379500	-1.016900	-1.409900
4	6	0	0.657600	-0.727300	-0.443400
5	6	0	0.839100	0.128200	0.825700
6	6	0	1.818000	1.287900	0.607600
7	6	0	-0.039300	-2.042700	-0.090900
8	6	0	3.203600	0.779400	0.140500
9	6	0	1.962500	-0.986200	-1.150300
10	6	0	3.098600	-0.328300	-0.878100
11	6	0	-1.368500	-1.794400	0.638200
12	6	0	0.881500	-2.902300	0.776800
13	6	0	4.072500	1.919000	-0.403100
14	6	0	-2.351700	-0.977000	-0.183000
15	6	0	-3.334200	-0.171400	0.597200
16	6	0	-3.861700	0.999100	0.197600
17	6	0	-4.852600	1.828000	0.966300
18	6	0	-3.482100	1.574500	-1.141900
19	1	0	0.038600	-0.164100	-1.152800
20	1	0	-0.129300	0.556600	1.111800
21	1	0	1.192700	-0.451500	1.681900
22	1	0	1.935700	1.822700	1.558700
23	1	0	-0.243800	-2.595300	-1.018300
24	1	0	1.976200	-1.743000	-1.930800
25	1	0	3.998700	-0.605700	-1.421500

26	1	0	-1.212400	-1.323000	1.614400
27	1	0	-1.855500	-2.758500	0.835100
28	1	0	1.852800	-3.095800	0.312500
29	1	0	0.416800	-3.884600	0.928300
30	1	0	1.049600	-2.492800	1.776300
31	1	0	4.166400	2.718900	0.340500
32	1	0	5.092300	1.572000	-0.609100
33	1	0	3.665500	2.347400	-1.325100
34	1	0	0.401000	2.510800	0.042500
35	1	0	3.388100	-0.562300	1.576400
36	1	0	-3.586700	-0.563500	1.578600
37	1	0	-5.122300	1.350200	1.913900
38	1	0	-4.432800	2.812800	1.195400
39	1	0	-5.770700	1.962100	0.385400
40	1	0	-4.095400	1.137600	-1.936300
41	1	0	-3.649600	2.657900	-1.154200
42	1	0	-2.421200	1.441200	-1.375200

Distance matrix (angstroms):

		1	2	3	4	5
1	O	0.000000				
2	O	3.664619	0.000000			
3	O	4.982397	6.922376	0.000000		
4	C	3.003390	3.772032	3.200307	0.000000	
5	C	2.414971	3.077221	4.082713	1.541245	0.000000
6	C	1.426582	2.418736	5.196289	2.551893	1.533205
7	C	4.454293	4.726357	2.875511	1.529772	2.514866
8	C	2.464244	1.433196	6.066419	3.015493	2.546453
9	C	3.381374	3.315366	4.349862	1.506486	2.531500
10	C	3.190576	2.350745	5.546761	2.511304	2.866473
11	C	4.884272	5.658502	2.412746	2.532517	2.933436
12	C	5.245372	4.356299	4.355515	2.503926	3.031191
13	C	2.835813	2.394597	7.159711	4.320425	3.895098

14	C	4.816200	6.511866	1.227863	3.030849	3.524223
15	C	5.251107	7.258659	2.377977	4.162492	4.190275
16	C	5.282594	7.857477	2.974093	4.880104	4.821877
17	C	6.251669	8.888009	4.456008	6.235312	5.941762
18	C	4.848665	7.867875	2.828941	4.787828	4.963467
19	H	2.797283	4.559892	2.576931	1.097089	2.154229
20	H	2.589528	4.029604	3.728039	2.164777	1.096895
21	H	3.333451	2.802212	4.758100	2.208913	1.092778
22	H	2.035961	2.540619	5.957925	3.484888	2.147359
23	H	5.085026	5.492795	2.684383	2.152314	3.462724
24	H	4.333140	4.209453	4.446423	2.232199	3.520324
25	H	4.086792	2.816639	6.391452	3.483449	3.946085
26	H	4.725192	5.333800	3.256104	2.843642	2.633758
27	H	5.976689	6.478214	2.889251	3.475055	3.948923
28	H	5.380548	4.001047	5.020045	2.758565	3.418353
29	H	6.280643	5.380345	4.637906	3.450810	4.036264
30	H	5.156035	3.952610	4.908042	2.863166	2.795996
31	H	3.029271	2.684568	7.737500	4.980208	4.244770
32	H	3.903059	2.613725	7.948049	4.998080	4.715180
33	H	2.616943	3.364955	6.918651	4.390745	4.187998
34	H	0.972343	4.346718	4.720732	3.284392	2.546000
35	H	3.982135	0.972102	6.510749	3.400360	2.745495
36	H	5.894119	7.516443	3.254848	4.704189	4.542358
37	H	6.810346	9.099007	4.916682	6.578762	6.181889
38	H	5.914774	8.711835	5.066580	6.413279	5.927621
39	H	7.063738	9.849876	4.857755	7.017323	6.873610
40	H	5.690502	8.647580	2.804154	5.319550	5.744286
41	H	4.993283	8.280985	3.896497	5.524200	5.519766
42	H	3.900512	6.945332	2.458699	3.879388	4.146985

		6	7	8	9	10
6	C	0.000000				
7	C	3.876901	0.000000			
8	C	1.548109	4.305136	0.000000		

9	C	2.877955	2.499145	2.514724	0.000000	
10	C	2.541524	3.661320	1.508499	1.340764	0.000000
11	C	4.433429	1.536233	5.270318	3.866197	4.940000
12	C	4.296910	1.529604	4.399084	2.924673	3.778849
13	C	2.550014	5.718342	1.532702	3.667505	2.494887
14	C	4.810532	2.547821	5.835319	4.421321	5.532608
15	C	5.354888	3.851183	6.622343	5.636727	6.601670
16	C	5.701798	4.893518	7.068946	6.299171	7.166931
17	C	6.702035	6.266406	8.166019	7.671069	8.442335
18	C	5.588734	5.103098	6.853854	6.016720	6.855352
19	H	2.893713	2.159361	3.546836	2.092187	3.076690
20	H	2.140326	2.865475	3.478690	3.445716	3.893853
21	H	2.137904	2.681895	2.816868	2.983262	3.193937
22	H	1.097477	4.643611	2.169640	3.902476	3.452120
23	H	4.687623	1.098753	4.961450	2.733932	4.041112
24	H	3.956622	2.745413	3.487018	1.087251	2.090295
25	H	3.529649	4.487862	2.233949	2.089124	1.087389
26	H	4.124775	2.191388	5.108182	4.223386	5.078061
27	H	5.469891	2.160655	6.212383	4.654029	5.777892
28	H	4.393759	2.202679	4.107483	2.569481	3.260154
29	H	5.368516	2.153925	5.489971	3.887221	4.806496
30	H	4.031128	2.207879	4.245333	3.415877	3.991153
31	H	2.762985	6.367641	2.174545	4.561516	3.451175
32	H	3.504584	6.298246	2.181124	4.078349	2.767371
33	H	2.875957	5.875523	2.195440	3.747486	2.771381
34	H	1.955176	4.576682	3.295742	4.011242	4.023063
35	H	2.612858	4.088832	1.973831	3.105949	2.482567
36	H	5.794938	4.190383	7.069631	6.198322	7.126284
37	H	7.062440	6.431787	8.531786	8.064892	8.842840
38	H	6.460908	6.673327	7.972586	7.799652	8.419495
39	H	7.621830	7.008155	9.055209	8.417436	9.246991
40	H	6.439124	5.474647	7.597158	6.467339	7.417701
41	H	5.905547	6.021670	7.222974	6.691423	7.384567

42	H	4.682501	4.411398	5.862909	5.015942	5.817769

		11	12	13	14	15
11	C	0.000000				
12	C	2.511803	0.000000			
13	C	6.669192	5.900812	0.000000		
14	C	1.519603	3.883501	7.050220	0.000000	
15	C	2.549468	5.026151	7.760772	1.490976	0.000000
16	C	3.770122	6.168820	8.009906	2.515935	1.344621
17	C	5.036704	7.435829	9.030003	3.929811	2.537592
18	C	4.357241	6.539431	7.598453	2.950838	2.468706
19	H	2.800980	3.454211	4.601489	2.704598	3.799781
20	H	2.699466	3.619106	4.669709	2.994578	3.326587
21	H	3.074481	2.631059	4.273142	4.039403	4.663459
22	H	4.984824	4.903910	2.902386	5.408663	5.716009
23	H	2.156475	2.140779	6.275972	2.785653	4.246846
24	H	4.217751	3.142203	4.487603	4.729934	6.087779
25	H	5.870452	4.452396	2.723361	6.480689	7.618083
26	H	1.095240	2.753213	6.520049	2.156007	2.619716
27	H	1.097920	2.741395	7.652015	2.111039	2.989354
28	H	3.489484	1.093819	5.530585	4.734200	5.961387
29	H	2.764126	1.097183	6.987027	4.165779	5.288429
30	H	2.762289	1.093137	5.775092	4.207772	5.098713
31	H	7.147980	6.525244	1.096174	7.511282	8.042309
32	H	7.391234	6.298485	1.096739	7.879854	8.689103
33	H	6.808099	6.303014	1.095107	6.968696	7.683449
34	H	4.692626	5.483770	3.745490	4.448928	4.631804
35	H	5.002353	3.521080	3.247102	6.017705	6.804480
36	H	2.705527	5.106631	8.291760	2.190763	1.086575
37	H	5.060329	7.444620	9.499283	4.181994	2.691889
38	H	5.561178	7.815330	8.700242	4.538011	3.235771
39	H	5.792630	8.250294	9.874825	4.544331	3.245491
40	H	4.760321	6.960692	8.347208	3.253626	2.951530
41	H	5.314047	7.428018	7.793648	3.979984	3.342427

42	H	3.953614	5.865571	6.583419	2.697010	2.706365
		16	17	18	19	20
16	C	0.000000				
17	C	1.503282	0.000000			
18	C	1.506467	2.527259	0.000000		
19	H	4.288234	5.690596	3.926599	0.000000	
20	H	3.868123	4.893587	4.166120	2.382438	0.000000
21	H	5.463913	6.500297	5.825142	3.074097	1.757545
22	H	6.011718	6.814102	6.058663	3.859870	2.463119
23	H	5.242836	6.689190	5.281013	2.451239	3.805905
24	H	6.791927	8.232724	6.435931	2.617729	4.356456
25	H	8.184299	9.485252	7.797039	3.993695	4.980857
26	H	3.797139	4.857972	4.598286	3.250453	2.226793
27	H	4.307064	5.480493	5.032817	3.777601	3.747828
28	H	7.031139	8.344678	7.237965	3.746103	4.231740
29	H	6.533759	7.771861	7.020607	4.279735	4.478410
30	H	6.229489	7.359441	6.752416	3.876157	3.336196
31	H	8.211487	9.084475	7.874434	5.251701	4.870676
32	H	9.008501	10.072162	8.590938	5.371176	5.590853
33	H	7.797132	8.836194	7.191601	4.414943	4.852417
34	H	4.525473	5.377726	4.166285	2.952146	2.289873
35	H	7.543120	8.602028	7.691210	4.338922	3.720201
36	H	2.103451	2.774290	3.461662	4.556629	3.664170
37	H	2.158256	1.094978	3.475411	6.191336	5.118908
38	H	2.147386	1.094783	2.810727	5.862542	4.859788
39	H	2.146373	1.094685	2.778591	6.374539	5.859051
40	H	2.151122	3.078164	1.094573	4.404345	5.035715
41	H	2.150341	2.575357	1.096341	4.643975	4.684307
42	H	2.178117	3.397638	1.094398	2.945686	3.495782
		21	22	23	24	25
21	H	0.000000				
22	H	2.395665	0.000000			
23	H	3.735036	5.559665	0.000000		

24	H	3.915794	4.989235	2.547052	0.000000	
25	H	4.186706	4.362876	4.703178	2.375572	0.000000
26	H	2.559018	4.450738	3.080267	4.786649	6.073448
27	H	3.915462	5.990341	2.461565	4.833567	6.633133
28	H	3.050133	5.074596	2.533231	2.622535	3.716482
29	H	3.599460	5.939506	2.426507	3.897773	5.394697
30	H	2.048486	4.410903	3.081100	3.894018	4.741757
31	H	4.549031	2.695034	7.038247	5.464827	3.766395
32	H	4.954807	3.837490	6.782905	4.737742	2.568722
33	H	4.794855	3.403501	6.309282	4.466764	2.973401
34	H	3.477016	2.264431	5.254838	4.946714	4.979891
35	H	2.200724	2.792491	4.904718	3.960804	3.059758
36	H	4.781828	6.015916	4.695432	6.682287	8.157247
37	H	6.571086	7.082710	6.925643	8.645132	9.906719
38	H	6.522161	6.455236	7.189975	8.461898	9.467022
39	H	7.483002	7.796452	7.299787	8.894212	10.261562
40	H	6.601561	7.004181	5.441699	6.720284	8.295696
41	H	6.415583	6.265218	6.262112	7.184751	8.319797
42	H	5.097887	5.266487	4.600194	5.457559	6.738476

		26	27	28	29	30
26	H	0.000000				
27	H	1.755433	0.000000			
28	H	3.772693	3.760103	0.000000		
29	H	3.112366	2.537742	1.750289	0.000000	
30	H	2.551722	3.065299	1.775232	1.748327	0.000000
31	H	6.847720	8.155355	6.258136	7.616510	6.240014
32	H	7.285206	8.313295	5.756046	7.348359	6.209360
33	H	6.775427	7.824201	5.966242	7.380358	6.315786
34	H	4.446566	5.786668	5.797809	6.456472	5.335050
35	H	4.663122	5.733076	3.220747	4.504035	3.038976
36	H	2.493075	2.892727	6.132187	5.242195	5.025591
37	H	4.745842	5.358844	8.425166	7.684801	7.271859
38	H	5.258458	6.149120	8.671784	8.273157	7.651375

39 H 5.751558 6.149397 9.149067 8.530170 8.264214

40 H 5.193622 5.279906 7.639363 7.334056 7.309872

41 H 5.427030 6.042640 8.095226 7.979770 7.563070

42 H 4.247318 4.779427 6.457536 6.459451 6.120029

31 32 33 34 35

31 H 0.000000

32 H 1.753400 0.000000

33 H 1.778521 1.774728 0.000000

34 H 3.782902 4.828480 3.543161 0.000000

35 H 3.591584 3.497991 4.118496 4.551874 0.000000

36 H 8.509853 9.201708 8.336619 5.264283 6.974800

37 H 9.519920 10.523915 9.418649 5.946089 8.729174

38 H 8.642101 9.773604 8.494232 4.978554 8.526602

39 H 9.965978 10.915401 9.597715 6.205524 9.574690

40 H 8.714452 9.293223 7.878372 5.100876 8.439875

41 H 7.957871 8.825935 7.323681 4.226238 8.207014

42 H 6.926227 7.553589 6.153993 3.334476 6.817178

36 37 38 39 40

36 H 0.000000

37 H 2.476437 0.000000

38 H 3.501732 1.769421 0.000000

39 H 3.545735 1.769507 1.780384 0.000000

40 H 3.937898 3.990459 3.567587 2.979383 0.000000

41 H 4.224875 3.645842 2.481535 2.711747 1.766842

42 H 3.755283 4.257041 3.540594 3.819712 1.791634

41 42

41 H 0.000000

42 H 1.743034 0.000000

Stoichiometry C15H24O3

Framework group C1[X(C15H24O3)]

Deg. of freedom 120

Full point group C1 NOp 1

Largest Abelian subgroup C1 NOp 1

Largest concise Abelian subgroup C1 NOp 1

Standard orientation:

Center Number	Atomic Number	Atomic Type	Coordinates (Angstroms) X	Y	Z
1	8	0	1.199324	-2.205277	0.279485
2	8	0	3.866055	-0.203098	-1.240140
3	8	0	-2.379676	1.050568	1.468819
4	6	0	0.653998	0.740092	0.498051
5	6	0	0.823404	-0.076862	-0.797836
6	6	0	1.781775	-1.259724	-0.615957
7	6	0	-0.019460	2.078039	0.187318
8	6	0	3.174936	-0.790585	-0.130516
9	6	0	1.961685	0.953425	1.214952
10	6	0	3.086873	0.285193	0.923294
11	6	0	-1.351214	1.876437	-0.551465
12	6	0	0.917776	2.949282	-0.650663
13	6	0	4.022994	-1.962005	0.377177
14	6	0	-2.350019	1.049947	0.241314
15	6	0	-3.344596	0.287097	-0.566081
16	6	0	-3.892962	-0.886459	-0.205434
17	6	0	-4.896362	-1.672821	-1.002102
18	6	0	-3.526088	-1.511396	1.115285
19	1	0	0.023940	0.164972	1.187882
20	1	0	-0.151619	-0.479011	-1.099142
21	1	0	1.188691	0.524077	-1.634257
22	1	0	1.892242	-1.765393	-1.583713
23	1	0	-0.216358	2.603760	1.131831
24	1	0	1.986768	1.684203	2.019594
25	1	0	3.990477	0.529288	1.476767
26	1	0	-1.201212	1.434276	-1.542194
27	1	0	-1.821164	2.854662	-0.717749

28	1	0	1.891291	3.110868	-0.178861
29	1	0	0.470341	3.943840	-0.770948
30	1	0	1.080888	2.569528	-1.662656
31	1	0	4.104677	-2.738905	-0.391818
32	1	0	5.048176	-1.639494	0.595864
33	1	0	3.606778	-2.412957	1.284186
34	1	0	0.342799	-2.475667	-0.092946
35	1	0	3.385447	0.593530	-1.521888
36	1	0	-3.588283	0.715069	-1.534637
37	1	0	-5.155844	-1.160005	-1.934125
38	1	0	-4.493073	-2.656778	-1.262367
39	1	0	-5.817835	-1.809812	-0.427249
40	1	0	-4.133437	-1.089941	1.922498
41	1	0	-3.712207	-2.591577	1.092206
42	1	0	-2.463541	-1.404035	1.354394

Rotational constants (GHZ): 0.6663695 0.2347885 0.1992504

Standard basis: 6-311+G(2d,p) (5D, 7F)

There are 666 symmetry adapted cartesian basis functions of A symmetry.

There are 630 symmetry adapted basis functions of A symmetry.

630 basis functions, 948 primitive gaussians, 666 cartesian basis functions

69 alpha electrons 69 beta electrons

nuclear repulsion energy 1412.1180923296 Hartrees.

NAtoms= 42 NActive= 42 NUniq= 42 SFac= 1.00D+00 NAtFMM= 60 NAOKFM=F Big=F

Integral buffers will be 262144 words long.

Raffenetti 2 integral format.

Two-electron integral symmetry is turned on.

Using the following non-standard input for PCM:

--- end of non-standard input.

Polarizable Continuum Model (PCM)

=================================

Model : PCM (using non-symmetric T matrix).

Atomic radii : SMD-Coulomb.

Polarization charges : Total charges.

Charge compensation : None.

Solution method : On-the-fly selection.

Cavity type : VdW (van der Waals Surface) (Alpha=1.000).

Cavity algorithm : GePol (No added spheres)

Default sphere list used, NSphG= 42.

Lebedev-Laikov grids with approx. 5.0 points / Ang**2.

Smoothing algorithm: Karplus/York (Gamma=1.0000).

Polarization charges: spherical gaussians, with

point-specific exponents (IZeta= 3).

Self-potential: point-specific (ISelfS= 7).

Self-field : sphere-specific E.n sum rule (ISelfD= 2).

Solvent : Water, Eps= 78.355300 Eps(inf)= 1.777849

Atomic radii for non-electrostatic terms: SMD-CDS.

Nuclear repulsion after PCM non-electrostatic terms = 1412.1291656598 Hartrees.

One-electron integrals computed using PRISM.

NBasis= 630 RedAO= T EigKep= 1.48D-06 NBF= 630

NBsUse= 630 1.00D-06 EigRej= -1.00D+00 NBFU= 630

ExpMin= 4.38D-02 ExpMax= 8.59D+03 ExpMxC= 1.30D+03 IAcc=2 IRadAn= 4 AccDes= 0.00D+00

Harris functional with IExCor= 402 and IRadAn= 4 diagonalized for initial guess.

HarFok: IExCor= 402 AccDes= 0.00D+00 IRadAn= 4 IDoV= 1 UseB2=F ITyADJ=14

ICtDFT= 3500011 ScaDFX= 1.000000 1.000000 1.000000 1.000000

FoFCou: FMM=F IPFlag= 0 FMFlag= 100000 FMFlg1= 0

NFxFlg= 0 DoJE=T BraDBF=F KetDBF=T FulRan=T

wScrn= 0.000000 ICntrl= 500 IOpCl= 0 I1Cent= 200000004 NGrid= 0

NMat0= 1 NMatS0= 1 NMatT0= 0 NMatD0= 1 NMtDS0= 0 NMtDT0= 0

Petite list used in FoFCou.

Requested convergence on RMS density matrix=1.00D-08 within 128 cycles.

Requested convergence on MAX density matrix=1.00D-06.

Requested convergence on energy=1.00D-06.

No special actions if energy rises.

Inv3: Mode=1 IEnd= 21902412.

Iteration 1 A*A^-1 deviation from unit magnitude is 2.89D-15 for 1607.

Iteration 1 A*A^-1 deviation from orthogonality is 2.40D-15 for 1424 147.

Iteration 1 A^-1*A deviation from unit magnitude is 2.22D-15 for 654.

Iteration 1 A^-1*A deviation from orthogonality is 7.37D-10 for 2656 2645.

Iteration 2 A*A^-1 deviation from unit magnitude is 2.89D-15 for 34.

Iteration 2 A*A^-1 deviation from orthogonality is 4.02D-15 for 1952 67.

Iteration 2 A^-1*A deviation from unit magnitude is 1.22D-15 for 1163.

Iteration 2 A^-1*A deviation from orthogonality is 4.94D-16 for 1300 277.

EnCoef did 6 forward-backward iterations

Error on total polarization charges = 0.07790

SCF Done: E(RB3LYP) = -811.954291957 A.U. after 14 cycles

NFock= 14 Conv=0.25D-08 -V/T= 2.0044

SMD-CDS (non-electrostatic) energy (kcal/mol) = 6.95

(included in total energy above)

****

Population analysis using the SCF density.

****

Orbital symmetries:

Occupied (A) (A) (A) (A) (A) (A) (A) (A) (A) (A) (A) (A)

(A) (A) (A) (A) (A) (A) (A) (A) (A) (A) (A) (A)

(A) (A) (A) (A) (A) (A) (A) (A) (A) (A) (A) (A)

(A) (A) (A) (A) (A) (A) (A) (A) (A) (A) (A) (A)

(A) (A) (A) (A) (A) (A) (A) (A) (A) (A) (A) (A)

(A) (A) (A) (A) (A) (A) (A) (A) (A)

Virtual (A) (A) (A) (A) (A) (A) (A) (A) (A) (A) (A) (A)

(A) (A) (A) (A) (A) (A) (A) (A) (A) (A) (A) (A)

(A) (A) (A) (A) (A) (A) (A) (A) (A) (A) (A) (A)

(A) (A) (A) (A) (A) (A) (A) (A) (A) (A) (A) (A)

(A) (A) (A) (A) (A) (A) (A) (A) (A) (A) (A) (A)

(A) (A) (A) (A) (A) (A) (A) (A) (A) (A) (A) (A)

(A) (A) (A) (A) (A) (A) (A) (A) (A) (A) (A) (A)

(A) (A) (A) (A) (A) (A) (A) (A) (A) (A) (A) (A)

(A) (A) (A) (A) (A) (A) (A) (A) (A) (A) (A) (A)

(A) (A) (A) (A) (A) (A) (A) (A) (A) (A) (A) (A)

(A) (A) (A) (A) (A) (A) (A) (A) (A) (A) (A) (A)

(A) (A) (A) (A) (A) (A) (A) (A) (A) (A) (A) (A)

(A) (A) (A) (A) (A) (A) (A) (A) (A) (A) (A) (A)

(A) (A) (A) (A) (A) (A) (A) (A) (A) (A) (A) (A)

(A) (A) (A) (A) (A) (A) (A) (A) (A) (A) (A) (A)

(A) (A) (A) (A) (A) (A) (A) (A) (A) (A) (A) (A)

(A) (A) (A) (A) (A) (A) (A) (A) (A) (A) (A) (A)

(A) (A) (A) (A) (A) (A) (A) (A) (A) (A) (A) (A)

(A) (A) (A) (A) (A) (A) (A) (A) (A) (A) (A) (A)

(A) (A) (A) (A) (A) (A) (A) (A) (A) (A) (A) (A)

(A) (A) (A) (A) (A) (A) (A) (A) (A) (A) (A) (A)

(A) (A) (A) (A) (A) (A) (A) (A) (A) (A) (A) (A)

(A) (A) (A) (A) (A) (A) (A) (A) (A) (A) (A) (A)

(A) (A) (A) (A) (A) (A) (A) (A) (A) (A) (A) (A)

(A) (A) (A) (A) (A) (A) (A) (A) (A) (A) (A) (A)

(A) (A) (A) (A) (A) (A) (A) (A) (A) (A) (A) (A)

(A) (A) (A) (A) (A) (A) (A) (A) (A) (A) (A) (A)

(A) (A) (A) (A) (A) (A) (A) (A) (A) (A) (A) (A)

(A) (A) (A) (A) (A) (A) (A) (A) (A) (A) (A) (A)

(A) (A) (A) (A) (A) (A) (A) (A) (A) (A) (A) (A)

(A) (A) (A) (A) (A) (A) (A) (A) (A) (A) (A) (A)

(A) (A) (A) (A) (A) (A) (A) (A) (A) (A) (A) (A)

(A) (A) (A) (A) (A) (A) (A) (A) (A) (A) (A) (A)

(A) (A) (A) (A) (A) (A) (A) (A) (A) (A) (A) (A)

(A) (A) (A) (A) (A) (A) (A) (A) (A) (A) (A) (A)

(A) (A) (A) (A) (A) (A) (A) (A) (A) (A) (A) (A)

(A) (A) (A) (A) (A) (A) (A) (A) (A) (A) (A) (A)

(A) (A) (A) (A) (A) (A) (A) (A) (A) (A) (A) (A)

(A) (A) (A) (A) (A) (A) (A) (A) (A) (A) (A) (A)

(A) (A) (A) (A) (A) (A) (A) (A) (A) (A) (A) (A)

(A) (A) (A) (A) (A) (A) (A) (A) (A) (A) (A) (A)

(A) (A) (A) (A) (A) (A) (A) (A) (A) (A) (A) (A)

(A) (A) (A) (A) (A) (A) (A) (A) (A) (A) (A) (A)

(A) (A) (A) (A) (A) (A) (A) (A) (A) (A) (A) (A)

(A) (A) (A) (A) (A) (A) (A) (A) (A) (A) (A) (A)

(A) (A) (A) (A) (A) (A) (A) (A) (A) (A) (A) (A)

(A) (A) (A) (A) (A) (A) (A) (A) (A)

The electronic state is 1-A.

Alpha occ. eigenvalues -- -19.12967 -19.12560 -19.11572 -10.26340 -10.23295

Alpha occ. eigenvalues -- -10.21673 -10.19886 -10.17052 -10.16717 -10.16671

Alpha occ. eigenvalues -- -10.16635 -10.16411 -10.16317 -10.16026 -10.15733

Alpha occ. eigenvalues -- -10.15564 -10.15541 -10.14725 -1.04634 -1.03644

Alpha occ. eigenvalues -- -1.02333 -0.83989 -0.81955 -0.79589 -0.75360

Alpha occ. eigenvalues -- -0.73844 -0.72701 -0.69313 -0.68391 -0.67721

Alpha occ. eigenvalues -- -0.62365 -0.60399 -0.58467 -0.54915 -0.54450

Alpha occ. eigenvalues -- -0.51084 -0.50792 -0.49240 -0.48097 -0.47433

Alpha occ. eigenvalues -- -0.46318 -0.45415 -0.44796 -0.44252 -0.43361

Alpha occ. eigenvalues -- -0.42793 -0.41816 -0.40708 -0.40518 -0.39704

Alpha occ. eigenvalues -- -0.39161 -0.38585 -0.38540 -0.37474 -0.37235

Alpha occ. eigenvalues -- -0.36470 -0.36127 -0.35247 -0.34735 -0.34533

Alpha occ. eigenvalues -- -0.33143 -0.32432 -0.31945 -0.31755 -0.28334

Alpha occ. eigenvalues -- -0.27460 -0.26011 -0.25811 -0.24822

Alpha virt. eigenvalues -- -0.05381 -0.00108 0.00421 0.01267 0.01560

Alpha virt. eigenvalues -- 0.02126 0.02762 0.03201 0.03672 0.04134

Alpha virt. eigenvalues -- 0.04402 0.04864 0.05245 0.05784 0.06100

Alpha virt. eigenvalues -- 0.06386 0.06771 0.07059 0.07613 0.07875

Alpha virt. eigenvalues -- 0.08034 0.08753 0.09045 0.09303 0.09865

Alpha virt. eigenvalues -- 0.10170 0.10606 0.10961 0.11103 0.11484

Alpha virt. eigenvalues --	0.11754	0.11846	0.12601	0.13083	0.13584
Alpha virt. eigenvalues --	0.13678	0.14247	0.14650	0.15088	0.15611
Alpha virt. eigenvalues --	0.15927	0.16133	0.16947	0.17098	0.17372
Alpha virt. eigenvalues --	0.17749	0.18195	0.18369	0.18520	0.19005
Alpha virt. eigenvalues --	0.19167	0.19319	0.19604	0.20201	0.20285
Alpha virt. eigenvalues --	0.20442	0.20673	0.20877	0.21089	0.21715
Alpha virt. eigenvalues --	0.21933	0.22216	0.22367	0.22507	0.22928
Alpha virt. eigenvalues --	0.23128	0.23489	0.23689	0.23843	0.24042
Alpha virt. eigenvalues --	0.24473	0.24878	0.25194	0.25742	0.26055
Alpha virt. eigenvalues --	0.26556	0.26681	0.26744	0.26891	0.27038
Alpha virt. eigenvalues --	0.27749	0.28293	0.28363	0.28585	0.28912
Alpha virt. eigenvalues --	0.29354	0.29877	0.30345	0.30536	0.30763
Alpha virt. eigenvalues --	0.31356	0.31916	0.32518	0.33051	0.33472
Alpha virt. eigenvalues --	0.33659	0.33923	0.34052	0.34970	0.35473
Alpha virt. eigenvalues --	0.35779	0.36037	0.36309	0.36535	0.37280
Alpha virt. eigenvalues --	0.37598	0.39148	0.40279	0.40714	0.41038
Alpha virt. eigenvalues --	0.41994	0.42817	0.43157	0.43647	0.43990
Alpha virt. eigenvalues --	0.44705	0.44996	0.45332	0.46738	0.47614
Alpha virt. eigenvalues --	0.47809	0.48528	0.49029	0.49817	0.50143
Alpha virt. eigenvalues --	0.50620	0.51098	0.51504	0.52679	0.53124
Alpha virt. eigenvalues --	0.53946	0.54873	0.55510	0.55712	0.56017
Alpha virt. eigenvalues --	0.56548	0.57198	0.57715	0.58222	0.58529
Alpha virt. eigenvalues --	0.59309	0.59582	0.60118	0.61025	0.61364
Alpha virt. eigenvalues --	0.61510	0.62095	0.62859	0.63440	0.64027
Alpha virt. eigenvalues --	0.64500	0.65031	0.65321	0.65669	0.66208
Alpha virt. eigenvalues --	0.66346	0.66673	0.67285	0.67553	0.68015
Alpha virt. eigenvalues --	0.68623	0.69336	0.69718	0.70447	0.70741
Alpha virt. eigenvalues --	0.70969	0.71677	0.71793	0.72224	0.72698
Alpha virt. eigenvalues --	0.72852	0.73903	0.74206	0.75019	0.75430
Alpha virt. eigenvalues --	0.75989	0.76318	0.77052	0.77553	0.77774
Alpha virt. eigenvalues --	0.79160	0.79457	0.80249	0.80643	0.81182
Alpha virt. eigenvalues --	0.81893	0.82243	0.83110	0.84187	0.85831
Alpha virt. eigenvalues --	0.86228	0.86800	0.87176	0.88235	0.89486

Alpha virt. eigenvalues -- 0.90047 0.90579 0.91839 0.93642 0.94821

Alpha virt. eigenvalues -- 0.95368 0.95794 0.96278 0.97008 0.98552

Alpha virt. eigenvalues -- 0.99684 1.00449 1.00624 1.00874 1.02563

Alpha virt. eigenvalues -- 1.03378 1.04566 1.05333 1.06151 1.07108

Alpha virt. eigenvalues -- 1.08147 1.09076 1.09425 1.10354 1.10855

Alpha virt. eigenvalues -- 1.12208 1.13212 1.14089 1.14657 1.14978

Alpha virt. eigenvalues -- 1.15187 1.15745 1.16547 1.17122 1.19056

Alpha virt. eigenvalues -- 1.19552 1.20409 1.21197 1.22037 1.22700

Alpha virt. eigenvalues -- 1.23487 1.24250 1.24983 1.25170 1.25860

Alpha virt. eigenvalues -- 1.26497 1.26819 1.28170 1.29195 1.29485

Alpha virt. eigenvalues -- 1.29904 1.31682 1.32087 1.32565 1.32941

Alpha virt. eigenvalues -- 1.33389 1.33900 1.34577 1.34946 1.36176

Alpha virt. eigenvalues -- 1.36822 1.38674 1.38860 1.40109 1.40326

Alpha virt. eigenvalues -- 1.40825 1.41854 1.42998 1.43932 1.44971

Alpha virt. eigenvalues -- 1.46507 1.46930 1.47456 1.48770 1.48918

Alpha virt. eigenvalues -- 1.49732 1.51233 1.52075 1.52630 1.52956

Alpha virt. eigenvalues -- 1.54032 1.55225 1.56081 1.58017 1.58552

Alpha virt. eigenvalues -- 1.59633 1.59963 1.61135 1.62034 1.65803

Alpha virt. eigenvalues -- 1.66512 1.68075 1.68501 1.69367 1.71622

Alpha virt. eigenvalues -- 1.73680 1.74699 1.75818 1.76528 1.76848

Alpha virt. eigenvalues -- 1.78454 1.79563 1.81029 1.82070 1.83909

Alpha virt. eigenvalues -- 1.85239 1.86007 1.86544 1.87269 1.88279

Alpha virt. eigenvalues -- 1.89373 1.90033 1.90262 1.91337 1.92812

Alpha virt. eigenvalues -- 1.94588 1.95348 1.97005 1.98844 2.01477

Alpha virt. eigenvalues -- 2.01677 2.03321 2.05451 2.06794 2.08126

Alpha virt. eigenvalues -- 2.09385 2.10006 2.12120 2.13781 2.13878

Alpha virt. eigenvalues -- 2.15792 2.16753 2.18671 2.20031 2.22115

Alpha virt. eigenvalues -- 2.22995 2.23354 2.24963 2.25248 2.26062

Alpha virt. eigenvalues -- 2.27295 2.28826 2.29858 2.30236 2.30843

Alpha virt. eigenvalues -- 2.32376 2.32516 2.33236 2.34976 2.35182

Alpha virt. eigenvalues -- 2.36104 2.37283 2.37592 2.38179 2.39437

Alpha virt. eigenvalues -- 2.39997 2.41284 2.41934 2.42929 2.43190

Alpha virt. eigenvalues -- 2.43908 2.44498 2.45150 2.46342 2.46998

Alpha virt. eigenvalues -- 2.49723 2.50522 2.52442 2.53899 2.55687
Alpha virt. eigenvalues -- 2.55830 2.58538 2.60602 2.61405 2.62067
Alpha virt. eigenvalues -- 2.63258 2.65708 2.68153 2.69878 2.71105
Alpha virt. eigenvalues -- 2.71664 2.72125 2.73298 2.74311 2.76010
Alpha virt. eigenvalues -- 2.77158 2.78413 2.80025 2.83890 2.84356
Alpha virt. eigenvalues -- 2.86006 2.86741 2.87859 2.88779 2.89675
Alpha virt. eigenvalues -- 2.90843 2.91682 2.92620 2.92945 2.94736
Alpha virt. eigenvalues -- 2.96045 2.97917 3.00239 3.01087 3.01847
Alpha virt. eigenvalues -- 3.02894 3.04209 3.06508 3.08313 3.09961
Alpha virt. eigenvalues -- 3.10400 3.12644 3.14247 3.14415 3.16766
Alpha virt. eigenvalues -- 3.17816 3.19123 3.20912 3.21885 3.24931
Alpha virt. eigenvalues -- 3.26172 3.27381 3.28845 3.30107 3.30465
Alpha virt. eigenvalues -- 3.32523 3.33129 3.34881 3.35919 3.36987
Alpha virt. eigenvalues -- 3.37363 3.38928 3.39650 3.40675 3.41951
Alpha virt. eigenvalues -- 3.42164 3.43982 3.45412 3.46501 3.48160
Alpha virt. eigenvalues -- 3.48427 3.49812 3.50026 3.50752 3.51186
Alpha virt. eigenvalues -- 3.52801 3.53512 3.53781 3.55185 3.56054
Alpha virt. eigenvalues -- 3.57028 3.57814 3.59044 3.60439 3.62118
Alpha virt. eigenvalues -- 3.62967 3.63623 3.64904 3.65838 3.66023
Alpha virt. eigenvalues -- 3.66551 3.67684 3.68354 3.69034 3.70325
Alpha virt. eigenvalues -- 3.70908 3.71586 3.72856 3.73936 3.75729
Alpha virt. eigenvalues -- 3.76333 3.77338 3.78934 3.79275 3.80011
Alpha virt. eigenvalues -- 3.81512 3.83348 3.84453 3.85191 3.85928
Alpha virt. eigenvalues -- 3.86538 3.89226 3.89988 3.90535 3.91930
Alpha virt. eigenvalues -- 3.92847 3.93733 3.95478 3.95557 3.99080
Alpha virt. eigenvalues -- 4.00714 4.03190 4.04198 4.04959 4.08109
Alpha virt. eigenvalues -- 4.09336 4.14209 4.15477 4.16485 4.16831
Alpha virt. eigenvalues -- 4.21940 4.22157 4.24185 4.24604 4.25671
Alpha virt. eigenvalues -- 4.27329 4.28914 4.29801 4.31910 4.32448
Alpha virt. eigenvalues -- 4.33882 4.35715 4.40376 4.41995 4.45784
Alpha virt. eigenvalues -- 4.51449 4.52086 4.57301 4.57656 4.58557
Alpha virt. eigenvalues -- 4.62438 4.67259 4.92078 4.95818 5.14172
Alpha virt. eigenvalues -- 5.17617 5.19397 5.38327 5.44782 5.51178

Alpha virt. eigenvalues -- 5.79333 5.84105 6.03707 6.87132 6.90505

Alpha virt. eigenvalues -- 6.92527 6.94093 7.03478 7.04212 7.07701

Alpha virt. eigenvalues -- 7.07860 7.11095 7.19099 7.23420 7.25029

Alpha virt. eigenvalues -- 7.28932 7.38190 7.38501 23.75235 23.83141

Alpha virt. eigenvalues -- 23.89351 23.91402 23.96358 23.98392 24.00080

Alpha virt. eigenvalues -- 24.02067 24.06813 24.09013 24.10717 24.13707

Alpha virt. eigenvalues -- 24.16038 24.24318 24.26839 50.01995 50.04381

Alpha virt. eigenvalues -- 50.05638

Condensed to atoms (all electrons):

		1	2	3	4	5	6
1	O	8.200678	0.001270	-0.000556	-0.056703	-0.049611	0.167953
2	O	0.001270	8.262538	0.000011	0.100548	0.049880	-0.085440
3	O	-0.000556	0.000011	8.282489	0.002655	0.006577	0.003890
4	C	-0.056703	0.100548	0.002655	11.322463	-0.741637	0.808102
5	C	-0.049611	0.049880	0.006577	-0.741637	7.282569	-1.350371
6	C	0.167953	-0.085440	0.003890	0.808102	-1.350371	8.161755
7	C	0.053591	-0.017655	-0.075001	-2.044966	0.334945	-0.463031
8	C	0.009817	0.216583	-0.001251	-2.166047	0.435432	-1.555933
9	C	0.003629	-0.179946	-0.032754	-4.011526	0.113089	-0.507589
10	C	0.046979	0.116374	-0.003012	1.024998	-0.170968	0.657189
11	C	-0.024105	0.003047	0.044520	0.961075	-0.211594	0.205942
12	C	0.002348	0.018292	0.007174	0.223860	-0.131789	-0.037944
13	C	0.003964	-0.225081	-0.000011	-0.037669	-0.118922	0.003938
14	C	-0.012126	-0.000763	0.559186	-0.129084	0.078813	0.139866
15	C	0.005506	0.000174	-0.170167	0.183743	0.038917	-0.154983
16	C	-0.004131	0.000023	-0.050214	-0.039672	-0.014493	0.043970
17	C	0.000461	0.000006	-0.015684	-0.009476	0.013864	-0.003544
18	C	0.002153	0.000032	0.007455	0.047734	-0.004876	-0.023040
19	H	-0.004312	-0.001233	-0.003879	0.397310	-0.026344	0.021128
20	H	-0.013749	-0.000397	-0.000216	-0.072605	0.420879	0.022008
21	H	0.007442	-0.002519	-0.000042	-0.122779	0.476185	-0.077748
22	H	-0.058584	-0.002359	-0.000004	-0.013582	-0.006278	0.429197
23	H	0.000250	0.000031	0.005405	-0.050109	-0.008076	-0.012217

24	H	0.000659	-0.000600	-0.000075	-0.064952	-0.023509	0.009332
25	H	-0.001333	0.004376	0.000003	0.007441	0.014808	-0.008748
26	H	0.000277	-0.000013	0.003270	-0.017175	0.001026	-0.000764
27	H	-0.000020	0.000004	-0.004630	0.019249	0.010422	0.000700
28	H	0.000025	-0.000289	0.000282	-0.085197	0.008096	-0.000019
29	H	-0.000003	-0.000082	-0.000028	0.008753	0.007768	0.001252
30	H	-0.000013	-0.000185	0.000092	0.025252	-0.027120	0.001999
31	H	-0.001643	-0.002768	0.000000	0.006932	0.007200	-0.054492
32	H	-0.000479	-0.002169	0.000000	-0.000737	-0.003605	0.026992
33	H	-0.004129	0.007838	0.000000	0.001327	-0.011429	0.022635
34	H	0.260880	-0.000082	-0.000011	-0.003948	-0.010039	0.043851
35	H	-0.000185	0.263419	-0.000002	-0.006259	-0.000544	-0.022172
36	H	-0.000008	0.000000	0.004991	0.005508	-0.002481	0.000021
37	H	0.000000	0.000000	-0.000013	-0.000415	0.000433	-0.000012
38	H	0.000010	0.000000	0.000160	-0.000377	0.000139	0.000383
39	H	0.000000	0.000000	0.000160	0.000309	-0.000333	-0.000060
40	H	-0.000016	0.000000	-0.005140	0.000350	-0.000092	-0.000093
41	H	0.000075	0.000000	0.003236	-0.000960	0.000028	0.000695
42	H	0.000089	-0.000001	-0.006187	0.002114	0.002071	-0.001247

		7	8	9	10	11	12
1	O	0.053591	0.009817	0.003629	0.046979	-0.024105	0.002348
2	O	-0.017655	0.216583	-0.179946	0.116374	0.003047	0.018292
3	O	-0.075001	-0.001251	-0.032754	-0.003012	0.044520	0.007174
4	C	-2.044966	-2.166047	-4.011526	1.024998	0.961075	0.223860
5	C	0.334945	0.435432	0.113089	-0.170968	-0.211594	-0.131789
6	C	-0.463031	-1.555933	-0.507589	0.657189	0.205942	-0.037944
7	C	8.418343	0.421291	1.063604	0.070459	-2.705387	-0.123198
8	C	0.421291	8.933552	1.604594	-2.203498	-0.079192	-0.130319
9	C	1.063604	1.604594	10.292191	-1.719211	-0.497923	-0.421593
10	C	0.070459	-2.203498	-1.719211	8.748309	-0.183000	0.168293
11	C	-2.705387	-0.079192	-0.497923	-0.183000	9.124808	0.148989
12	C	-0.123198	-0.130319	-0.421593	0.168293	0.148989	5.945698
13	C	0.007116	-0.113080	0.192275	-0.367205	0.013241	-0.006905

14	C	0.643742	-0.009120	-0.171447	-0.033905	-0.895985	-0.152773
15	C	0.037982	0.000022	0.078590	0.035751	-0.079891	-0.033765
16	C	-0.208677	-0.004519	-0.049455	-0.009010	0.184751	-0.001105
17	C	0.014092	0.001713	0.000291	-0.000019	-0.067289	-0.002461
18	C	-0.016006	-0.000134	0.019126	0.003893	-0.021427	0.005148
19	H	0.024858	0.022355	-0.043538	-0.059843	-0.002416	-0.021696
20	H	-0.020267	-0.011573	0.019533	-0.007975	-0.011700	0.000986
21	H	0.068015	0.055877	0.021942	-0.016850	-0.018581	-0.028202
22	H	0.001920	-0.062745	-0.002152	0.020969	-0.005562	0.002520
23	H	0.484721	0.003439	0.002527	0.013454	-0.083552	-0.020749
24	H	0.014889	-0.000685	0.485773	-0.053966	-0.015565	-0.015336
25	H	-0.003936	-0.032344	-0.023515	0.403587	0.000957	0.001709
26	H	-0.072735	0.003510	0.003533	0.001179	0.517195	-0.015632
27	H	-0.109110	-0.000517	0.000339	0.000194	0.486389	-0.007040
28	H	-0.044014	0.007239	0.052870	-0.009900	0.023012	0.431318
29	H	-0.075023	0.001377	0.010771	-0.002604	0.016005	0.407216
30	H	0.051951	0.000731	-0.012287	0.009564	-0.083688	0.400744
31	H	0.000249	-0.021946	-0.009581	0.029547	-0.000107	0.000231
32	H	-0.000252	-0.063048	0.024075	-0.041166	0.000063	-0.000003
33	H	0.000600	-0.086293	-0.010514	0.033272	-0.000129	-0.000006
34	H	0.000176	-0.043163	0.006686	0.030752	-0.006183	-0.000169
35	H	0.000986	0.018181	0.036725	-0.050251	0.001846	0.000412
36	H	-0.007423	-0.000122	-0.000512	0.000034	0.020251	0.000465
37	H	0.000759	0.000001	0.000007	-0.000001	0.000991	0.000016
38	H	-0.000084	0.000010	-0.000043	-0.000013	0.001334	0.000010
39	H	0.000011	-0.000004	0.000016	0.000004	-0.000333	0.000000
40	H	0.000299	-0.000085	0.000377	0.000164	-0.006642	-0.000108
41	H	0.000736	0.000063	-0.000294	-0.000123	0.004488	0.000040
42	H	-0.006756	0.000272	-0.002011	-0.000315	-0.009080	-0.000499

		13	14	15	16	17	18
1	O	0.003964	-0.012126	0.005506	-0.004131	0.000461	0.002153
2	O	-0.225081	-0.000763	0.000174	0.000023	0.000006	0.000032
3	O	-0.000011	0.559186	-0.170167	-0.050214	-0.015684	0.007455

4	C	-0.037669	-0.129084	0.183743	-0.039672	-0.009476	0.047734
5	C	-0.118922	0.078813	0.038917	-0.014493	0.013864	-0.004876
6	C	0.003938	0.139866	-0.154983	0.043970	-0.003544	-0.023040
7	C	0.007116	0.643742	0.037982	-0.208677	0.014092	-0.016006
8	C	-0.113080	-0.009120	0.000022	-0.004519	0.001713	-0.000134
9	C	0.192275	-0.171447	0.078590	-0.049455	0.000291	0.019126
10	C	-0.367205	-0.033905	0.035751	-0.009010	-0.000019	0.003893
11	C	0.013241	-0.895985	-0.079891	0.184751	-0.067289	-0.021427
12	C	-0.006905	-0.152773	-0.033765	-0.001105	-0.002461	0.005148
13	C	5.895260	0.000808	-0.001363	0.000527	0.000120	-0.000190
14	C	0.000808	6.877542	-1.613983	0.537382	-0.166425	-0.165661
15	C	-0.001363	-1.613983	8.347908	-0.929223	0.308139	-0.171501
16	C	0.000527	0.537382	-0.929223	6.109903	-0.189518	0.232223
17	C	0.000120	-0.166425	0.308139	-0.189518	5.655061	-0.128906
18	C	-0.000190	-0.165661	-0.171501	0.232223	-0.128906	5.761988
19	H	0.005304	0.005071	0.000604	-0.001430	-0.000551	-0.003110
20	H	0.002307	0.016473	0.004735	0.006246	-0.000132	-0.004356
21	H	-0.000368	-0.008500	0.005649	-0.001612	-0.000058	0.000100
22	H	-0.008521	-0.000673	0.000958	-0.000771	0.000069	0.000465
23	H	-0.000356	-0.036631	0.017704	-0.004103	0.000279	0.000163
24	H	-0.000382	0.003225	-0.000049	-0.000086	0.000003	-0.000021
25	H	-0.019836	-0.000152	-0.000018	0.000004	0.000000	0.000001
26	H	0.000113	-0.115020	0.037267	-0.002084	0.001340	-0.002071
27	H	-0.000025	-0.055886	0.000811	-0.001961	-0.000259	0.000776
28	H	-0.000134	-0.002780	0.000340	0.000010	-0.000003	0.000004
29	H	0.000084	-0.004852	-0.000685	0.000438	-0.000017	-0.000019
30	H	-0.000485	0.005358	0.001577	-0.000576	0.000102	-0.000036
31	H	0.411761	0.000004	-0.000001	0.000003	0.000001	0.000001
32	H	0.434371	0.000011	-0.000001	0.000000	0.000000	0.000000
33	H	0.422163	-0.000004	0.000011	0.000000	0.000000	-0.000003
34	H	-0.004800	-0.009135	0.008361	-0.005846	0.000634	0.004642
35	H	0.066954	-0.000131	-0.000062	0.000025	0.000000	0.000000
36	H	-0.000010	-0.027424	0.366057	-0.029169	-0.014173	0.013738

37	H	0.000000	0.007815	0.043954	-0.131674	0.449865	-0.003346
38	H	0.000004	0.004446	-0.000402	0.004763	0.375046	-0.022338
39	H	-0.000001	-0.001231	-0.033467	0.006874	0.387220	0.006162
40	H	-0.000009	0.001135	-0.012580	-0.048111	0.010196	0.423543
41	H	0.000021	-0.013320	0.094458	-0.086815	0.003388	0.358548
42	H	0.000052	0.037468	-0.093182	0.021352	-0.018079	0.443970

		19	20	21	22	23	24
1	O	-0.004312	-0.013749	0.007442	-0.058584	0.000250	0.000659
2	O	-0.001233	-0.000397	-0.002519	-0.002359	0.000031	-0.000600
3	O	-0.003879	-0.000216	-0.000042	-0.000004	0.005405	-0.000075
4	C	0.397310	-0.072605	-0.122779	-0.013582	-0.050109	-0.064952
5	C	-0.026344	0.420879	0.476185	-0.006278	-0.008076	-0.023509
6	C	0.021128	0.022008	-0.077748	0.429197	-0.012217	0.009332
7	C	0.024858	-0.020267	0.068015	0.001920	0.484721	0.014889
8	C	0.022355	-0.011573	0.055877	-0.062745	0.003439	-0.000685
9	C	-0.043538	0.019533	0.021942	-0.002152	0.002527	0.485773
10	C	-0.059843	-0.007975	-0.016850	0.020969	0.013454	-0.053966
11	C	-0.002416	-0.011700	-0.018581	-0.005562	-0.083552	-0.015565
12	C	-0.021696	0.000986	-0.028202	0.002520	-0.020749	-0.015336
13	C	0.005304	0.002307	-0.000368	-0.008521	-0.000356	-0.000382
14	C	0.005071	0.016473	-0.008500	-0.000673	-0.036631	0.003225
15	C	0.000604	0.004735	0.005649	0.000958	0.017704	-0.000049
16	C	-0.001430	0.006246	-0.001612	-0.000771	-0.004103	-0.000086
17	C	-0.000551	-0.000132	-0.000058	0.000069	0.000279	0.000003
18	C	-0.003110	-0.004356	0.000100	0.000465	0.000163	-0.000021
19	H	0.587814	-0.008998	0.005999	-0.000782	-0.012165	-0.001533
20	H	-0.008998	0.542814	-0.033879	-0.008571	-0.000232	-0.000295
21	H	0.005999	-0.033879	0.537555	-0.005897	-0.000137	-0.000042
22	H	-0.000782	-0.008571	-0.005897	0.591236	0.000038	0.000102
23	H	-0.012165	-0.000232	-0.000137	0.000038	0.596462	0.003051
24	H	-0.001533	-0.000295	-0.000042	0.000102	0.003051	0.543460
25	H	-0.000356	0.000099	-0.000156	-0.000309	-0.000029	-0.009807
26	H	-0.000448	-0.003285	0.000565	0.000080	0.007613	0.000038

27	H	-0.000323	0.000355	-0.000282	0.000001	-0.007205	0.000022
28	H	0.000353	-0.000098	0.000346	0.000000	-0.004313	-0.001692
29	H	-0.000183	-0.000075	-0.000149	-0.000004	-0.008759	0.000008
30	H	-0.000289	0.000449	-0.000760	0.000065	0.006390	0.000130
31	H	-0.000047	-0.000037	-0.000022	0.003525	0.000000	0.000033
32	H	-0.000005	0.000022	-0.000028	-0.000468	0.000000	-0.000046
33	H	0.000000	0.000024	0.000019	0.000285	0.000000	-0.000016
34	H	-0.001831	0.000137	-0.000276	-0.006514	-0.000001	-0.000004
35	H	0.000081	-0.000148	0.000056	-0.001357	-0.000011	-0.000058
36	H	-0.000048	-0.000133	-0.000006	0.000001	0.000010	0.000000
37	H	0.000001	-0.000001	0.000000	0.000000	0.000000	0.000000
38	H	0.000001	-0.000031	0.000000	0.000000	0.000000	0.000000
39	H	0.000000	0.000007	0.000000	0.000000	0.000000	0.000000
40	H	-0.000024	0.000014	0.000000	0.000000	0.000004	0.000000
41	H	0.000009	-0.000010	0.000000	-0.000001	-0.000001	0.000000
42	H	0.000886	-0.000167	0.000002	-0.000010	0.000017	0.000002

		25	26	27	28	29	30
1	O	-0.001333	0.000277	-0.000020	0.000025	-0.000003	-0.000013
2	O	0.004376	-0.000013	0.000004	-0.000289	-0.000082	-0.000185
3	O	0.000003	0.003270	-0.004630	0.000282	-0.000028	0.000092
4	C	0.007441	-0.017175	0.019249	-0.085197	0.008753	0.025252
5	C	0.014808	0.001026	0.010422	0.008096	0.007768	-0.027120
6	C	-0.008748	-0.000764	0.000700	-0.000019	0.001252	0.001999
7	C	-0.003936	-0.072735	-0.109110	-0.044014	-0.075023	0.051951
8	C	-0.032344	0.003510	-0.000517	0.007239	0.001377	0.000731
9	C	-0.023515	0.003533	0.000339	0.052870	0.010771	-0.012287
10	C	0.403587	0.001179	0.000194	-0.009900	-0.002604	0.009564
11	C	0.000957	0.517195	0.486389	0.023012	0.016005	-0.083688
12	C	0.001709	-0.015632	-0.007040	0.431318	0.407216	0.400744
13	C	-0.019836	0.000113	-0.000025	-0.000134	0.000084	-0.000485
14	C	-0.000152	-0.115020	-0.055886	-0.002780	-0.004852	0.005358
15	C	-0.000018	0.037267	0.000811	0.000340	-0.000685	0.001577
16	C	0.000004	-0.002084	-0.001961	0.000010	0.000438	-0.000576

17	C	0.000000	0.001340	-0.000259	-0.000003	-0.000017	0.000102
18	C	0.000001	-0.002071	0.000776	0.000004	-0.000019	-0.000036
19	H	-0.000356	-0.000448	-0.000323	0.000353	-0.000183	-0.000289
20	H	0.000099	-0.003285	0.000355	-0.000098	-0.000075	0.000449
21	H	-0.000156	0.000565	-0.000282	0.000346	-0.000149	-0.000760
22	H	-0.000309	0.000080	0.000001	0.000000	-0.000004	0.000065
23	H	-0.000029	0.007613	-0.007205	-0.004313	-0.008759	0.006390
24	H	-0.009807	0.000038	0.000022	-0.001692	0.000008	0.000130
25	H	0.552141	0.000000	-0.000001	0.000100	-0.000007	0.000002
26	H	0.000000	0.513535	-0.035671	-0.000121	-0.000362	0.005139
27	H	-0.000001	-0.035671	0.521694	-0.000043	0.003689	0.000298
28	H	0.000100	-0.000121	-0.000043	0.534623	-0.026592	-0.031617
29	H	-0.000007	-0.000362	0.003689	-0.026592	0.557585	-0.032273
30	H	0.000002	0.005139	0.000298	-0.031617	-0.032273	0.528141
31	H	-0.000325	0.000000	0.000000	-0.000001	0.000000	-0.000001
32	H	0.003120	0.000000	0.000000	0.000000	0.000000	0.000000
33	H	0.000583	0.000000	0.000000	0.000000	0.000000	-0.000001
34	H	0.000016	-0.000032	0.000006	-0.000002	0.000000	-0.000008
35	H	-0.000201	0.000008	-0.000002	-0.000232	0.000035	-0.000292
36	H	0.000000	0.000915	0.000540	-0.000001	0.000002	0.000010
37	H	0.000000	0.000005	0.000002	0.000000	0.000000	0.000000
38	H	0.000000	-0.000007	-0.000004	0.000000	0.000000	0.000000
39	H	0.000000	0.000004	0.000003	0.000000	0.000000	0.000000
40	H	0.000000	0.000016	0.000028	0.000000	0.000000	0.000000
41	H	0.000000	-0.000011	-0.000001	0.000000	0.000000	0.000000
42	H	0.000000	0.000025	-0.000047	0.000001	0.000000	0.000001

		31	32	33	34	35	36
1	O	-0.001643	-0.000479	-0.004129	0.260880	-0.000185	-0.000008
2	O	-0.002768	-0.002169	0.007838	-0.000082	0.263419	0.000000
3	O	0.000000	0.000000	0.000000	-0.000011	-0.000002	0.004991
4	C	0.006932	-0.000737	0.001327	-0.003948	-0.006259	0.005508
5	C	0.007200	-0.003605	-0.011429	-0.010039	-0.000544	-0.002481
6	C	-0.054492	0.026992	0.022635	0.043851	-0.022172	0.000021

7	C	0.000249	-0.000252	0.000600	0.000176	0.000986	-0.007423
8	C	-0.021946	-0.063048	-0.086293	-0.043163	0.018181	-0.000122
9	C	-0.009581	0.024075	-0.010514	0.006686	0.036725	-0.000512
10	C	0.029547	-0.041166	0.033272	0.030752	-0.050251	0.000034
11	C	-0.000107	0.000063	-0.000129	-0.006183	0.001846	0.020251
12	C	0.000231	-0.000003	-0.000006	-0.000169	0.000412	0.000465
13	C	0.411761	0.434371	0.422163	-0.004800	0.066954	-0.000010
14	C	0.000004	0.000011	-0.000004	-0.009135	-0.000131	-0.027424
15	C	-0.000001	-0.000001	0.000011	0.008361	-0.000062	0.366057
16	C	0.000003	0.000000	0.000000	-0.005846	0.000025	-0.029169
17	C	0.000001	0.000000	0.000000	0.000634	0.000000	-0.014173
18	C	0.000001	0.000000	-0.000003	0.004642	0.000000	0.013738
19	H	-0.000047	-0.000005	0.000000	-0.001831	0.000081	-0.000048
20	H	-0.000037	0.000022	0.000024	0.000137	-0.000148	-0.000133
21	H	-0.000022	-0.000028	0.000019	-0.000276	0.000056	-0.000006
22	H	0.003525	-0.000468	0.000285	-0.006514	-0.001357	0.000001
23	H	0.000000	0.000000	0.000000	-0.000001	-0.000011	0.000010
24	H	0.000033	-0.000046	-0.000016	-0.000004	-0.000058	0.000000
25	H	-0.000325	0.003120	0.000583	0.000016	-0.000201	0.000000
26	H	0.000000	0.000000	0.000000	-0.000032	0.000008	0.000915
27	H	0.000000	0.000000	0.000000	0.000006	-0.000002	0.000540
28	H	-0.000001	0.000000	0.000000	-0.000002	-0.000232	-0.000001
29	H	0.000000	0.000000	0.000000	0.000000	0.000035	0.000002
30	H	-0.000001	0.000000	-0.000001	-0.000008	-0.000292	0.000010
31	H	0.534920	-0.029649	-0.025995	0.000113	-0.000193	0.000000
32	H	-0.029649	0.528178	-0.026766	0.000027	0.000140	0.000000
33	H	-0.025995	-0.026766	0.517869	0.000676	-0.000298	0.000000
34	H	0.000113	0.000027	0.000676	0.397786	0.000016	0.000007
35	H	-0.000193	0.000140	-0.000298	0.000016	0.384062	0.000000
36	H	0.000000	0.000000	0.000000	0.000007	0.000000	0.541755
37	H	0.000000	0.000000	0.000000	-0.000001	0.000000	0.005322
38	H	0.000000	0.000000	0.000000	-0.000006	0.000000	0.000108
39	H	0.000000	0.000000	0.000000	0.000001	0.000000	0.000102

40	H	0.000000	0.000000	0.000000	0.000003	0.000000	-0.000232
41	H	0.000000	0.000000	0.000000	-0.000056	0.000000	-0.000192
42	H	0.000000	0.000000	0.000000	-0.000310	0.000000	-0.000290

		37	38	39	40	41	42
1	O	0.000000	0.000010	0.000000	-0.000016	0.000075	0.000089
2	O	0.000000	0.000000	0.000000	0.000000	0.000000	-0.000001
3	O	-0.000013	0.000160	0.000160	-0.005140	0.003236	-0.006187
4	C	-0.000415	-0.000377	0.000309	0.000350	-0.000960	0.002114
5	C	0.000433	0.000139	-0.000333	-0.000092	0.000028	0.002071
6	C	-0.000012	0.000383	-0.000060	-0.000093	0.000695	-0.001247
7	C	0.000759	-0.000084	0.000011	0.000299	0.000736	-0.006756
8	C	0.000001	0.000010	-0.000004	-0.000085	0.000063	0.000272
9	C	0.000007	-0.000043	0.000016	0.000377	-0.000294	-0.002011
10	C	-0.000001	-0.000013	0.000004	0.000164	-0.000123	-0.000315
11	C	0.000991	0.001334	-0.000333	-0.006642	0.004488	-0.009080
12	C	0.000016	0.000010	0.000000	-0.000108	0.000040	-0.000499
13	C	0.000000	0.000004	-0.000001	-0.000009	0.000021	0.000052
14	C	0.007815	0.004446	-0.001231	0.001135	-0.013320	0.037468
15	C	0.043954	-0.000402	-0.033467	-0.012580	0.094458	-0.093182
16	C	-0.131674	0.004763	0.006874	-0.048111	-0.086815	0.021352
17	C	0.449865	0.375046	0.387220	0.010196	0.003388	-0.018079
18	C	-0.003346	-0.022338	0.006162	0.423543	0.358548	0.443970
19	H	0.000001	0.000001	0.000000	-0.000024	0.000009	0.000886
20	H	-0.000001	-0.000031	0.000007	0.000014	-0.000010	-0.000167
21	H	0.000000	0.000000	0.000000	0.000000	0.000000	0.000002
22	H	0.000000	0.000000	0.000000	0.000000	-0.000001	-0.000010
23	H	0.000000	0.000000	0.000000	0.000004	-0.000001	0.000017
24	H	0.000000	0.000000	0.000000	0.000000	0.000000	0.000002
25	H	0.000000	0.000000	0.000000	0.000000	0.000000	0.000000
26	H	0.000005	-0.000007	0.000004	0.000016	-0.000011	0.000025
27	H	0.000002	-0.000004	0.000003	0.000028	-0.000001	-0.000047
28	H	0.000000	0.000000	0.000000	0.000000	0.000000	0.000001
29	H	0.000000	0.000000	0.000000	0.000000	0.000000	0.000000

30	H	0.000000	0.000000	0.000000	0.000000	0.000000	0.000001
31	H	0.000000	0.000000	0.000000	0.000000	0.000000	0.000000
32	H	0.000000	0.000000	0.000000	0.000000	0.000000	0.000000
33	H	0.000000	0.000000	0.000000	0.000000	0.000000	0.000000
34	H	-0.000001	-0.000006	0.000001	0.000003	-0.000056	-0.000310
35	H	0.000000	0.000000	0.000000	0.000000	0.000000	0.000000
36	H	0.005322	0.000108	0.000102	-0.000232	-0.000192	-0.000290
37	H	0.541856	-0.026048	-0.025268	-0.000332	0.000670	-0.000351
38	H	-0.026048	0.532075	-0.029265	-0.000078	0.002322	0.000193
39	H	-0.025268	-0.029265	0.529197	0.001350	-0.001294	0.000138
40	H	-0.000332	-0.000078	0.001350	0.526079	-0.028929	-0.025745
41	H	0.000670	0.002322	-0.001294	-0.028929	0.554422	-0.029124
42	H	-0.000351	0.000193	0.000138	-0.025745	-0.029124	0.532999

Mulliken charges:

		1
1	O	-0.536352
2	O	-0.522868
3	O	-0.562681
4	C	0.526153
5	C	-0.389033
6	C	-0.413348
7	C	0.278188
8	C	0.849558
9	C	-0.336672
10	C	-0.482128
11	C	-0.749571
12	C	-0.614177
13	C	-0.555031
14	C	0.708667
15	C	-0.323897
16	C	0.665760
17	C	-0.605294
18	C	-0.764824

19 H 0.123604

20 H 0.161839

21 H 0.139139

22 H 0.133713

23 H 0.107089

24 H 0.127992

25 H 0.112126

26 H 0.168778

27 H 0.177503

28 H 0.148429

29 H 0.136732

30 H 0.151634

31 H 0.152287

32 H 0.151423

33 H 0.158281

34 H 0.337647

35 H 0.309452

36 H 0.122389

37 H 0.135765

38 H 0.157693

39 H 0.159697

40 H 0.164658

41 H 0.137932

42 H 0.151748

Sum of Mulliken charges = 0.00000

Mulliken charges with hydrogens summed into heavy atoms:

1

1 O -0.198705

2 O -0.213417

3 O -0.562681

4 C 0.649757

5 C -0.088055

6 C -0.279634

7 C 0.385276

8 C 0.849558

9 C -0.208680

10 C -0.370001

11 C -0.403289

12 C -0.177381

13 C -0.093040

14 C 0.708667

15 C -0.201509

16 C 0.665760

17 C -0.152139

18 C -0.310486

Electronic spatial extent (au): <R**2>= 5778.2255

Charge= 0.0000 electrons

Dipole moment (field-independent basis, Debye):

X= -4.2517 Y= 0.2857 Z= -4.9320 Tot= 6.5179

Quadrupole moment (field-independent basis, Debye-Ang):

XX= -117.9421 YY= -107.4160 ZZ= -116.2838

XY= 13.8049 XZ= 13.8079 YZ= -2.0763

Traceless Quadrupole moment (field-independent basis, Debye-Ang):

XX= -4.0615 YY= 6.4647 ZZ= -2.4032

XY= 13.8049 XZ= 13.8079 YZ= -2.0763

Octapole moment (field-independent basis, Debye-Ang**2):

XXX= -106.8129 YYY= -1.6471 ZZZ= -15.3184 XYY= -17.7482

XXY= -23.6112 XXZ= 1.6391 XZZ= 4.9093 YZZ= -0.3132

YYZ= -13.9024 XYZ= 8.2931

Hexadecapole moment (field-independent basis, Debye-Ang**3):

XXXX= -5500.1927 YYYY= -1720.3221 ZZZZ= -675.7631 XXXY= 158.5093

XXXZ= 212.1613 YYYX= 17.4692 YYYZ= 26.5033 ZZZX= 10.9760

ZZZY= -9.1368 XXYY= -1154.6606 XXZZ= -1056.1510 YYZZ= -400.3736

XXYZ= -40.8630 YYXZ= 3.1461 ZZXY= 32.4505

N-N= 1.412129165660D+03 E-N=-4.716336842201D+03 KE= 8.083894541042D+02

1|1|UNPC-HORIZON|SP|RB3LYP|6-311+G(2d,p)|C15H24O3|USER|20-Dec-2025|0||

b3lyp/6-311+g(2d,p) scrf=(smd,solvent=water,read) geom=connectivity|

|MO||0,1|O,0,1.2533,2.2138,-0.3192|O,0,3.8828,0.2164,1.27|O,0,-2.3795,

-1.0169,-1.4099|C,0,0.6576,-0.7273,-0.4434|C,0,0.8391,0.1282,0.8257|C,

0,1.818,1.2879,0.6076|C,0,-0.0393,-2.0427,-0.0909|C,0,3.2036,0.7794,0.

1405|C,0,1.9625,-0.9862,-1.1503|C,0,3.0986,-0.3283,-0.8781|C,0,-1.3685

,-1.7944,0.6382|C,0,0.8815,-2.9023,0.7768|C,0,4.0725,1.919,-0.4031|C,0

,-2.3517,-0.977,-0.183|C,0,-3.3342,-0.1714,0.5972|C,0,-3.8617,0.9991,0

.1976|C,0,-4.8526,1.828,0.9663|C,0,-3.4821,1.5745,-1.1419|H,0,0.0386,-

0.1641,-1.1528|H,0,-0.1293,0.5566,1.1118|H,0,1.1927,-0.4515,1.6819|H,0

,1.9357,1.8227,1.5587|H,0,-0.2438,-2.5953,-1.0183|H,0,1.9762,-1.743,-1

.9308|H,0,3.9987,-0.6057,-1.4215|H,0,-1.2124,-1.323,1.6144|H,0,-1.8555

,-2.7585,0.8351|H,0,1.8528,-3.0958,0.3125|H,0,0.4168,-3.8846,0.9283|H,

0,1.0496,-2.4928,1.7763|H,0,4.1664,2.7189,0.3405|H,0,5.0923,1.572,-0.6

091|H,0,3.6655,2.3474,-1.3251|H,0,0.401,2.5108,0.0425|H,0,3.3881,-0.56

23,1.5764|H,0,-3.5867,-0.5635,1.5786|H,0,-5.1223,1.3502,1.9139|H,0,-4.

4328,2.8128,1.1954|H,0,-5.7707,1.9621,0.3854|H,0,-4.0954,1.1376,-1.936

3|H,0,-3.6496,2.6579,-1.1542|H,0,-2.4212,1.4412,-1.3752||Version=IA32W

-G09RevD.01|State=1-A|HF=-811.954292|RMSD=2.539e-009|Dipole=-1.6774006

,-0.0206951,1.9395312|Quadrupole=-3.3399398,5.0610241,-1.7210843,-10.4

488582,-9.9597915,-1.6049225|PG=C01 [X(C15H24O3)]||@

DON'T WORRY CHARLIE BROWN...WE LEARN MORE FROM LOSING THAN WE DO FROM WINNING.

THEN THAT MAKES ME THE SMARTEST PERSON IN THE WORLD...

CHARLES SCHULZ 'PEANUTS'

Job cpu time: 0 days 2 hours 1 minutes 25.0 seconds.

File lengths (MBytes): RWF=   329 Int=   0 D2E=   0 Chk=   15 Scr=   1

Normal termination of Gaussian 09 at Sat Dec 20 01:06:19 2025.

S3: Supplementary Table S1: RMSF Values Profile of COX-1 Receptor complex

Residue No.	Chain	RMSF Value
**32**	A	3.229
**33**	A	1.976
**34**	A	0.964
**35**	A	0.654
**36**	A	0.573
**37**	A	0.555
**38**	A	0.54
**39**	A	0.298
**40**	A	0.355
**41**	A	0.313
**42**	A	0.538
**43**	A	0.56
**44**	A	0.518
**45**	A	0.478
**46**	A	0.44
**47**	A	0.247
**48**	A	0.761
**49**	A	0.643
**50**	A	1.123
**51**	A	2.283
**52**	A	3.646
**53**	A	3.508
**54**	A	0.504
**55**	A	0.329
**56**	A	0.354
**57**	A	0.286
**58**	A	0.266
**59**	A	0.383
**60**	A	0.649
**61**	A	1.137
**62**	A	1.475
**63**	A	1.49
**64**	A	1.113
**65**	A	1.396
**66**	A	1.333
**67**	A	1.726
**68**	A	1.524
**69**	A	0.83
**70**	A	0.919
**71**	A	0.799

**72**	A	1.052
**73**	A	1.267
**74**	A	1.466
**75**	A	1.375
**76**	A	1.401
**77**	A	1.458
**78**	A	1.423
**79**	A	1.473
**80**	A	1.575
**81**	A	1.586
**82**	A	1.427
**83**	A	1.154
**84**	A	1.178
**85**	A	0.867
**86**	A	0.753
**87**	A	0.493
**88**	A	0.484
**89**	A	0.55
**90**	A	0.487
**91**	A	0.446
**92**	A	0.663
**93**	A	0.639
**94**	A	0.443
**95**	A	0.5
**96**	A	0.415
**97**	A	0.488
**98**	A	0.44
**99**	A	0.414
**100**	A	0.389
**101**	A	0.392
**102**	A	0.451
**103**	A	0.531
**104**	A	0.313
**105**	A	0.661
**106**	A	0.668
**107**	A	0.668
**108**	A	0.303
**109**	A	0.322
**110**	A	0.326
**111**	A	0.348
**112**	A	0.327
**113**	A	0.393
**114**	A	0.362

**115**	A	0.438
**116**	A	0.368
**117**	A	0.412
**118**	A	0.553
**119**	A	0.577
**120**	A	0.421
**121**	A	0.457
**122**	A	0.509
**123**	A	0.674
**124**	A	0.889
**125**	A	0.926
**126**	A	1.645
**127**	A	1.315
**128**	A	1.07
**129**	A	0.886
**130**	A	0.541
**131**	A	0.356
**132**	A	0.716
**133**	A	1.485
**134**	A	1.034
**135**	A	0.97
**136**	A	1.162
**137**	A	1.637
**138**	A	1.498
**139**	A	1.765
**140**	A	1.977
**141**	A	1.369
**142**	A	1.689
**143**	A	1.971
**144**	A	1.691
**145**	A	1.791
**146**	A	0.726
**147**	A	0.377
**148**	A	0.306
**149**	A	0.293
**150**	A	0.434
**151**	A	0.369
**152**	A	0.355
**153**	A	0.565
**154**	A	0.692
**155**	A	0.885
**156**	A	0.946
**157**	A	1.654

**158**	A	1.994
**159**	A	1.404
**160**	A	1.073
**161**	A	1.193
**162**	A	1.168
**163**	A	0.69
**164**	A	0.673
**165**	A	0.728
**166**	A	1.013
**167**	A	1.353
**168**	A	0.898
**169**	A	1.682
**170**	A	1.029
**171**	A	1.619
**172**	A	1.099
**173**	A	0.648
**174**	A	0.542
**175**	A	0.515
**176**	A	0.704
**177**	A	0.504
**178**	A	0.454
**179**	A	0.542
**180**	A	0.627
**181**	A	0.509
**182**	A	0.968
**183**	A	0.725
**184**	A	1.055
**185**	A	1.923
**186**	A	2.576
**187**	A	2.251
**188**	A	2.559
**189**	A	2.461
**190**	A	3.327
**191**	A	3.001
**192**	A	1.876
**193**	A	1.166
**194**	A	0.459
**195**	A	0.337
**196**	A	0.38
**197**	A	0.108
**198**	A	0.178
**199**	A	0.104
**200**	A	0.145

**201**	A	0.259
**202**	A	0.313
**203**	A	0.571
**204**	A	0.617
**205**	A	0.802
**206**	A	1.104
**207**	A	0.96
**208**	A	0.929
**209**	A	0.87
**210**	A	0.724
**211**	A	0.7
**212**	A	1.112
**213**	A	1.052
**214**	A	1.35
**215**	A	2.124
**216**	A	3.608
**217**	A	3.169
**218**	A	1.768
**219**	A	1.007
**220**	A	0.661
**221**	A	0.538
**222**	A	0.521
**223**	A	0.832
**224**	A	1.868
**225**	A	2.266
**226**	A	1.985
**227**	A	1.156
**228**	A	0.753
**229**	A	0.906
**230**	A	0.805
**231**	A	1.178
**232**	A	0.968
**233**	A	0.645
**234**	A	1.16
**235**	A	1.519
**236**	A	1.322
**237**	A	1.607
**238**	A	1.619
**239**	A	1.462
**240**	A	1.345
**241**	A	1.399
**242**	A	1.115
**243**	A	1.333

**244**	A	1.497
**245**	A	0.688
**246**	A	1.853
**247**	A	3.413
**248**	A	3.48
**249**	A	2.849
**250**	A	2.513
**251**	A	1.224
**252**	A	0.596
**253**	A	1.048
**254**	A	0.683
**255**	A	0.533
**256**	A	0.743
**257**	A	0.98
**258**	A	1.864
**259**	A	1.673
**260**	A	0.757
**261**	A	0.628
**262**	A	0.392
**263**	A	0.496
**264**	A	0.43
**265**	A	0.711
**266**	A	0.871
**267**	A	1.681
**268**	A	2.434
**269**	A	2.196
**270**	A	1.894
**271**	A	1.422
**272**	A	1.826
**273**	A	1.415
**274**	A	1.189
**275**	A	0.841
**276**	A	0.932
**277**	A	0.917
**278**	A	1.268
**279**	A	1.421
**280**	A	1.795
**281**	A	1.525
**282**	A	1.462
**283**	A	1.08
**284**	A	0.938
**285**	A	0.678
**286**	A	0.446

**287**	A	0.917
**288**	A	0.863
**289**	A	0.807
**290**	A	1.068
**291**	A	1.71
**292**	A	1.757
**293**	A	1.457
**294**	A	0.654
**295**	A	0.549
**296**	A	0.19
**297**	A	0.145
**298**	A	0.207
**299**	A	0.208
**300**	A	0.293
**301**	A	0.407
**302**	A	0.427
**303**	A	0.206
**304**	A	0.144
**305**	A	0.191
**306**	A	0.18
**307**	A	0.292
**308**	A	0.154
**309**	A	0.225
**310**	A	0.231
**311**	A	0.379
**312**	A	0.315
**313**	A	0.535
**314**	A	0.45
**315**	A	0.392
**316**	A	0.503
**317**	A	0.672
**318**	A	0.845
**319**	A	0.822
**320**	A	1.252
**321**	A	2.725
**322**	A	2.79
**323**	A	1.391
**324**	A	1.122
**325**	A	0.78
**326**	A	0.731
**327**	A	0.714
**328**	A	0.404
**329**	A	0.519

**330**	A	0.52
**331**	A	0.564
**332**	A	0.531
**333**	A	0.479
**334**	A	0.372
**335**	A	0.38
**336**	A	0.381
**337**	A	0.351
**338**	A	0.315
**339**	A	0.15
**340**	A	0.134
**341**	A	0.336
**342**	A	0.31
**343**	A	0.464
**344**	A	0.271
**345**	A	0.393
**346**	A	0.249
**347**	A	0.348
**348**	A	0.209
**349**	A	0.446
**350**	A	0.527
**351**	A	0.682
**352**	A	0.337
**353**	A	0.699
**354**	A	0.723
**355**	A	0.805
**356**	A	0.636
**357**	A	0.969
**358**	A	1.007
**359**	A	0.603
**360**	A	0.641
**361**	A	0.907
**362**	A	1.013
**363**	A	1.417
**364**	A	1.133
**365**	A	0.81
**366**	A	1.018
**367**	A	1.022
**368**	A	1.819
**369**	A	1.945
**370**	A	1.548
**371**	A	1.849
**372**	A	1.942

**373**	A	1.756
**374**	A	1.857
**375**	A	1.086
**376**	A	1.359
**377**	A	0.741
**378**	A	0.179
**379**	A	0.289
**380**	A	0.185
**381**	A	0.522
**382**	A	0.348
**383**	A	0.132
**384**	A	0.376
**385**	A	0.459
**386**	A	0.541
**387**	A	1.367
**388**	A	1.314
**389**	A	0.892
**390**	A	1.133
**391**	A	1.323
**392**	A	0.889
**393**	A	0.995
**394**	A	0.958
**395**	A	0.754
**396**	A	0.853
**397**	A	1.08
**398**	A	1.577
**399**	A	1.762
**400**	A	1.171
**401**	A	0.817
**402**	A	0.735
**403**	A	0.784
**404**	A	0.828
**405**	A	0.933
**406**	A	0.913
**407**	A	0.78
**408**	A	0.944
**409**	A	0.976
**410**	A	0.897
**411**	A	0.73
**412**	A	0.557
**413**	A	0.528
**414**	A	0.654
**415**	A	0.577

**416**	A	0.74
**417**	A	0.549
**418**	A	0.475
**419**	A	0.446
**420**	A	0.409
**421**	A	0.421
**422**	A	0.354
**423**	A	0.54
**424**	A	0.255
**425**	A	0.384
**426**	A	0.176
**427**	A	0.295
**428**	A	0.5
**429**	A	0.718
**430**	A	0.464
**431**	A	0.792
**432**	A	1.404
**433**	A	1.214
**434**	A	1.624
**435**	A	2.31
**436**	A	2.134
**437**	A	1.276
**438**	A	1.476
**439**	A	0.784
**440**	A	0.812
**441**	A	0.737
**442**	A	1.208
**443**	A	1.515
**444**	A	0.967
**445**	A	0.631
**446**	A	0.637
**447**	A	0.623
**448**	A	0.567
**449**	A	0.263
**450**	A	0.257
**451**	A	0.207
**452**	A	0.122
**453**	A	0.134
**454**	A	0.329
**455**	A	0.129
**456**	A	0.14
**457**	A	0.302
**458**	A	0.204

**459**	A	0.218
**460**	A	0.304
**461**	A	0.234
**462**	A	0.238
**463**	A	0.161
**464**	A	0.167
**465**	A	0.436
**466**	A	0.547
**467**	A	0.382
**468**	A	0.363
**469**	A	0.461
**470**	A	0.637
**471**	A	0.623
**472**	A	0.908
**473**	A	0.974
**474**	A	1.65
**475**	A	1.627
**476**	A	1.296
**477**	A	0.85
**478**	A	0.307
**479**	A	0.542
**480**	A	0.716
**481**	A	0.482
**482**	A	0.562
**483**	A	0.807
**484**	A	1.047
**485**	A	0.982
**486**	A	1.041
**487**	A	0.915
**488**	A	0.777
**489**	A	0.842
**490**	A	0.818
**491**	A	0.751
**492**	A	0.668
**493**	A	0.892
**494**	A	1.098
**495**	A	0.979
**496**	A	1.067
**497**	A	0.862
**498**	A	0.39
**499**	A	0.44
**500**	A	0.492
**501**	A	0.167

**502**	A	0.327
**503**	A	0.117
**504**	A	0.11
**505**	A	0.122
**506**	A	0.115
**507**	A	0.23
**508**	A	0.184
**509**	A	0.183
**510**	A	0.331
**511**	A	0.785
**512**	A	2.523
**513**	A	2.605
**514**	A	2.574
**515**	A	2.432
**516**	A	2.315
**517**	A	0.801
**518**	A	0.718
**519**	A	0.707
**520**	A	0.694
**521**	A	0.491
**522**	A	0.54
**523**	A	0.618
**524**	A	0.387
**525**	A	0.275
**526**	A	0.469
**527**	A	0.401
**528**	A	0.498
**529**	A	0.488
**530**	A	0.398
**531**	A	0.385
**532**	A	0.396
**533**	A	0.494
**534**	A	0.519
**535**	A	0.643
**536**	A	0.797
**537**	A	0.865
**538**	A	1.031
**539**	A	0.736
**540**	A	0.878
**541**	A	1.288
**542**	A	1.377
**543**	A	1.899
**544**	A	1.567

**545**	A	1.106
**546**	A	1.997
**547**	A	1.991
**548**	A	1.772
**549**	A	1.509
**550**	A	0.814
**551**	A	1.964
**552**	A	1.261
**553**	A	0.838
**554**	A	0.787
**555**	A	0.745
**556**	A	0.634
**557**	A	0.628
**558**	A	0.532
**559**	A	0.403
**560**	A	0.585
**561**	A	0.797
**562**	A	0.867
**563**	A	0.837
**564**	A	0.776
**565**	A	0.676
**566**	A	0.545
**567**	A	0.51
**568**	A	0.753
**569**	A	0.734
**570**	A	0.786
**571**	A	1.018
**572**	A	1.032
**573**	A	1.07
**574**	A	1.568
**575**	A	1.362
**576**	A	1.74
**577**	A	1.888
**578**	A	1.157
**579**	A	1.061
**580**	A	0.768
**581**	A	0.701
**582**	A	1.226
**583**	A	2.078
**584**	A	3.335

S4: Supplementary Table 2: RMSF Values Profile of IKKβ Receptor complex

**1**	**A**	**2.013**
**2**	A	1.593
**3**	A	0.815
**4**	A	1.459
**5**	A	1.76
**6**	A	1.625
**7**	A	1.99
**8**	A	1.36
**9**	A	1.044
**10**	A	0.648
**11**	A	0.414
**12**	A	0.476
**13**	A	0.774
**14**	A	0.581
**15**	A	0.185
**16**	A	0.161
**17**	A	0.102
**18**	A	0.173
**19**	A	0.361
**20**	A	0.696
**21**	A	0.925
**22**	A	1.144
**23**	A	1.233
**24**	A	1.511
**25**	A	1.279
**26**	A	1.365
**27**	A	1.207
**28**	A	0.829
**29**	A	0.506
**30**	A	0.325
**31**	A	0.131
**32**	A	0.091
**33**	A	0.168
**34**	A	0.46
**35**	A	0.827
**36**	A	1.308
**37**	A	1.215
**38**	A	0.965
**39**	A	0.555
**40**	A	0.473
**41**	A	0.471

**42**	A	0.1
**43**	A	0.09
**44**	A	0.49
**45**	A	0.669
**46**	A	1.46
**47**	A	2.018
**48**	A	3.053
**49**	A	1.962
**50**	A	1.799
**51**	A	1.345
**52**	A	0.959
**53**	A	0.851
**54**	A	0.856
**55**	A	0.721
**56**	A	0.734
**57**	A	0.718
**58**	A	0.616
**59**	A	0.631
**60**	A	0.528
**61**	A	0.292
**62**	A	0.293
**63**	A	0.231
**64**	A	0.143
**65**	A	0.098
**66**	A	0.342
**67**	A	0.302
**68**	A	0.419
**69**	A	0.839
**70**	A	0.707
**71**	A	1.597
**72**	A	0.784
**73**	A	0.614
**74**	A	0.529
**75**	A	0.617
**76**	A	0.363
**77**	A	0.388
**78**	A	0.717
**79**	A	0.785
**80**	A	1.052
**81**	A	1.421
**82**	A	2.62
**83**	A	2.563
**84**	A	2.07

**85**	A	2.841
**86**	A	1.667
**87**	A	1.841
**88**	A	2.162
**89**	A	3.076
**90**	A	2.078
**91**	A	1.63
**92**	A	1.186
**93**	A	0.581
**94**	A	0.097
**95**	A	0.092
**96**	A	0.191
**97**	A	0.305
**98**	A	0.477
**99**	A	0.508
**100**	A	0.565
**101**	A	0.534
**102**	A	0.535
**103**	A	0.421
**104**	A	0.483
**105**	A	0.666
**106**	A	0.637
**107**	A	0.527
**108**	A	0.721
**109**	A	0.934
**110**	A	0.751
**111**	A	0.818
**112**	A	0.925
**113**	A	1.044
**114**	A	1.244
**115**	A	1.81
**116**	A	2.279
**117**	A	0.921
**118**	A	0.658
**119**	A	0.566
**120**	A	0.893
**121**	A	0.561
**122**	A	0.248
**123**	A	0.224
**124**	A	0.4
**125**	A	0.156
**126**	A	0.175
**127**	A	0.15

**128**	A	0.074
**129**	A	0.108
**130**	A	0.214
**131**	A	0.137
**132**	A	0.281
**133**	A	0.242
**134**	A	0.238
**135**	A	0.221
**136**	A	0.173
**137**	A	0.16
**138**	A	0.409
**139**	A	0.79
**140**	A	0.483
**141**	A	0.36
**142**	A	0.19
**143**	A	0.279
**144**	A	0.469
**145**	A	0.556
**146**	A	0.506
**147**	A	0.663
**148**	A	0.608
**149**	A	0.509
**150**	A	0.489
**151**	A	0.403
**152**	A	0.321
**153**	A	0.305
**154**	A	0.222
**155**	A	0.395
**156**	A	0.818
**157**	A	0.899
**158**	A	0.814
**159**	A	0.689
**160**	A	0.407
**161**	A	0.173
**162**	A	0.213
**163**	A	0.182
**164**	A	0.473
**165**	A	0.561
**166**	A	0.59
**167**	A	0.369
**168**	A	0.615
**169**	A	0.669
**170**	A	0.695

**171**	A	0.308
**172**	A	0.503
**173**	A	0.635
**177**	A	2.09
**178**	A	0.293
**179**	A	0.307
**180**	A	0.467
**181**	A	1.033
**182**	A	1.771
**183**	A	1.275
**184**	A	1.639
**185**	A	1.343
**186**	A	0.908
**187**	A	0.849
**188**	A	0.625
**189**	A	0.494
**190**	A	0.683
**191**	A	0.688
**192**	A	0.748
**193**	A	0.914
**194**	A	0.946
**195**	A	1.122
**196**	A	1.124
**197**	A	0.976
**198**	A	0.95
**199**	A	0.438
**200**	A	0.316
**201**	A	0.444
**202**	A	0.556
**203**	A	0.366
**204**	A	0.248
**205**	A	0.128
**206**	A	0.259
**207**	A	0.258
**208**	A	0.111
**209**	A	0.288
**210**	A	0.294
**211**	A	0.472
**212**	A	0.385
**213**	A	0.313
**214**	A	0.273
**215**	A	0.452
**216**	A	0.513

**217**	A	0.665
**218**	A	0.911
**219**	A	0.899
**220**	A	1.022
**221**	A	0.523
**222**	A	0.142
**223**	A	0.779
**224**	A	0.952
**225**	A	1.318
**226**	A	1.087
**227**	A	1.059
**228**	A	0.74
**229**	A	0.981
**230**	A	0.846
**231**	A	0.504
**232**	A	0.577
**233**	A	0.87
**234**	A	0.674
**235**	A	0.634
**236**	A	0.861
**237**	A	0.898
**238**	A	1.152
**239**	A	1.2
**240**	A	1.007
**241**	A	0.547
**242**	A	1.1
**243**	A	0.804
**244**	A	0.62
**245**	A	0.372
**246**	A	0.545
**247**	A	0.771
**248**	A	0.95
**249**	A	1.498
**250**	A	1.636
**251**	A	2.587
**252**	A	1.777
**253**	A	0.944
**254**	A	0.89
**255**	A	0.986
**256**	A	1.011
**257**	A	1.651
**258**	A	1.933
**259**	A	1.082

**260**	A	1.793
**261**	A	1.292
**262**	A	2.481
**263**	A	1.533
**264**	A	1.499
**265**	A	1.01
**266**	A	0.736
**267**	A	0.583
**268**	A	0.845
**269**	A	0.551
**270**	A	0.475
**271**	A	0.313
**272**	A	0.328
**273**	A	0.122
**274**	A	0.124
**275**	A	0.231
**276**	A	0.379
**277**	A	0.19
**278**	A	0.235
**279**	A	0.216
**280**	A	0.265
**281**	A	0.332
**282**	A	0.594
**283**	A	0.504
**284**	A	0.838
**285**	A	1.226
**286**	A	1.311
**287**	A	1.732
**288**	A	2.449
**289**	A	2.016
**290**	A	1.712
**291**	A	1.287
**292**	A	2.344
**293**	A	1.698
**294**	A	1.591
**295**	A	1.841
**296**	A	1.781
**297**	A	1.233
**298**	A	1.042
**299**	A	0.487
**300**	A	0.139
**301**	A	0.157
**302**	A	0.226

**303**	A	0.171
**304**	A	0.196
**305**	A	0.399
**306**	A	0.252
**307**	A	0.48
**308**	A	0.718
**309**	A	0.606
**310**	A	0.622
**311**	A	0.637
**312**	A	0.423
**313**	A	0.305
**314**	A	0.12
**315**	A	0.129
**316**	A	0.366
**317**	A	0.561
**318**	A	1.223
**319**	A	1.805
**320**	A	0.81
**321**	A	0.573
**322**	A	0.461
**323**	A	0.442
**324**	A	0.503
**325**	A	0.406
**326**	A	0.584
**327**	A	0.712
**328**	A	1.428
**329**	A	2.521
**330**	A	2.515
**331**	A	1.321
**332**	A	0.902
**333**	A	0.629
**334**	A	0.651
**335**	A	0.771
**336**	A	0.487
**337**	A	0.318
**338**	A	0.458
**339**	A	0.389
**340**	A	0.375
**341**	A	0.535
**342**	A	0.522
**343**	A	0.726
**344**	A	0.944
**345**	A	1.99

**346**	A	1.625
**347**	A	1.449
**348**	A	1.363
**349**	A	1.359
**350**	A	0.831
**351**	A	0.439
**352**	A	0.279
**353**	A	0.429
**354**	A	0.278
**355**	A	0.59
**356**	A	0.738
**357**	A	1.18
**358**	A	1.305
**359**	A	1.014
**360**	A	0.655
**361**	A	0.935
**362**	A	1.95
**363**	A	2.541
**364**	A	2.148
**365**	A	1.442
**366**	A	1.115
**367**	A	0.75
**368**	A	0.853
**369**	A	0.952
**370**	A	0.718
**371**	A	1.626
**372**	A	2.98
**376**	A	2.078
**377**	A	1.774
**378**	A	2.156
**379**	A	2.532
**380**	A	1.97
**381**	A	2.619
**382**	A	2.753
**383**	A	1.729
**384**	A	1.222
**385**	A	0.536
**386**	A	0.441
**387**	A	0.067
**388**	A	0.064
**389**	A	0.289
**390**	A	0.567
**391**	A	0.691

**392**	A	0.963
**393**	A	1.514
**394**	A	1.355
**395**	A	1.457
**396**	A	1.452
**397**	A	1.33
**398**	A	1.715
**399**	A	2.657
**400**	A	2.348
**401**	A	1.982
**402**	A	2.37
**403**	A	1.05
**404**	A	1.117
**405**	A	1.166
**406**	A	0.963
**407**	A	0.982
**408**	A	0.993
**409**	A	0.801
**410**	A	0.743
**411**	A	0.857
**412**	A	0.882
**413**	A	0.787
**414**	A	0.918
**415**	A	1.003
**416**	A	1.743
**417**	A	2.497
**418**	A	2.137
**419**	A	2.69
**420**	A	2.374
**421**	A	1.255
**422**	A	0.959
**423**	A	0.715
**424**	A	0.706
**425**	A	0.609
**426**	A	0.447
**427**	A	0.444
**428**	A	0.494
**429**	A	0.394
**430**	A	0.595
**431**	A	0.586
**432**	A	0.394
**433**	A	0.364
**434**	A	0.248

**435**	A	0.406
**436**	A	0.185
**437**	A	0.196
**438**	A	0.073
**439**	A	0.146
**440**	A	0.258
**441**	A	0.147
**442**	A	0.242
**443**	A	0.216
**444**	A	0.206
**445**	A	0.383
**446**	A	0.255
**447**	A	0.205
**448**	A	0.3
**449**	A	0.223
**450**	A	0.442
**451**	A	0.403
**452**	A	0.385
**453**	A	0.335
**454**	A	0.402
**455**	A	0.381
**456**	A	0.356
**457**	A	0.327
**458**	A	0.35
**459**	A	0.352
**460**	A	0.469
**461**	A	0.17
**462**	A	0.157
**463**	A	0.281
**464**	A	0.309
**465**	A	0.19
**466**	A	0.157
**467**	A	0.283
**468**	A	0.189
**469**	A	0.124
**470**	A	0.519
**471**	A	0.535
**472**	A	0.405
**473**	A	0.589
**474**	A	0.561
**475**	A	0.386
**476**	A	0.546
**477**	A	0.509

**478**	A	0.44
**479**	A	0.254
**480**	A	0.439
**481**	A	0.409
**482**	A	0.231
**483**	A	0.248
**484**	A	0.206
**485**	A	0.182
**486**	A	0.248
**487**	A	0.227
**488**	A	0.293
**489**	A	0.324
**490**	A	0.222
**491**	A	0.486
**492**	A	0.561
**493**	A	0.502
**494**	A	0.285
**495**	A	0.727
**496**	A	0.818
**497**	A	0.761
**498**	A	0.959
**499**	A	1.366
**500**	A	1.526
**501**	A	1.737
**502**	A	1.9
**503**	A	1.499
**504**	A	1.756
**505**	A	1.66
**506**	A	1.951
**507**	A	1.659
**508**	A	1.226
**509**	A	1.06
**510**	A	0.688
**511**	A	0.749
**512**	A	0.637
**513**	A	0.266
**514**	A	0.196
**515**	A	0.38
**516**	A	0.456
**517**	A	0.354
**518**	A	0.386
**519**	A	0.427
**520**	A	0.757

**521**	A	0.628
**522**	A	0.682
**523**	A	0.636
**524**	A	1.223
**525**	A	1.499
**526**	A	0.959
**527**	A	0.788
**528**	A	0.68
**529**	A	0.675
**530**	A	0.419
**531**	A	0.497
**532**	A	0.277
**533**	A	0.187
**534**	A	0.198
**535**	A	0.175
**536**	A	0.165
**537**	A	0.15
**538**	A	0.343
**539**	A	0.162
**540**	A	0.17
**541**	A	0.357
**542**	A	0.087
**543**	A	0.128
**544**	A	0.08
**545**	A	0.082
**546**	A	0.101
**547**	A	0.303
**548**	A	0.337
**549**	A	0.694
**550**	A	1.108
**558**	A	1.109
**559**	A	0.562
**560**	A	0.333
**561**	A	0.554
**562**	A	0.314
**563**	A	0.226
**564**	A	0.164
**565**	A	0.21
**566**	A	0.113
**567**	A	0.109
**568**	A	0.097
**569**	A	0.11
**570**	A	0.413

**571**	A	0.293
**572**	A	0.469
**573**	A	0.584
**574**	A	0.756
**575**	A	0.852
**576**	A	1.359
**577**	A	1.871
**578**	A	3.09
**579**	A	2.885
**580**	A	2.691
**581**	A	3.318
**582**	A	3.166
**583**	A	3.56
**584**	A	2.275
**585**	A	1.514
**586**	A	1.246
**587**	A	0.8
**588**	A	0.481
**589**	A	0.352
**590**	A	0.467
**591**	A	0.502
**592**	A	0.44
**593**	A	0.417
**594**	A	0.33
**595**	A	0.421
**596**	A	0.404
**597**	A	0.398
**598**	A	0.449
**599**	A	0.449
**600**	A	0.418
**601**	A	0.488
**602**	A	0.632
**603**	A	0.576
**604**	A	0.445
**605**	A	0.424
**606**	A	0.292
**607**	A	0.32
**608**	A	0.332
**609**	A	0.291
**610**	A	0.391
**611**	A	0.267
**612**	A	0.216
**613**	A	0.07

**614**	A	0.072
**615**	A	0.068
**616**	A	0.068
**617**	A	0.074
**618**	A	0.073
**619**	A	0.069
**620**	A	0.074
**621**	A	0.077
**622**	A	0.075
**623**	A	0.075
**624**	A	0.1
**625**	A	0.109
**626**	A	0.125
**627**	A	0.253
**628**	A	0.184
**629**	A	0.134
**630**	A	0.431
**631**	A	0.418
**632**	A	0.333
**633**	A	0.481
**634**	A	0.482
**635**	A	0.42
**636**	A	0.372
**637**	A	0.384
**638**	A	0.453
**639**	A	0.499
**640**	A	0.364
**641**	A	0.394
**642**	A	0.464
**643**	A	0.345
**644**	A	0.472
**645**	A	0.174
**646**	A	0.177
**647**	A	0.17
**648**	A	0.182
**649**	A	0.192
**650**	A	0.175
**651**	A	0.359
**652**	A	0.557
**653**	A	0.444
**654**	A	0.497
**655**	A	0.49
**656**	A	0.454

**657**	A	0.445
**658**	A	0.685
**659**	A	0.715
**660**	A	0.595
**661**	A	0.675
**662**	A	1.468
**663**	A	2.71
